# Carbon dots as probes in FLIM:a review of applications and advances in cellular imaging

**DOI:** 10.1039/d5ra05371d

**Published:** 2025-11-18

**Authors:** Fozia Aslam, Jiaqing Guo, Asif Khalid, Saad Anwar, Kinza Arshad, Muhammad Nouman Khan, Puxiang Lai, Liwei Liu

**Affiliations:** a Center for Biomedical Optics and Photonics (CBOP), College of Physics and Optoelectronic Engineering, Key Laboratory of Optoelectronic Devices and Systems, Shenzhen University Shenzhen 518060 P. R. China liulw@szu.edu.cn; b THz Technology Laboratory, Shenzhen Key Laboratory of Micro-nano Photonic Information Technology, Shenzhen University Shenzhen 518060 China; c College of Physics and Optoelectronic Engineering, Shenzhen University Shenzhen 518061 People's Republic of China; d Department of Biomedical Engineering, Hong Kong Polytechnic University Hong Kong

## Abstract

Carbon dots (CDs) have emerged as an exceptional alternative to traditional fluorescent probes for cellular imaging due to their high brightness, photostability, tunable fluorescence emission, and low toxicity. These properties make CDs ideal for applications requiring high sensitivity and minimal phototoxicity. However, their optimal potential is realized when combined with advanced fluorescence imaging techniques like fluorescence lifetime imaging microscopy (FLIM). FLIM provides deep insights into dynamic cellular behaviors by measuring fluorescence lifetimes, offering information that traditional intensity-based methods cannot measure. Thus, this review explores the optical properties and fluorescence mechanisms of CDs. The use of UV-vis absorption and photoluminescence (PL) spectroscopy is also discussed to illustrate the unique behavior of CDs. Their applications in cellular imaging, including organelle visualization and real-time tracking of intracellular processes, are examined. The combination of CDs with FLIM enhances the sensitivity of cellular imaging, enabling label-free, time-resolved measurements of cellular dynamics. This integration allows for precise monitoring of metabolic shifts, molecular interactions, and bacterial detection *via* multicolor imaging. Finally, we addressed the challenges and future directions in optimizing CDs and FLIM, with a focus on improving temporal and spatial resolution. The combination of CDs and FLIM holds transformative potential for advancing biomedical diagnostics and therapeutic monitoring.

## Abstract 2

The pictorial representation of the paper.

## Graphical abstract

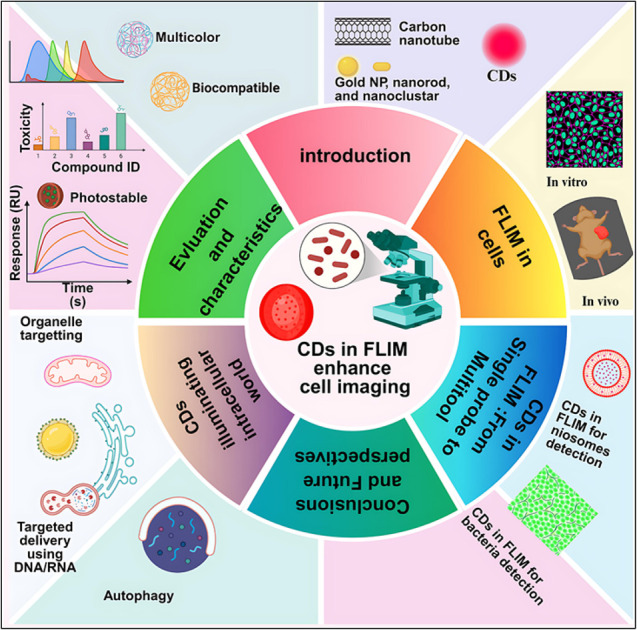

## 1. Introduction

**Fig. 1:**
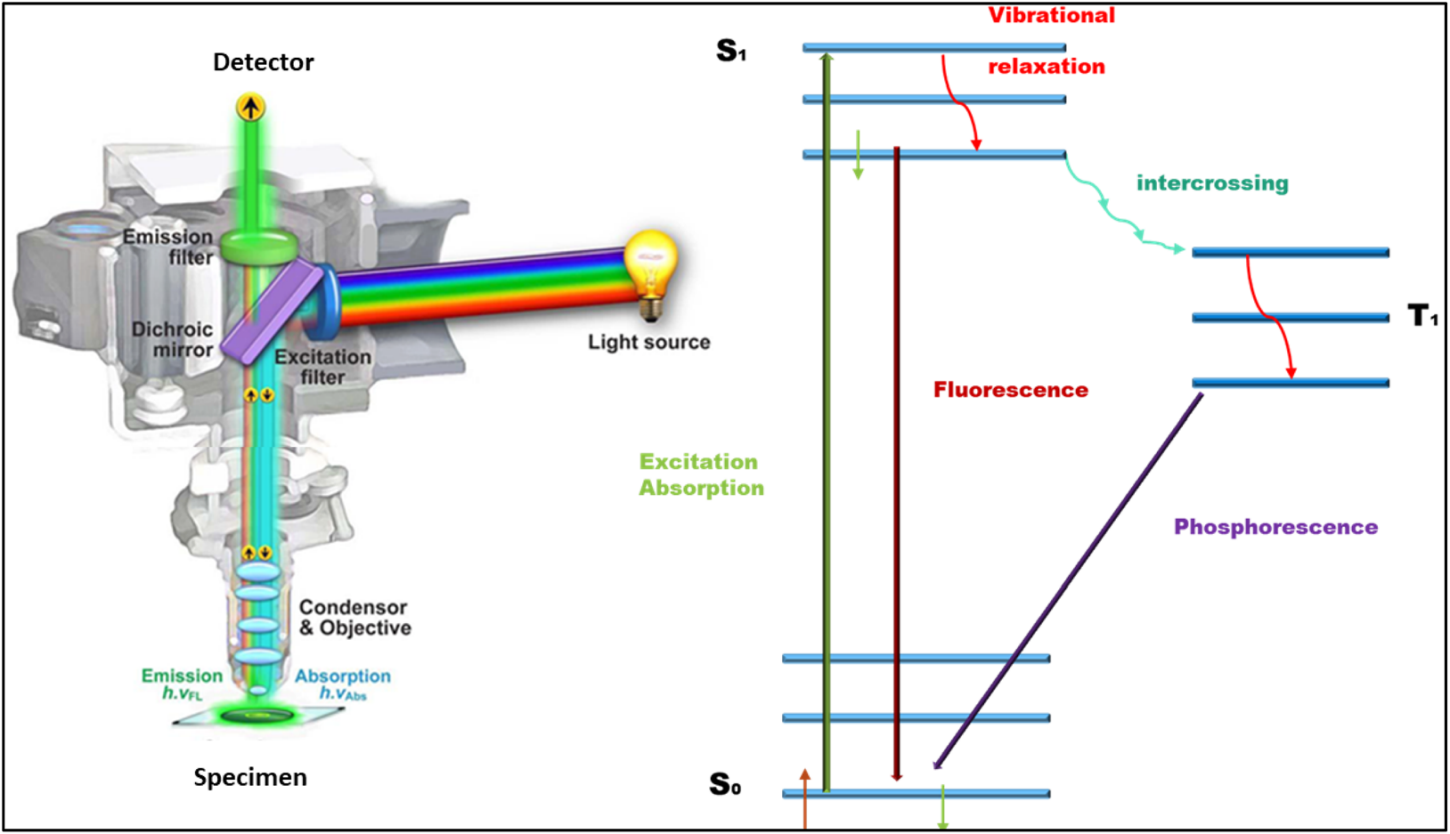
Illustration of the fluorescence microscopy, and phosphorescence. (Adapted with permission from ref. 11 Copyrights 2012, MDPI). Jablonski diagram of fluorescence.

Cell imaging has revolutionized biological research by enabling real-time visualization of dynamic molecular processes with unprecedented spatiotemporal resolution.^1,2^ This technique focuses on observing the structures and activities within living cells, providing high-resolution images of processes such as cell development, migration, and intracellular transport. The cell imaging techniques rely on the alteration between electromagnetic wavelengths and cellular structure.^3^ The non-invasive imaging techniques (like fluorescence microscopy) are increasingly common for examining live cells and identifying specific targets.^4^ The photophysics of fluorescence imaging is based on three steps: firstly, specific wavelength photons excite fluorophores to higher electronic states (S_0_ → S_1_), then non-radiative relaxation occurs through vibrational states.^5^ Finally, during the electronics transition (S_1_ → S_0_), longer-wavelength photons are emitted (Fig. 1). These principles have driven the development of advanced imaging methods that help to overcome diffraction limits, achieving nanometer-scale resolution approximately less than 20 nm (nanometer). Super-resolution techniques, such as STED microscopy (which uses patterned excitation) and STORM (based on stochastic fluorophore switching), exemplify these advancements.^6–8^ Additionally, near infrared (NIR-II) imaging (operating between 1000–1700 nm) allows deeper tissue penetration by minimizing photon scattering and autofluorescence.^9,10^

Despite these innovations, early detection of diseased cells remains challenging due to insufficient monitoring and diagnostic methods, often leading to delayed or false diagnoses.^12,13^ Diseased cells exhibit complex biological structures, including microscopic entities like ions and biomolecules *e.g.*, reactive oxygen and sulfur species (ROS), as well as microenvironmental factors such as pH, viscosity, oxygen levels, and molecular markers (*e.g.*, peptides, proteins, and nucleic acids).^14^ These factors play critical roles in cancer progression, drug resistance, and metastasis. For instance, cancer cells often exhibit extracellular pH downregulation and glutathione (GSH) overexpression.^15–17^ Although these changes at the cellular and molecular levels are very important in disease, but traditional clinical imaging techniques like CT, PET, MRI, and ultrasound aren't often sensitive enough to detect early biochemical and microenvironmental changes.^18–20^ Moreover, some techniques (*e.g.*, CT and PET) carry radiological risks, further limiting their suitability for repeated or high-resolution monitoring.^19^ Therefore, it is imperative to develop cutting-edge imaging technologies that are capable of accurately and safely diagnosing these subliminal diagnostic changes.

**Fig. 2:**
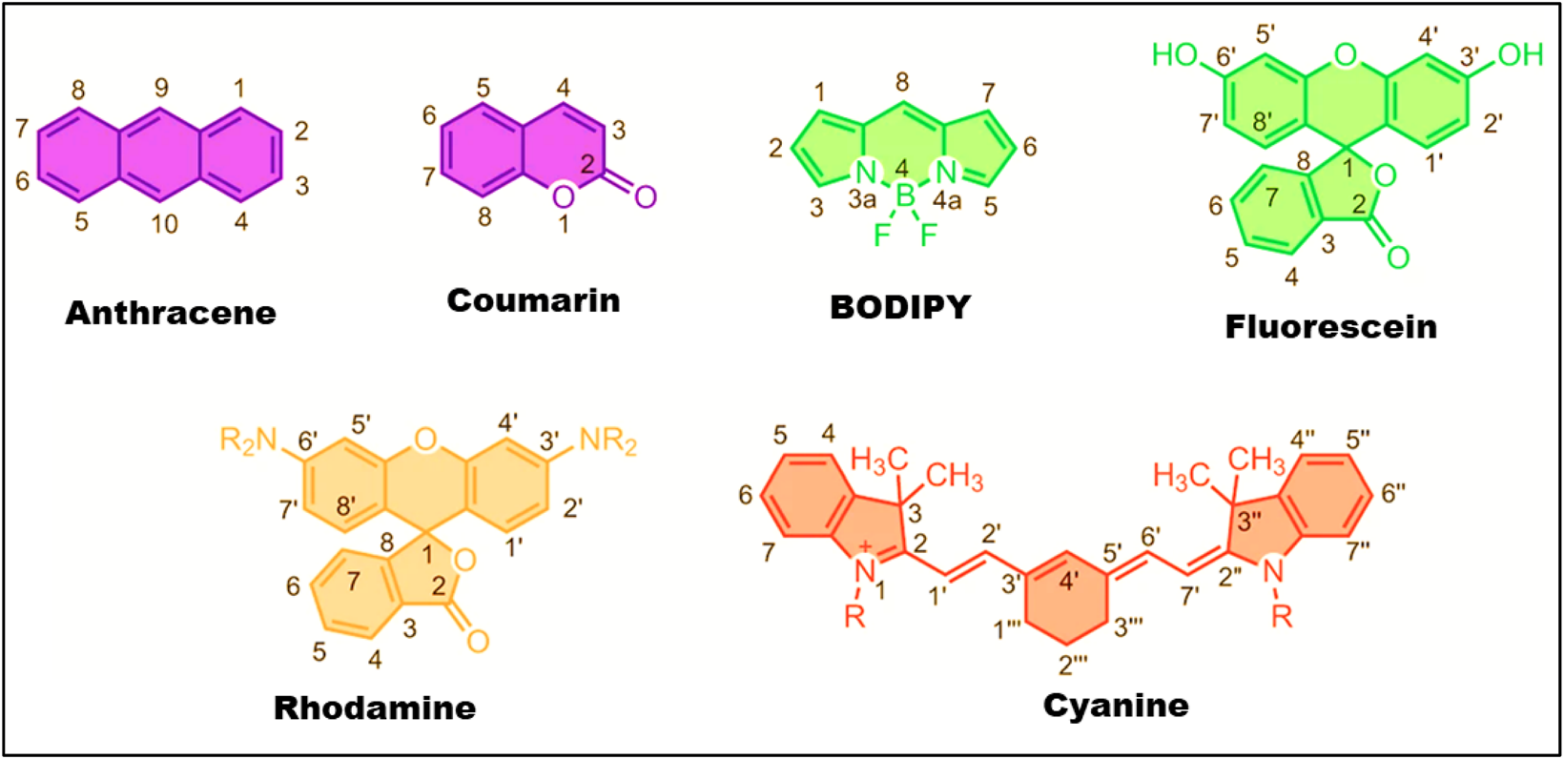
Common fluorescent probes for bioimaging. (Adaptedt with permission from ref. 26 Copyrights 2012, Nature chemistry).

In response to the growing demand for accurate, real-time, and high-resolution imaging in biomedical research and diagnostics, FLIM has emerged as a powerful alternative to conventional optical methods.^21,22^ Unlike traditional fluorescence microscopy, which depends heavily on signal intensity and is often affected by factors like fluorophore concentration and photobleaching, FLIM offers non-destructive, real-time imaging with high spatial resolution.^23^ It detects cellular and tissue-level biological structures, making it highly valuable in clinical practice. FLIM enables label-free metabolic imaging of autofluorescent molecules such as nicotinamide adenine dinucleotide (NADPH) and flavin adenine dinucleotide (FAD), and also facilitates the tracking of organelle-specific changes, intracellular trafficking, signaling pathways, and molecular interactions.^24^ The effectiveness of all imaging techniques, including FLIM depends on the performance of the probes,^25^ which are critical for image quality, resolution, and depth of detection (Fig. 2).

While the traditional fluorophores (*e.g.*, coumarins, BODIPYs, cyanines) have often suffered certain drawbacks that limit the imaging quality, brightness, Stokes shift, and tissue penetration.^27–29^ It also has poor photostability due to ROS generation during triplet–state transitions, which accelerate deterioration. To overcome those hurdles, the modern probes are engineered with specific improvements. Firstly, excitation/emission wavelengths higher than 650 nm diminish autofluorescence. Secondly, high brightness achieved by excellent extinction coefficients and quantum yields (QYs) allows for low-exposure imaging. Further enhancements contain significant Stokes shifts (>100 nm) to reduce self-quenching, improved solubility to prevent aggregation, and customized permeability for live-cell labeling.^30–32^ These alterations are significantly improving imaging performance and enabling researchers to better understand biological processes at both the molecular and cellular levels. As a result, FLIM is being integrated into clinical diagnostics, offering improved capabilities for pathogen detection, early cancer diagnosis, drug delivery, and metabolic imaging. Unlike structural imaging methods, FLIM uniquely captures nanosecond-scale decay kinetics of fluorophores, making it highly sensitive to pH, ion concentrations, and molecular interactions. FLIM has shown remarkable potential in early cancer detection through NADH lifetime analysis (92% accuracy in oral cancer screening) and Alzheimer's research *via* tau protein aggregation monitoring.^33,34^ Its compatibility with label-free metabolic imaging using NADH and FAD, along with advanced computational tools, has made it indispensable in both fundamental biology and clinical practice. This highlights the critical importance of sensitive and timely detection technologies in combating disease progression. To meet these needs, researchers are not only working on the imaging techniques but also pay high attention to the fluorescent materials.^35–37^ Among these, a wide variety of fluorescent probes/materials are being employed, including organic dyes, fluorescent proteins, quantum dots (QDs), up-conversion nanoparticles,^38^ silica nanoparticles, gold nanoparticles, and fluorescent metal nanoclusters.^39^ Each offers unique advantages in imaging stability, brightness, biocompatibility, and functional tunability. Notably, carbon-based nanomaterials (CNMs) have gained significant interest due to their desirable properties, such as earth abundance, low cost, and resistance to photobleaching.^40–42^ These characteristics make CNMs highly promising for applications in biosensing, bioimaging, solar cells, phototherapy, drug delivery, and batteries.

**Fig. 3:**
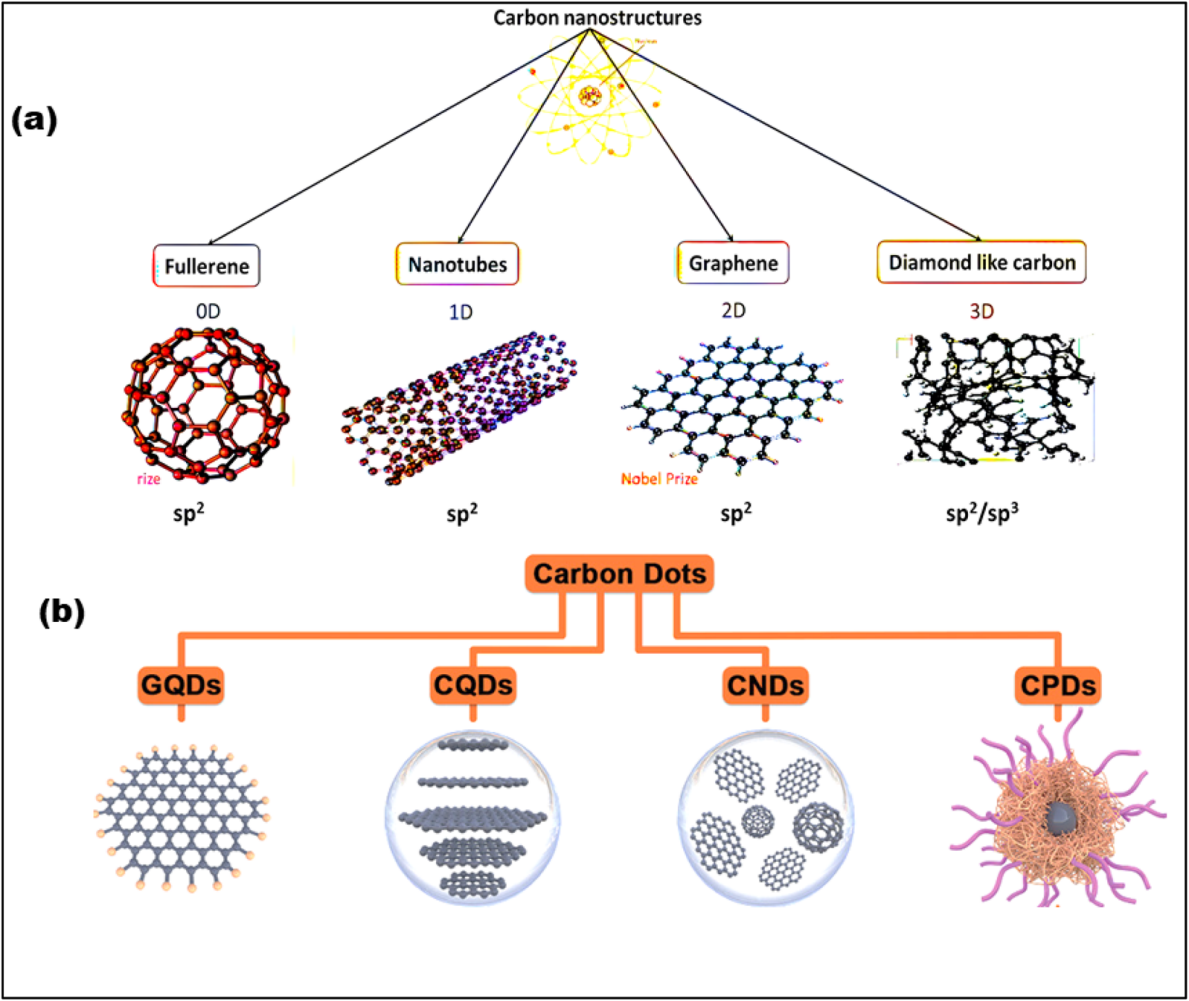
(a) Graphical representation of carbon family that varies from 0D to 3D (adapted with permission from ref. 55 Copyrights 2021, Materials, MDPl). (b) Four categories of CDs and their structures: graphene quantum dots (GQDs), carbon quantum dots (CQDs), carbon nanodots (CNDs), and carbonized polymer dot (CPDs) (Adapted with permission from ref. 56 Copyright 2021, Wiley-VCH).

The family of CNMs comes in a variety of forms, spanning from 0D to 3D structures, and can exist in both crystalline and amorphous states^43,44^ (Fig. 3a). The remarkable sp^2^ hybridization of carbon is one of its most striking properties, which allows it to generate a wide range of nanostructures.^45,46^ The members of this multipurpose family all have their own set of unique properties; for example, diamond is very hard and very thermally conductive, and graphene is very electrically and mechanically strong. Therefore, the researchers engaged in the advancement of fluorescent and luminescent CNMs, including silicon nanoparticles, polymer dots (PDs), upconversion nanoparticles, and CDs. Regardless of these developments, CDs have risen to recognition as a biocompatible, environmentally responsible, and affordable fluorescent CNM.^47^ It's worth mentioning that, researcher made a serendipitous finding during the purification of carbon nanotubes (CNTs) when they observed a luminous fraction of CNMs, leading to the identification of CDs.^48^ Afterward, atomic force microscopy (AFM) was used to analyze the fraction, sparking considerable interest among researchers thereafter. CDs are characterized by their diminutive size (<10 nm), graphitic core, and surface functional groups such as hydroxyl (–OH), amino (–NH_2_), and carboxyl (COOH).^49^ These characteristics endow CDs with exceptional fluorescence, biocompatibility, aqueous solubility, and adaptability for diverse applications.^50,51^ With adjustable emission spanning from ultraviolet (UV) to NIR, exceptional photostability, and resilience against photobleaching. In general, CDs are divided into four categories based on their core structure. These include graphene quantum dots (GQDs) with a 2D graphene core, carbon quantum dots (CQDs) with a spherical crystalline core, carbon nanodots (CNDs) with an amorphous core, and carbonized polymer dots (CPDs), which have a polymer matrix that is either dehydrated or partially graphitized^52–54^ (Fig. 3b).

**Fig. 4:**
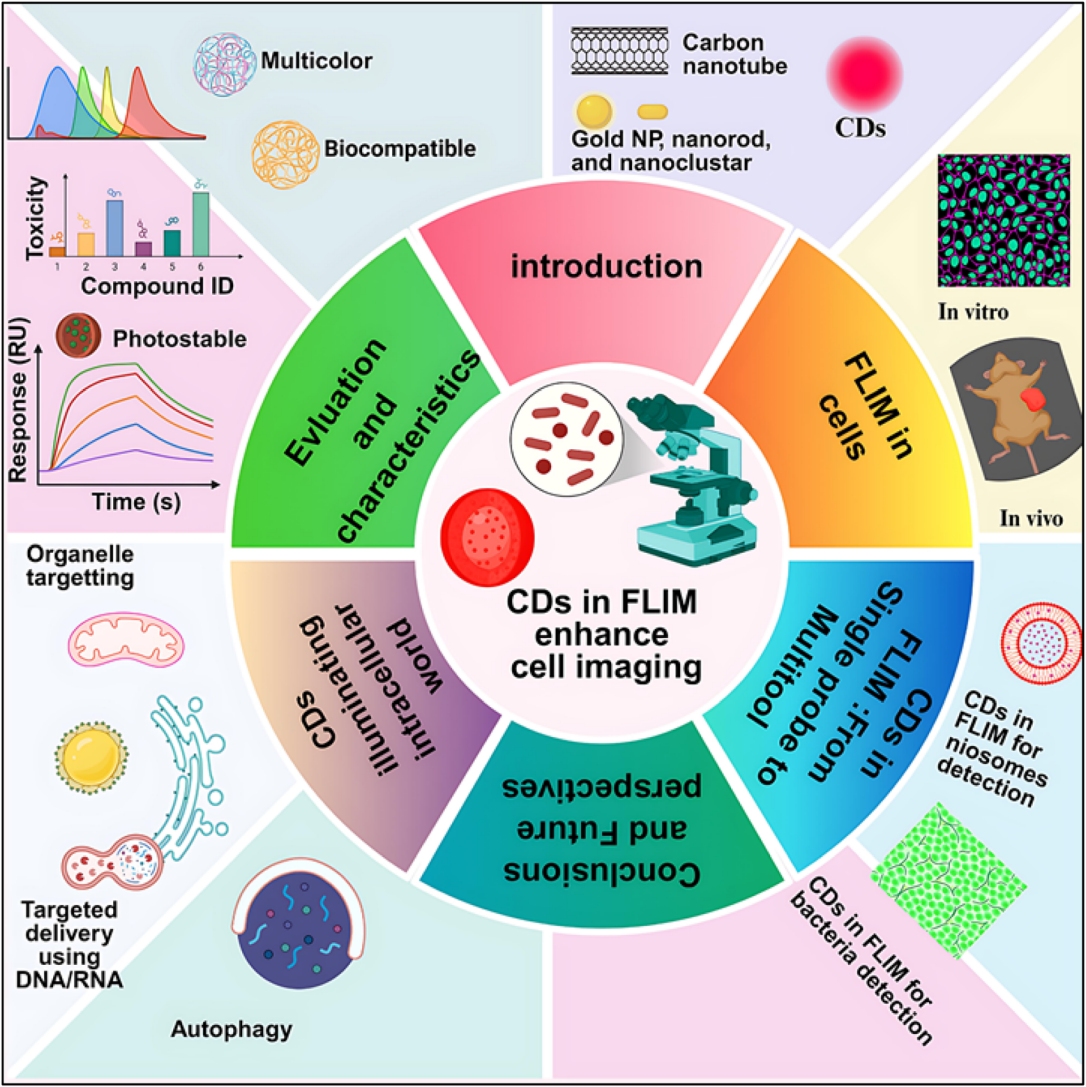
The pictorial representation of the paper.

CDs are synthesized through diverse strategies, broadly classified into top-down and bottom-up approaches. Top-down methods include arc discharge, laser ablation, electrochemical oxidation, and chemical oxidation. Whereas bottom-up techniques involve hydrothermal/solvothermal processes microwave-assisted synthesis, pyrolysis and template-assisted methods. Due to their heteroatom doping, surface passivation, and quantum confinement, CDs are more optically versatile than organic dyes and classic fluorophores like QDs, and they don't contain the harmful heavy metals that QDs normally contain.^57^ The synergy between FLIM and nanomaterials directly addresses the earlier-mentioned clinical need for sensitive detection of cellular abnormalities, while the photostability of CNMs specifically mitigates FLIM's photobleaching challenges in thick tissues. Together, these developments in FLIM-coupled nanomaterial systems represent significant progress toward achieving the required diagnostic sensitivity and therapeutic efficacy to combat disease progression and recurrence. FLIM is considerably enhanced by CDs because they enable particular targeting, multiplexing, and valuable lifespan information.^58,59^ Their remarkable adaptability renders them a highly promising tool for unraveling the mysteries of cells. A number of studies have been published covering the synthesis, characteristics, and uses of CDs in many different potential sectors. To the best of our knowledge, no comprehensive review has yet focused on the application of CDs in cellular imaging utilizing FLIM. Therefore, this review provides an integrated perspective combining the photophysical understanding of CDs with advanced FLIM methodologies to elucidate cellular and subcellular processes. The novelty of this work lies in presenting, for the first time, a systematic analysis of CDs as FLIM probes for cellular imaging, bridging the gap between nanomaterial photophysics and fluorescence lifetime-based bioimaging. Thus, this review begins by exploring the photophysics of CDs, delving into their luminescence mechanisms and emphasizing the process of cellular uptake and the distribution of CDs within intracellular organelles. The discussion then transits to provide a concise overview of FLIM instrumentation, covering both time-domain (TD) and frequency-domain (FD) techniques. It also explores the integration of FLIM with targeted dyes and nanoparticles, highlighting their applications in revealing cellular dynamics. The unique capability of FLIM to capture fluorescence lifetime (*τ*) data is emphasized, showcasing its potential to study the temporal changes in cellular behavior. Finally, the review examines several biological applications of FLIM in combination with CDs, showcasing their transformative role in cellular and subcellular imaging. The graphical abstract of the entire paper is shown in (Fig. 4).

## 2. Evaluation and characteristics of CDs

**Fig. 5:**
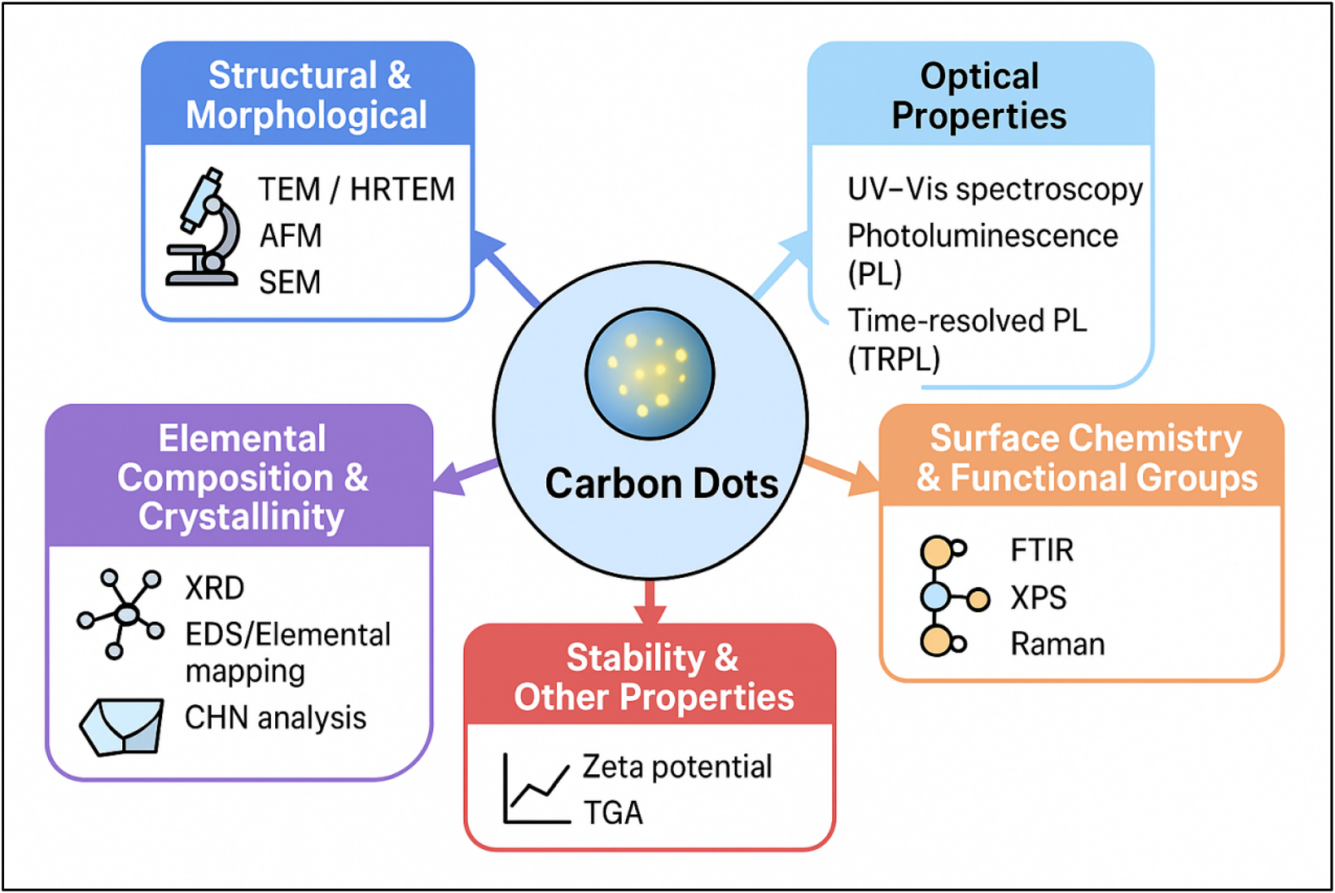
Overview of characterization methods for CDs.

Understanding the structural and optical properties of carbon dots (CDs) is crucial for their wide-ranging applications. Techniques such as Transmission Electron Microscopy (TEM) allow for ultrastructural identification, while Fourier Transform Infrared Spectroscopy (FTIR) is employed to analyze oxygen-containing functional groups. Additionally, efficient UV absorption, unique PL characteristics, and insights into lattice structure through X-ray diffraction (XRD) all contribute to a comprehensive understanding of CDs.^60^ AFM is another powerful technique that provides imaging at nanometer-scale resolution. In AFM, a cantilever with a sharp tip scans the surface of the sample.^61^Table 1 and (Fig. 5) represent valuable insights into the characterization of CDs, enhancing our understanding of their properties through these key techniques.

**Table 1:** Characterization techniques for CDs

Method	Objective/key aspect	Key insights	References
TEM	Ultrastructure identification; high resolution (0.1–0.2 nm)	Provides information on morphology, size (<10 nm), and dispersion	62
FTIR	Analysis of oxygen-containing groups; key peaks: O–H vibrations, C=O bonds	Reveals surface modifications and passivation effectiveness	63
UV-absorbtion	Efficient UV absorption (π–π* transitions of C=C bonds); (n–π* transition of C=O and C=N bonds)	Generally higher efficiency than graphene quantum dots (GQDs) in absorbing longer wavelengths; distinct peak in the 270–390 nm range	64
PL	Distinctive feature: PL influenced by quantum confinement; enhancement through surface passivation	Emission peaks correspond to specific wavelengths	65
XRD	Characterization of particle size, phase purity, and crystal structure	Specific patterns provide insights into structural characteristics	66
Nuclear magnetic resonance (NMR)	Insights into hybrid carbon atoms and carbon atom interactions	Identifies distinctive peaks linked to the chemical structure, aiding in the determination of structure and composition	67
Raman spectroscopy	Characterizing lattice structure and properties; distinctive feature: CDs exhibit distinct Raman bands	Provides insights into size and structural characteristics	68
AFM	Observes individual dots to determine size and shape; investigates aggregation and interactions with other materials	Offers valuable insight into morphology, size distribution, and surface characteristics of nanoscale objects	69

The optical properties of CDs make them highly valuable for a range of applications, particularly in cellular imaging. These properties stem from the unique surface chemistry and electronic structure of CDs, which set them apart from other nanomaterials. CDs are known for their impressive PL, high QY, broad emission wavelength range, and outstanding stability, all of which contribute to their usefulness in various optical applications.^70,71^ Despite their small size, typically ranging from a few nanometers to tens of nanometers, CDs possess a high Specific Absorption Value (SAV) ratio. This high surface area allows them to interact effectively with target cells, which is beneficial for cellular imaging and drug delivery applications. However, this large surface area can also limit their ability to load drugs, which is an important consideration in designing CD-based drug delivery systems. The optical and chemical characteristics of CDs are primarily determined by their synthesis process and precursor materials.^72^ As noted in several studies, these factors such as the choice of precursors, reaction conditions, and the method of synthesis directly influence the final properties of CDs.^73,74^ Therefore, understanding these factors is essential for tailoring CDs to specific applications. The main characteristics of CDs that make them perfect for cellular imaging applications will be the subject of this section.

### 2.1 Fluorescence

The capability of CDs to emit light at longer wavelengths and absorb light at shorter wavelengths is known as their fluorescence. In particular, by modifying their chemical makeup, particle size, and adding surface flaws, these carbon nanoparticles undergo changeable fluorescence ability. The size of a GQDs is less than 100 nm, and it is a multifaceted, 2D sheet that is bonded with sp^2^-carbon.^75^ Graphene, graphene oxide (GO), or aromatic molecule condensation is the typical material for them.^76^ The presence of polyaromatic structures and other functional moieties in graphene leads to a quantum confinement phenomenon, that results in intense fluorescence emitted by GQDs.^77^ CNMs with remarkable chemical, mechanical, and optical characteristics are being created by the use of fluorescent nanodiamonds.^78^ The building blocks of nanodiamond and nanocrystals are carbon atoms connected in a tetrahedral pattern, which form a cubic lattice in three dimensions, and a carbon shell like an onion, which contains functional groups on its surface.^79^ Atomic defects within nanodiamonds are the source of their fluorescence. Absorption and emission bands can be adjusted owing to the two primary kinds of nitrogen–vacancy (N–V) defect centers, respectively.^80^ Because of its high fluorescence QY and exceptional photostability, nanodiamond enables prolonged imaging without the possibility of signal deterioration.^81^ Enhancing imaging time without worrying about signal loss is possible with nanodiamond because of their very photostable nature and strong fluorescence QY. They are safe for N–V centers to surface functionalize since they are both biocompatible and cytotoxic,^82^ because of these advantages, fluorescent nanodiamonds are used for tissue engineering,^83^*in vivo* imaging of animals, drug/gene delivery, subcellular biomarkers, and other similar applications.

The complex area of optical imaging has recently caused researchers to give an enormous amount of energy and time to investigate ways to utilize CDs' luminescence properties.^84^ However, most of them allow blue light instead of photons with longer wavelengths, which range from green to red.^85^ A ʻbiological window’ in the NIR spectrum has been observed in fluorescence emission by some recently described CDs'. Enhancing the signal-to-noise ratio (SNR) is achievable by confining organismal autofluorescence to the NIR spectrum. Future advances in bioimaging could be significantly enhanced by developing multi-color, long-wavelength-emitting CDs that are water-soluble, photostable, and resistant to interference. Using citric acid (CA) as a substrate, Pan. *et al.* successfully synthesized full-spectrum CDs. By adjusting excitation wavelengths from 330 nm to 600 nm, these full-color CDs maintained consistent emission intensity across nearly the entire visible spectrum.^86^ In a another study, Liu *et al.*proposed a method to create three types of CDs-*m*-CDs, *o*-CDs, and *p*-CDs by microwave heating *o*-phenylenediamine (*o-*PD), *m*-phenylenediamine (*m*PD), and *p*-phenylenediamine (pPD). When exposed to a 365 nm UV laser, these CDs emitted blue, yellow and orange fluorescence, respectively.^87^

In another study polyethyleneimine (PEI) and 1, 2, 4-triaminobenzene were used by Li, *et al.*^88^ to produce modified CDs that show minimal toxicity and intercellular red fluorescence. Nanoscale complexes can be formed by these CD's bonding interactions with small interfering RNA (siRNA). The resulting imaging-trackable complex can be utilized to treat glioblastomas (GBM) and transfer growth factors collected from hepatomas to human glioma cells.

**Fig. 6:**
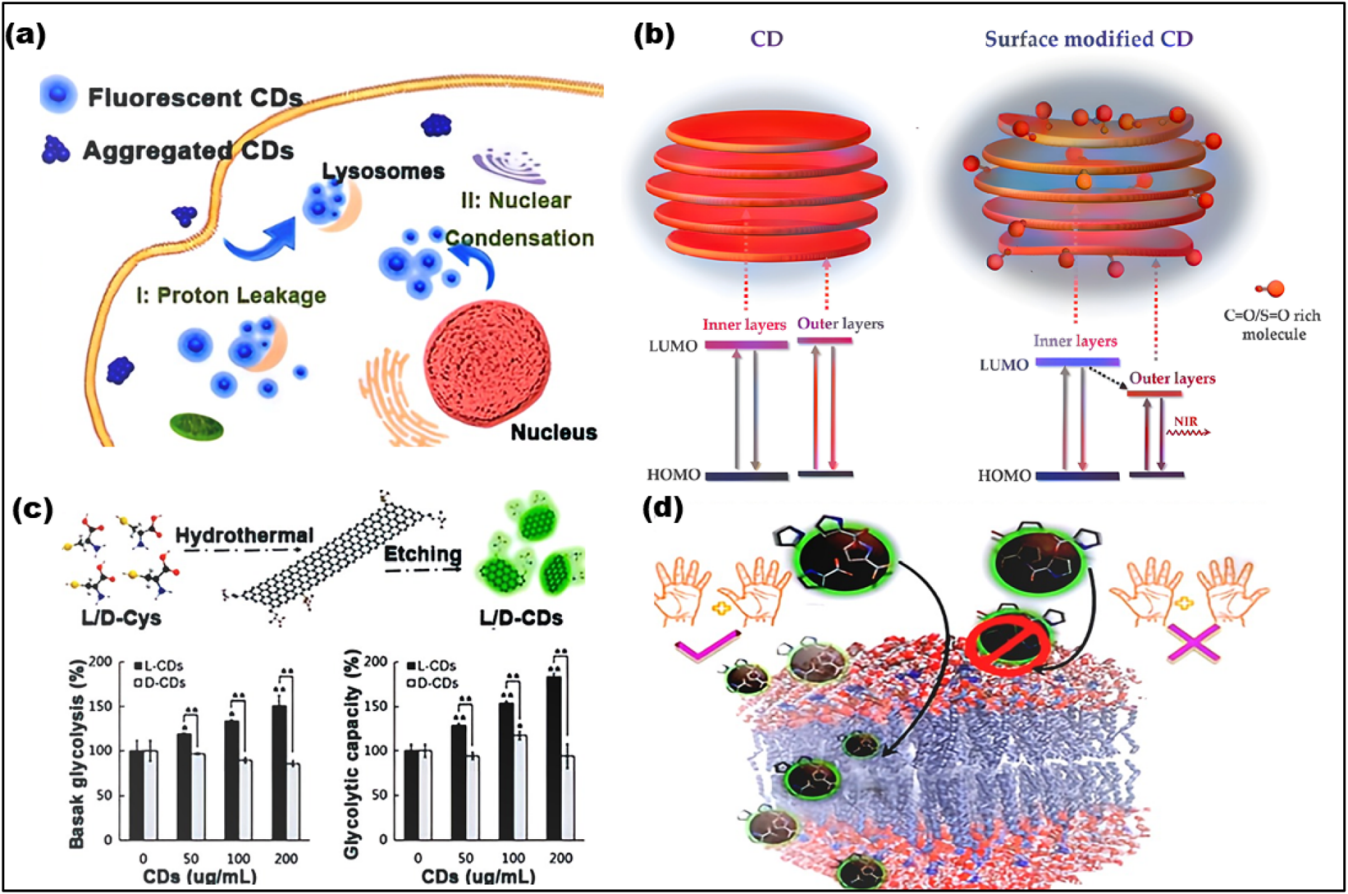
Optical properties of CDs. (a) High QY CDs for visualization of lysosomes. (Adapted with permission from ref. 89 Copyright 2020, The Authors, published by Elsevier). (b) NIR-emitting CDs for bio-imaging. (Adapted with permission form ref. 90. Copyright 2018, Wiley-VCH). (c) Chiral CDs for the treatment of T24 cells. (Adapted with permission from ref. 92 Copyright 2018, Wiley-VCH). (d) Chiral CDs demonstrating high selectivity for cell membranes. (Adapted with permission from ref. 93 Copyright 2018, American Chemical Society).

Optimizing CDs for various applications thus involves careful precursor selection, specific targeting moieties, and methods for surface modification and passivation. The optical characteristics of CDs, such as their red and NIR emissions, up-conversion photoluminescence (UCPL), high fluorescence quantum yield (FQYs), and promise for NIR-based applications (Fig. 6a),^89^ are drawing growing interest. In healthcare, CDs have demonstrated potential, especially in chiral luminescence and photothermal treatment. However, since most CDs absorb in the UV region and produce blue-green light, the limited penetration of short-wavelength light in biological tissues makes detection difficult. The use of blue-green emission CDs with high FQYs can help to mitigate this problem. Furthermore, CDs can be passively seen in living cell lysosomes due to their acidophilic nature. For example, branched polyethyleneimine (bPEI) and rose bengal (RB) were used as precursors to create green-emitting CDs with a FQY of 90.49%. Red and NIR-emitting CDs are of great interest due to their reduced tissue absorption, improved tissue penetration, and low autofluorescence interference (Fig. 6b).^90^ The potential of red/NIR-emitting CDs for labelling applications in *in vitro* cell imaging has been evaluated using a variety of cancer cell lines. The data indicates that red/NIR-emitting CDs primarily aggregate in the cytoplasm and on the cell membrane.^91^

*o*-PD^94^ was used as the precursor to create remarkable red-emitting CDs with high FQYs. These CDs were then used as fluorescent probes for both *in vitro* and *in vivo* imaging applications. Furthermore, a variety of red-emissive CDs made from o-PD have been created for use in bioimaging. The produced CDs can be utilized to study the dynamic changes of the Golgi apparatus during the early phases of viral infection, and because of their high light stability and biocompatibility, they are well suited for long-term *in situ* imaging of the Golgi apparatus, (Fig. 6c). In addition, chiral cysteine has been frequently employed as a stabilize and chiral ligand to alter the characteristics of nanomaterials. When human bladder cancer T24 cells were exposed to chiral CDs made with (l or d)-cysteine, l-CDs caused an increase in glycolysis while d-CDs had no such impact (Fig. 6c).^92^ For example, d-proline preferentially interacts with the cell membrane or liposome mimics due to its inverted chirality (Fig. 6d).^93^

### 2.2 UV-vis absorption and band gap

The bandgap and UV-vis absorption spectrum, play a major role in determining how CDs interact with light. In the UV-vis absorption spectrum, CDs typically display broad absorption bands that span both the UV and visible regions.^95^ The specific position and shape of these bands are influenced by various factors, including the size of the CDs, their surface chemistry, and the degree of conjugation in their carbon core. Larger CDs generally exhibit absorption across the UV-vis range, while smaller CDs tend to absorb primarily in the UV region, typically showing higher energy absorption.^96^ Importantly, the optical properties of CDs can be modified through surface functionalization. By incorporating different functional groups onto the surface, new energy levels can be introduced, and the absorption spectrum can be adjusted. This tunability allows researchers to fine-tune CDs absorption properties for specific applications, such as bioimaging, sensing, and optoelectronics.^72^ For example Yan *et al.*^97^ produced CDs emitting colors from blue to red, with fluorescence spectra spanning 450–650 nm, by applying a heat treatment technique derived from phenol. They further investigated the capability of the synthesized yellow and green CDs for non-metal detection. Their study demonstrated that these CDs responded swiftly and accurately to chlorine monoxide, with detection limits of 31.1 nM (nanomolar) and 56.5 nM within the concentration ranges of 0–50 µM (micromolar) and 4–44 µM, respectively. CDs with narrower bandgaps tend to emit NIR light, while those with larger bandgaps emit blue or UV light. The bandgap can be tuned by altering the size of the CDs, as quantum confinement effect (QCE) causes the bandgap to widen in smaller CDs. Additionally, doping CDs with heteroatoms like nitrogen (N), phosphorus (P), or sulfur (S) can further modify the bandgap, shifting the emission wavelengths into the visible or NIR range.^98^ Surface passivation and functionalization processes also affect the bandgap by changing the energy levels and electrical transitions within the CDs.S.

### 2.3 PL

**Fig. 7:**
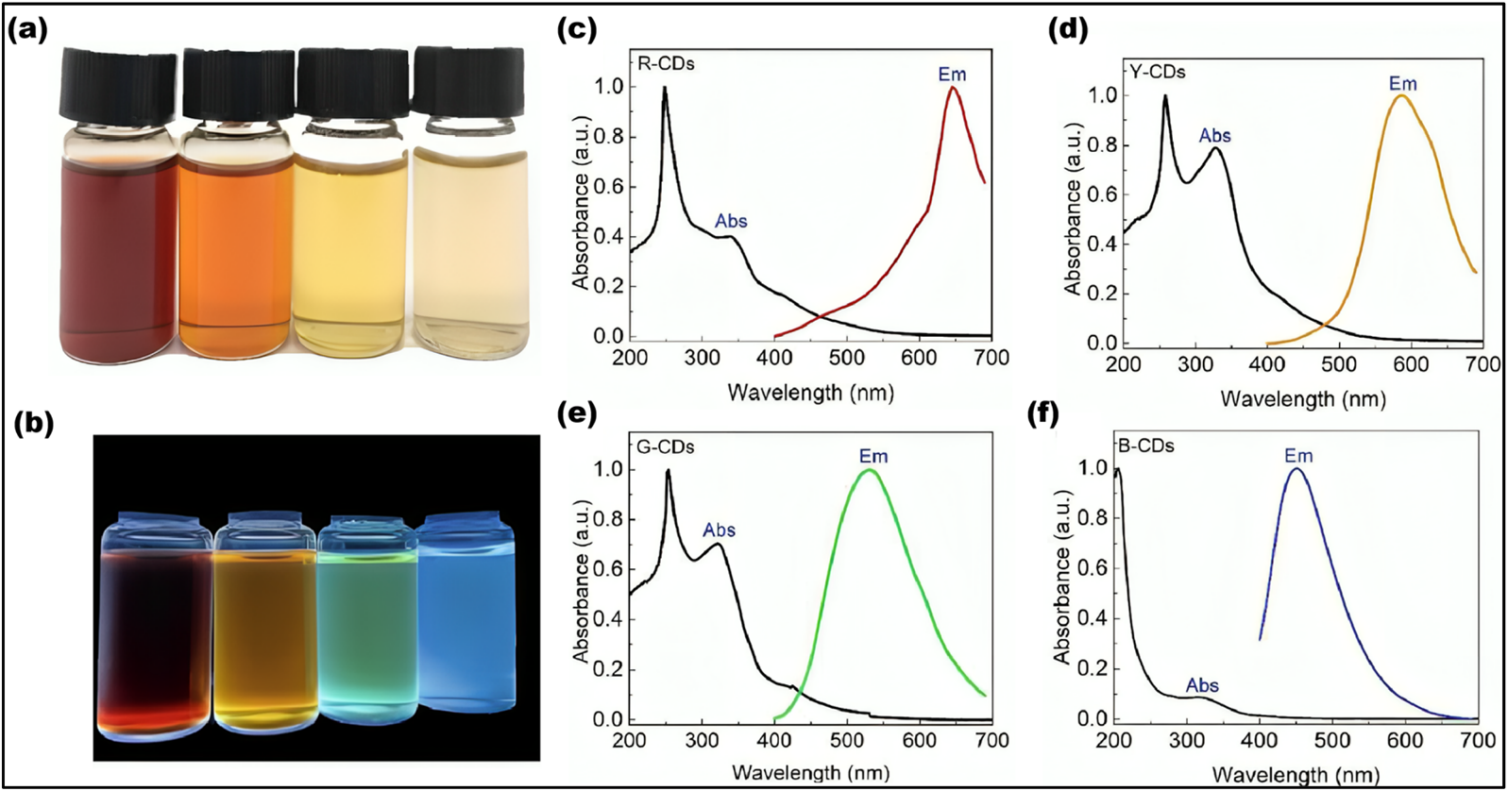
B-CDs, G-CDs, R-CDs and Y-CDs under (a) sunlight and (b) UV irradiation (365 nm). (c) R-CDs’ and (d) Y-CDs’ emission and UV-adsorption spectra respectively. (e) UV-adsorption and PL emissions of G- CDs. (f) The emission, UV-adsorption related to B-CDs. (Adapted with permission from ref. 100 Copyright 2022, MDPI (open access)).

The PL properties of CDs are influenced by several factors, including size, surface functionalization, chemical composition, and structural defects. PL refers to the ability of CDs to emit light when excited, with emission wavelengths ranging from UV to NIR.^99^ One key method for tuning the PL of CDs is by adjusting their size. Surface functionalization is another crucial approach, where different functional groups or chemicals are introduced to modify the electronic transitions and energy levels of the CDs. This adjustment can help customize their emission characteristics. The PL of CDs can be further explained by three primary mechanisms: fluorescence of molecules, core state emission, and surface state emission. Fluorescence emission can originate from the molecules or precursors present during the synthesis, where carbonization of small molecules creates organic fluorophores that serve as the centers for fluorescence. Core state emission, on the other hand, arises from specific functional groups or crystal defects within the CDs. Surface state emission involves a red shift in emission wavelengths caused by surface defects, such as increased oxidation, leading to fluorescence due to coordination bonds between carbon and adjacent chemical groups. PL properties are also highly sensitive to the excitation wavelength (*λ*_ex_) and the intensity of the emitted light. By modifying the chemical structure of CDs, their PL characteristics can be fine-tuned for specific applications, such as bio-imaging or sensing. Researchers can manipulate the CD structure or facilitate energy or electron transfer processes to control their emission characteristics.^96^ Rao *et al.*^100^ and their team demonstrated that the PL emission wavelengths of the CDs could shift from 451 nm to 654 nm by adjusting the volume ratio of water to Dimethylformamide (DMF). PL emission and UV-vis absorption spectroscopy were used to determine the optical properties of these CDs, and four different PL emission peaks were observed: red-emitting (R-CDs) at 645 nm, yellow-emitting (Y-CDs) at 586 nm, green-emitting (G-CDs) at 530 nm, and blue-emitting (B-CDs) at 451 nm as shown in Fig. 7.^100^

Among the different processes contributing to PL, surface state and core state emissions play particularly significant roles in determining the overall PL behavior. Core state PL, which arises from nanoscale carbon cores and polymer clusters, can be adjusted to enhance luminescence characteristics, improving selectivity and sensitivity in applications like ion detection.

### 2.4 Luminescence mechanisms of multifunctional CDs (MCDs)

The adjustable emission of light from CDs is an exciting property. A complete comprehension of their luminescence mechanisms is required to produce CDs with changeable emission qualities. Surface states, QCE, and the degree of graphitization are the three main mechanisms that allow tunable fluorescence in CDs. This section will analyze how these elements lead to the multicolor emission displayed by MCDs.

#### 2.4.1 Surface state

The MCDs are rather sensitive to the surface states, especially to the surface defects and functional groups. Fluorescence emission can occur due to defects on the surface that are associated with surface oxidation and can serve as regions that trap excitons. These defects produce several emission areas dependent on the oxidation level; the higher oxidation increases emission efficiency.^101^ Emission domains particular to chemical groups attached to the surface are –COOH, –OH, carbonyl (–C=O), and –NH_2_ group. Thus, the several electronic states generated by these functional groups lead to variations in the MCD emission characteristics whereby the emission spectrum shifts and the intensity varies.^102^

**Fig. 8:**
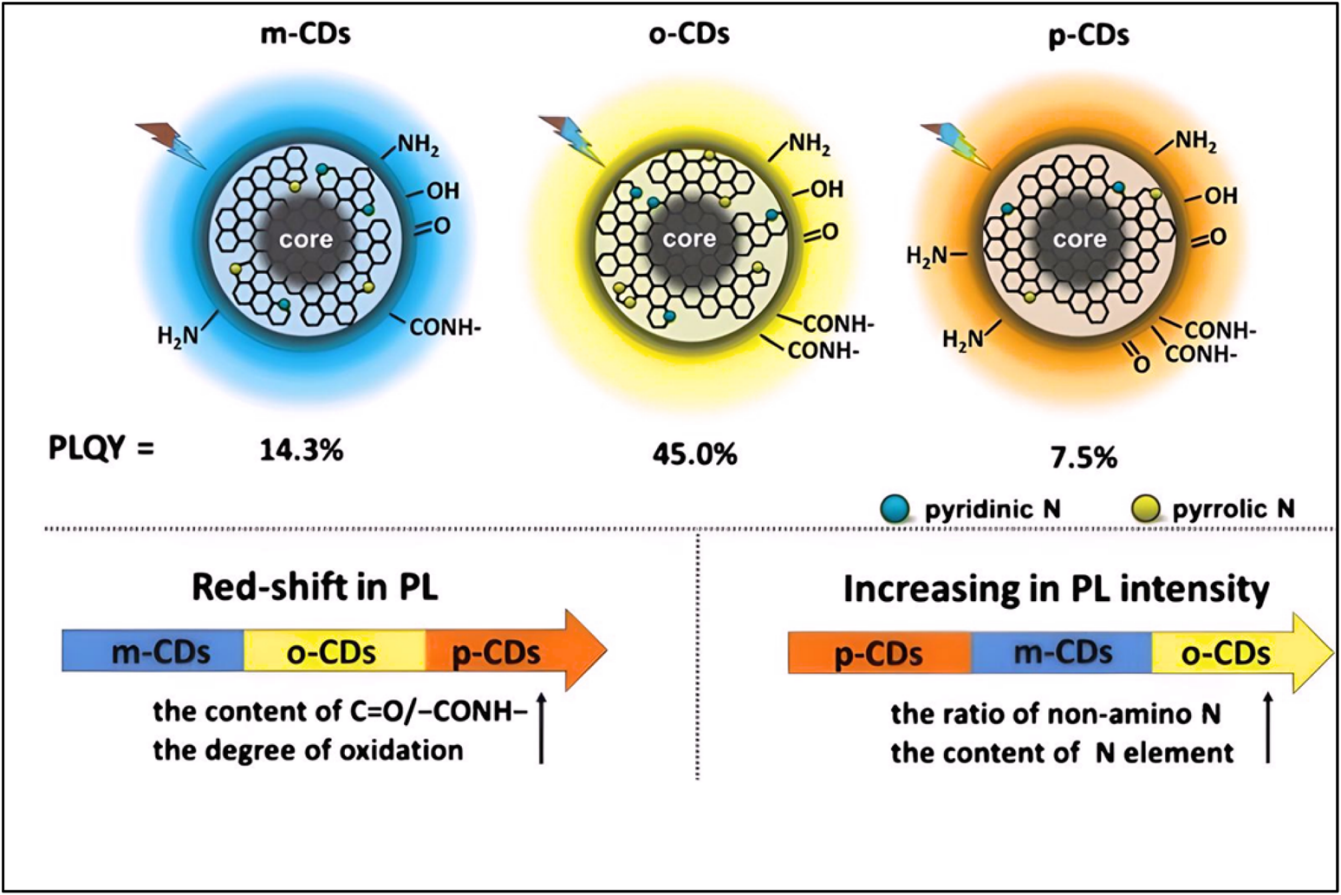
The mechanism diagram of polychromatic CDs. (Adapted with permission from ref. 87 Copyright 2018 Springer).

Through changing different reaction conditions including synthetic method, solvent, temperature, as well as the type and ratio of reactants, it is possible to synthesize MCDs through distinct outward properties which consequently affect the release of light. Therefore, the surface states have the potential to be modified in terms of the quantity and kind of purposeful groups^103,104^, hydrogen bonding,^105–107^ or by altering the content of elements including N, O_2_, S, or P on the CDs. One example is MCDs developed by Jia *et al.* where orange, green, and blue-MCD lights were produced. They noted that with the increase in the change from blue to orange, the number of pyrrolic nitrogen and –C=O group increased as well. This implies that there was a higher extent of oxidation and the density of N-related surface state was higher in the orange MCDs. Such imperfections on the surface work as the spots where excitons are trapped, implying a reduction in energy disparity and a shift in the emitted light towards longer wavelengths.^108^ Similarly, Xu *et al.* discovered that the fluorescence secretion in MCDs changed from green to red as the C=N group ratio increased from 35.6% to 58.4%. Since the C=N group is a solid electron-acceptor, more of these in the molecules result in push–pull processes that generate internal charge transfer (ICT) and, eventually, a change in color.^109^ Liu *et al.* pointed out that the incorporation of (–C=O) and amide (–CONH) returned a shift towards the longer wavelength of emitted light. On the other hand, an increase in the content of non-amino N improved the brightness of the fluorescence (Fig. 8).^87^ Moreover, it is discovered that the choice of solvents in MCD synthesis can affect their luminous characteristics. The polarity of solvent was changed by scientist from DMF, ethanol, water, and acetic acid. The solvent hydrogen bonding with MCDs caused changes in the surface energy state and longer wavelength shifts of the emitted light.^106^ Recently, Song *et al.* held a study to investigate the effects of oxidation on CDs fluorescence. From the analyzed results, it was determined that increasing the amount of nitric acid (HNO_3_) in the reaction process raised the oxidation level of MCDs. By increasing the number of oxygen atoms on the surface structure, this modification caused the energy gap between the valence and conduction bands to increase, which in turn caused the light that was released to shift toward longer wavelengths. This redshift mechanism was further confirmed through the use of theoretical calculations.^110^

#### 2.4.2 QCE

**Fig. 9:**
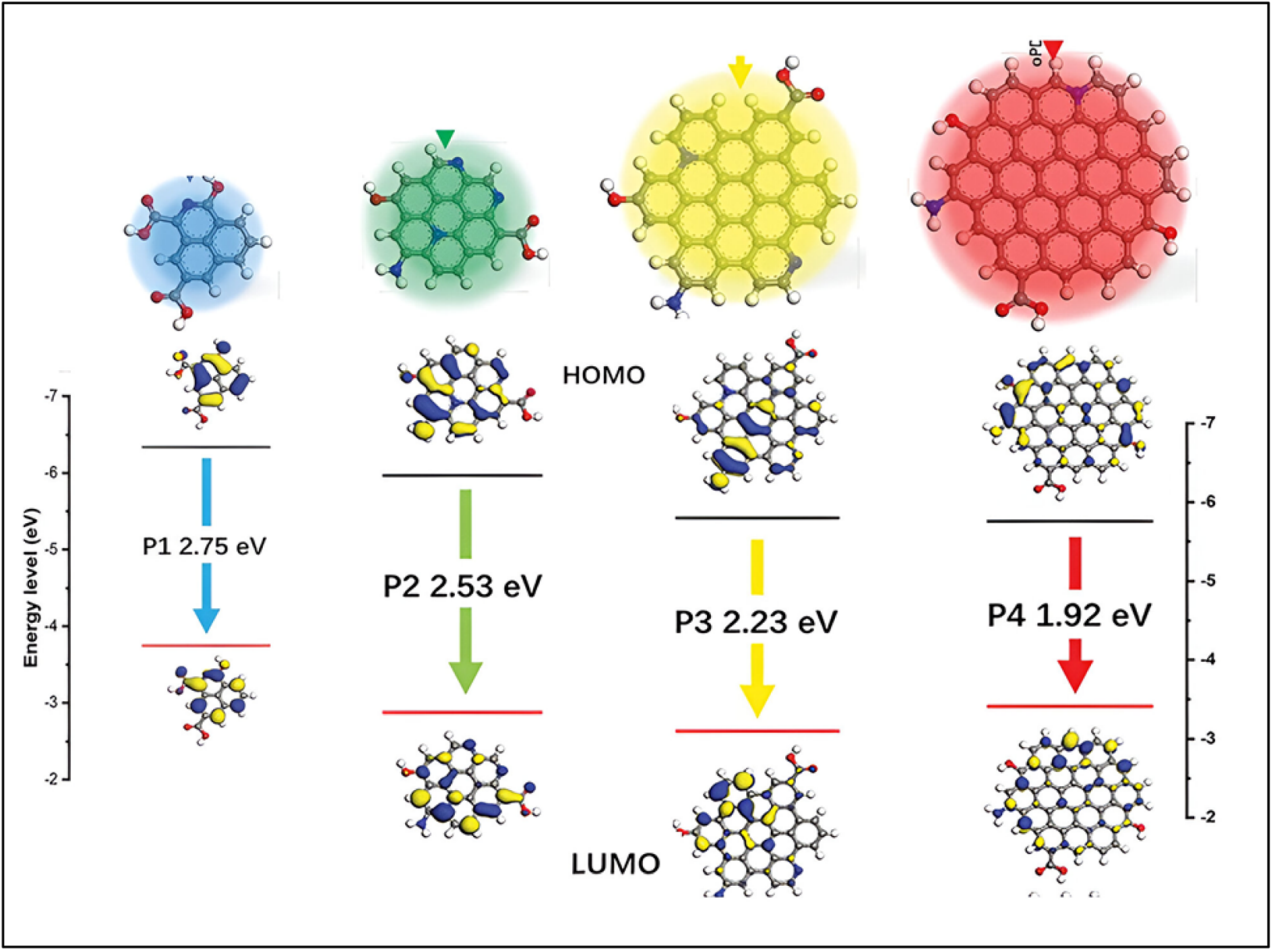
Mechanism of tunable fluorescence for CDs by adjusting QCE. (Adapted with permission from ref. 114 Copyright 2022 Wiley-VCH).

Several studies have explored how the fluorescence emission characteristics of MCDs relate to particle size.^111^ For example, Wu *et al.* showed that MCD emission could be controlled within a certain wavelength spectrum, from 513 to 612 nm, by varying particle size within the 2–8 nm range.^112^ It was also demonstrated by Ding *et al.* that MCDs are capable of exhibiting fluorescence from the blue to NIR spectrum. Hence, the density and concentration of sp^2^ coupled domains, and graphitic nitrogen in the structure improved with the increase in sample size.^113^ In addition, Cao *et al.* suggested that particle size and the percentage of graphitic nitrogen affect fluorescence in MCDs. Fig. 9 illustrates how the size of MCDs causes the amount of graphitic nitrogen to rise, which in turn causes the bandgap to shrink and the emission wavelength to shift toward the red region.^114^

#### 2.4.3 Graphitization

**Fig. 10:**
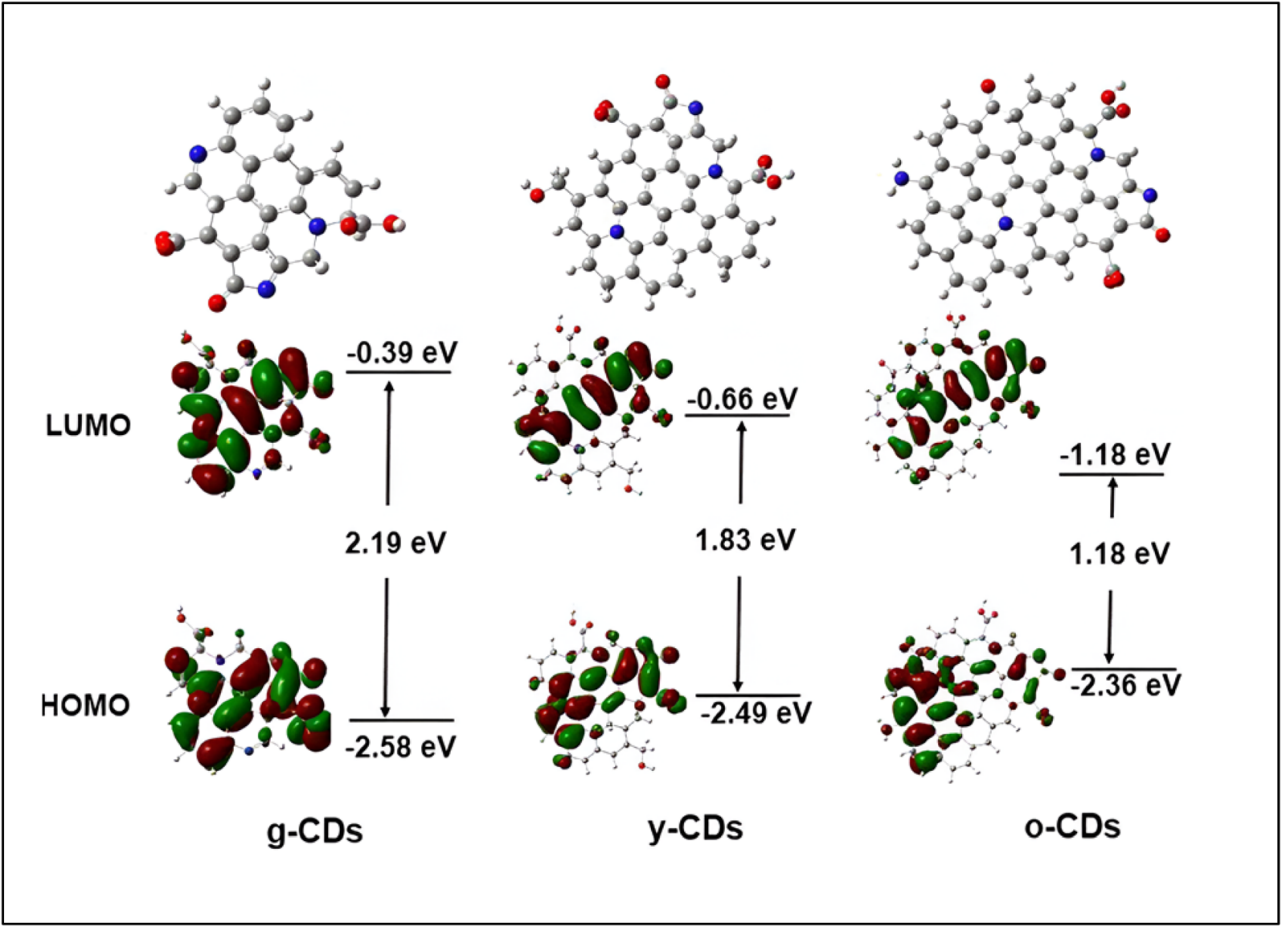
Mechanism of tunable fluorescence by adjusting graphitization degree. (Adapted with permission from ref. 115 Copyright 2021 Wiley-VCH).

It is possible to change the size of sp^2^/sp hybridized carbon cores in the middle of the MCDs, which will affect the degree of graphitization. It is theoretically suggested that more sp^2^ conjugated domains lead to the longer wavelength shift of the emission wavelength of MCDs.^115^ This indicates that graphitization holds a strong impact on the fluorescence properties of MCDs with an improved graphitization leading to a shift to the red end of the spectrum. Consequently, Sun *et al.* suggested that changing the solvents during synthesis can influence the amount of sp^2^ conjugated domains and lead to the red-shift in emitted wavelength.^116^ Wang *et al.* also used silica salve stake chromatography to purify white light-emitting CDs and obtained R, G, and B light-emitting CDs. Simulations using density functional theory (DFT) further showed that the expansion of sp^2^ conjugated domains and the presence of C=O functional groups are responsible for the redshift in emission wavelengths.^117^ A semi-analytical model was presented by Wang *et al.* to examine the optical characteristics of MCD cores (Fig. 10).^115^ According to this model, bandgap energy decreases (from 2.84 eV to 1.96 eV) when internal sp^2^/sp^3^ hybridization increases (from Nsp^2^ = 0.16 to 0.26 to 0.26), suggesting that a smaller bandgap is associated with a redshift in emission wavelength.^118^ Furthermore, Wang *et al.* verified that the peak emission wavelength steadily changed from 445 nm to 643 nm when the degree of graphitization and the size of sp^2^ domains in MCDs grew.^119^ The observed redshift is a result of the decrease in the bandgap energy that is caused because of an increase in the extent of graphitization.

### 2.5 Photostability

**Fig. 11:**
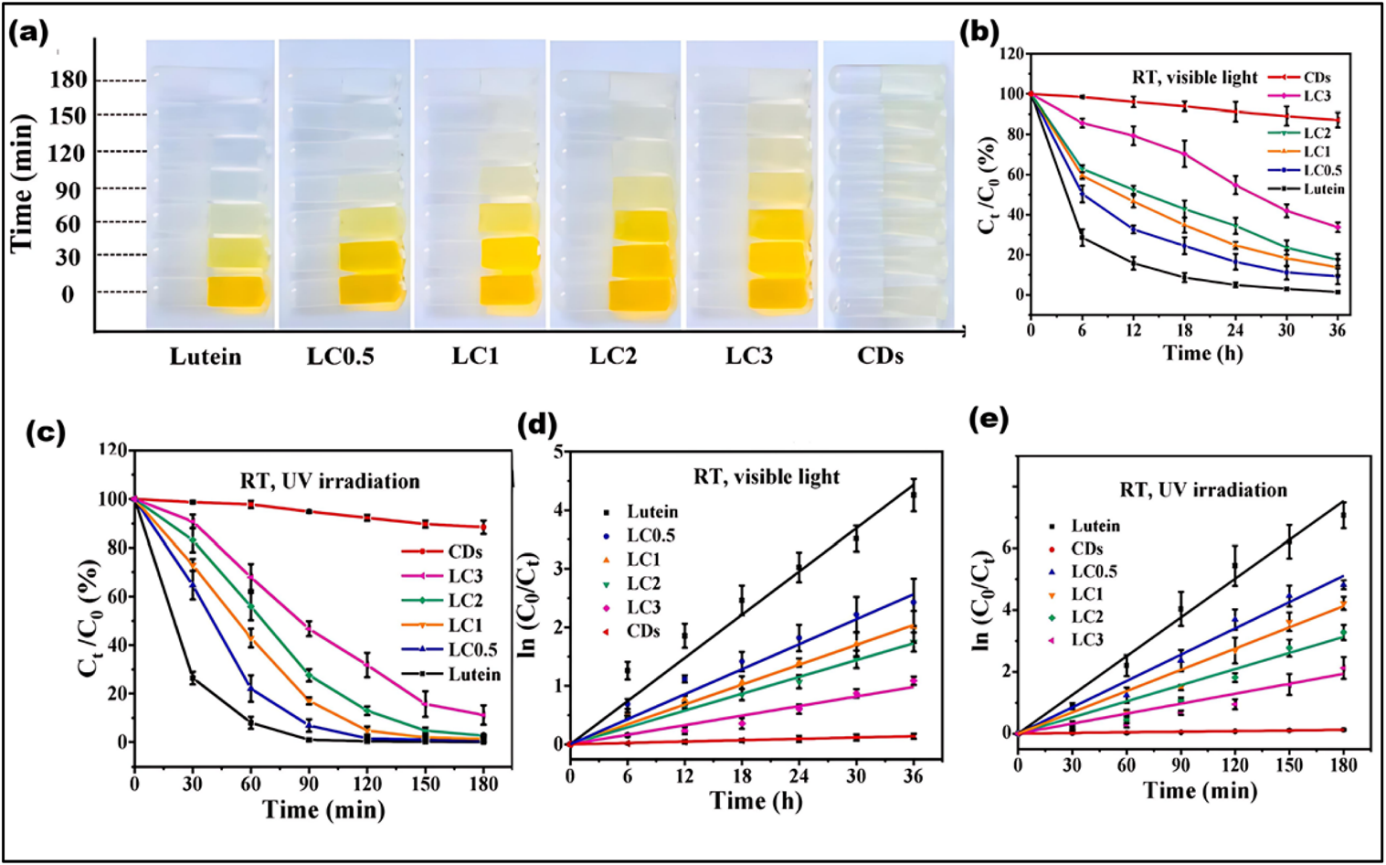
(a) Color varying description of leutin from LC_0.5_–LC_0.3_ under UV irradiation, (b) photo-stability of lutein, CDs, and LCs under Vis light, (c–e) UV irradiation. ((a–e) Adapted with permission from ref. 120 Copyright 2022 Elsevier Ltd).

Photostability is crucial to keep the optical properties and fluorescence intensity of a material under prolonged light exposure, ensuring its stability and reliability. It is particularly valuable in fields such as bioimaging and optoelectronics. In one study Da jian-Jv *et al.* proposed a technique that uses a solvent-ultrasonic process to encapsulate CDs into lutein, improving its photostability and antioxidant capability.^120^ The lutein/CDs composites (LCs) displayed significantly higher photostability compared to pure lutein when exposed to UV irradiation and UV light. Among the different LC ratios, LC_3_ exhibited the highest photostability (Fig. 11a). Under UV irradiation and UV light, LCs showed 3.4 and 3.5 times greater photostability, respectively, compared to lutein alone (Fig. 11b–e).^120^ The stability was achieved by modifying lutein with CDs, and this is anticipated to increase its use in photostability.^120^ In another approach, O-CDs with orange emission were synthesized *via* a solvothermal process. After 30 minutes (min), the synthesized O-CDs demonstrated improved photostability compared to commercial fluorescent dyes, with fluorescence reductions of 24.78%, 13.5%, 7.6%, 21.2%, and 36.3% for MTG, MTDR, Rhodamine (Rho) 123, Hoechst, and BODIPY, respectively. Moreover, the O-CDs' outstanding photostability was verified by their long-term stability, including almost constant fluorescence intensity after 21 days of room temperature storage. They are useful for imaging mitochondrial cells due to these features.^121^ B. Ju *et al.* synthesized yellow-green emission CDs that can be excited without the need for external stimulation. A straightforward solvothermal method was used to fabricate these CDs from chloroform and o-PD. The procedure included heating a mixture of precursors at 160 °C to figure out the photostability of the generated CDs. When the testing period was prolonged by 30 min, it was discovered that there was no discernible change in the fluorescence intensity of nitrogen doped CDs (N-CDs).^122^ Even after a 360 min exposure, a 96% retention of fluorescence intensity was noted.^123^ Because of their exceptional optical qualities, N-CDs can be employed as sophisticated anti-counterfeiting techniques and as invisible ink for storing important data.

Fluorine and nitrogen doped, FN CDs were synthesized that exhibited photo-activated fluorescence intensification and reversible photochromic behavior. When exposed to UV light, the CDs showed a blue shift in emission and an increase in fluorescence intensity. They also demonstrated pressure-triggered aggregation-induced emission (AIE) that persisted after UV exposure ranging from 5 to 30 min. Due to their resistance to photobleaching and these optical properties, the CDs could potentially be useful for fluorescence-based sensing and imaging applications.^124^

### 2.6 Dispersibility and bio-compatibility

The photophysical properties of CDs have garnered significant interest for their bio-applications, where high bio-compatibility and low cellular toxicity are essential prerequisites.^125^ Numerous studies have evaluated CDs across a variety of *in vitro* and *in vivo* models, including human hepatocellular carcinoma cell line SMMC-7721,^126^ human glioblastoma cells LN-229,^127^ normal human kidney cells HK-2,^127^ and human cervical cancer cells HeLa.^128^ Additional models include normal mammary epithelial cells MCF-10 A,^129^ human breast cancer cell lines MCF-7 (ref. 130) and MDA-MB-231 (ref. 130), as well as normal mammary epithelial cells MDA-10 A^130^ and normal porcine kidney cells LLC-PK1.^130^ Studies have also involved Danio rerio zebrafish,^126^ bean sprout cells,^131^ and *E. coli* strain Seattle 1946 (ref. 132), among other biological models.^133^ This type of work highlights the biocompatibility and cytotoxicity of CDs, establishing a foundation for their potential clinical translation. Numerous studies have shown that the manufacturing processes and the precursors used to synthesize CDs influence their hydrophilic and hydrophobic properties. For instance, the oxygen-containing functional groups generated during the synthesis often lead to CDs exhibiting significant hydrophilicity and dispersibility.^134^ The bulk of CDs exhibit notable hydrophilicity and dispersibility as a result of the precursor's production of oxygen-containing functional groups. Additionally, CDs can change from hydrophilicity to hydrophobicity in the other direction by modifying the functional groups on their surface. For example, Hsu *et al.*^134^ introduced a one-pot hydrothermal synthesis method to produce hydrophilic CDs. They proposed that the hydrophilic nature of the resulting CDs is primarily influenced by the types of functional groups present. In contrast, Mitra *et al.*^135^ employed microwave pyrolysis of Pluronic F-68 (PF-68) to synthesize hydrophobic cyclodextrins (HCDs). These HCD polymers demonstrated enhanced water resistance and effective dispersion in organic solvents. In bioimaging and health care, CD's low toxicity is an essential property. The small size of CDs enables their quick removal from human excretory systems, which is largely responsible for their low toxicity. On the other hand, multiple studies have shown that nanoparticles inhaled can reach the brain and cause neurodegenerative changes.^136^ The toxicity of CDs remains a complex issue, largely due to their intricate structure. To address this, researchers often use non-toxic precursors when synthesizing CDs for applications in biomedicine.

## 3. CDs illuminating the intracellular world

The remarkable photophysical properties of CDs-including their tunable fluorescence, high photostability, and sensitivity to environmental changes-are fundamentally determined by their size, surface chemistry, and electronic structure. These features not only distinguish CDs from traditional fluorophores but also underpin their rapidly growing utility in diverse applications. In particular, their outstanding optical characteristics have positioned CDs as highly promising candidates for advanced bioimaging, sensing, and therapeutic technologies. Building on this foundation, the following section explores how these unique photophysical attributes translate into practical applications, with a focus on the role of CDs in innovative cell imaging strategies.

### 3.1 Fluorescent CDs as intracellular imaging probe

**Fig. 12:**
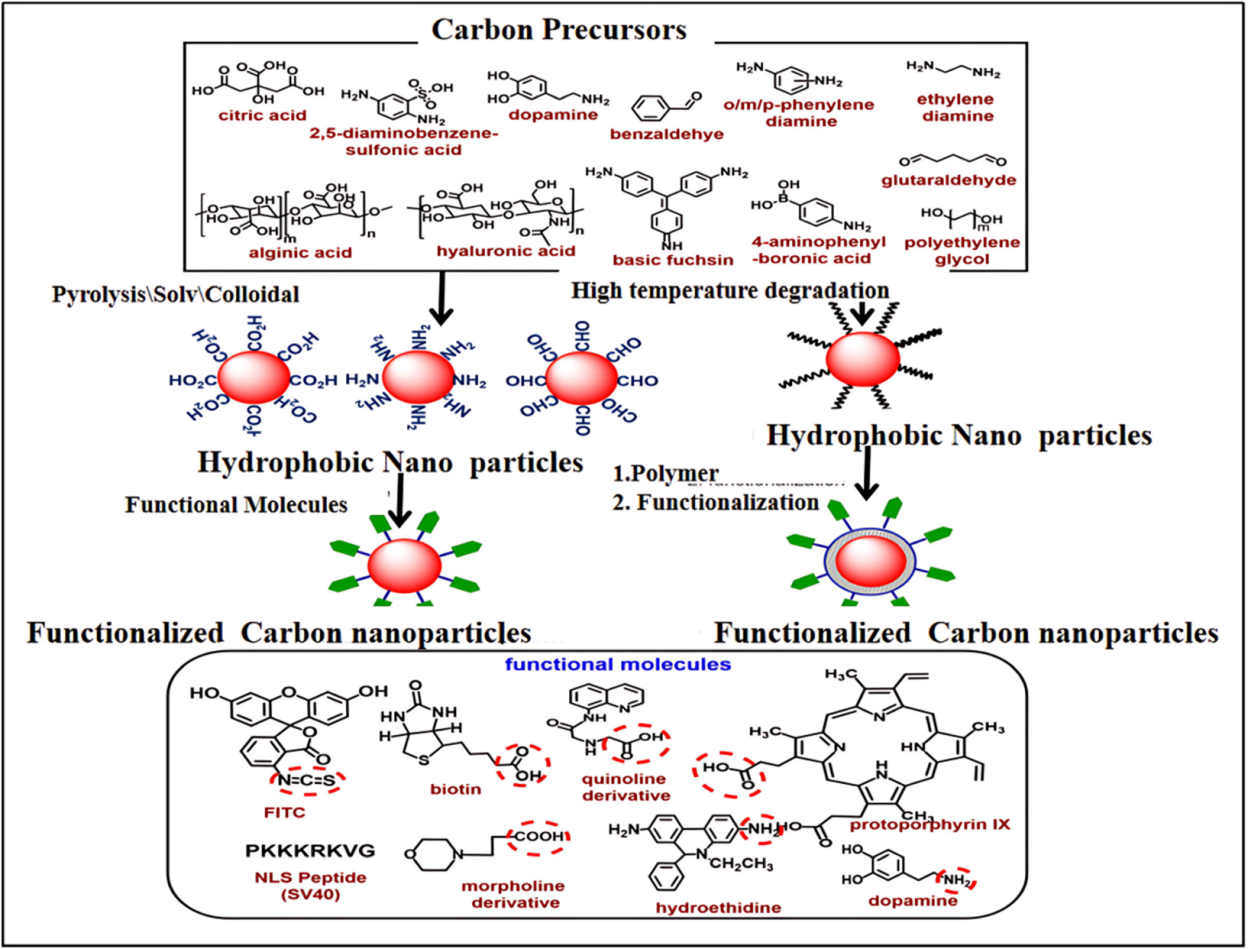
Schematic representation of fluorescent carbon dot synthesis, surface chemistry, and functionalization. The chemical structure of important precursors is shown in the top box. The functionalization approach for hydrophilic CDs *via* conjugation chemistry is shown in the middle left of the scheme and the functionalization approach for hydrophobic CDs *via* coating chemistry and conjugation chemistry is shown in the middle right of the scheme. Chemical structures of selected biomolecules are shown in the lower box that are conjugated with CDs. The highlighted functional groups (red circle) are used for conjugation chemistry. (Adapted with permission from ref. 138 Copyright 2020, Wiley Periodicals, Inc).

Understanding a variety of intracellular processes can be significantly improved through the use of intracellular imaging probes.^137,138^ These particles must be extremely small (preferably less than 10 nm), easily able to enter cells, and able to interact selectively with particular cellular compartments or biomolecules in order to be used in cellular applications. Because of their small size and capacity to target and interact with specific cell components, including lysosomes, mitochondria, and nuclei, as well as a variety of molecules, including protons, metal ions, superoxide species, glutathione, amino acids, and DNA, fluorescent carbon dots, (FCDs), are ideal for this application. Fig. 12 shows a schematic of the synthesis, surface chemistry modification, and functionalization of FCDs. Table 2 provides an overview of the FCDs-based key probes developed for visualizing various intracellular components and compounds.^138^ In this section, we will investigate deeper into some of these possible applications.

**Table 2:** Fluorescent carbon dot-based important probes

Carbon precursor	Conjugated biomolecule, nanoprobe size	Intracellular imaging probe	Ref.
Activated carbon powder	Dopamine, 2–5 nm	Tyrosinase activity monitoring and inhibitor screening	139
Citric acid-ethylene diamine	Nuclear localization sequence peptide (PKKKRKVG), <10 nm	Cell nucleus	140
Hyaluronic acid, branched polyethylene imine	Hyaluronic acid, ∼2–3 nm	Tumor targeting, intracellular imaging, and gene delivery	141
Citric acid-ethylenediamine	Mesoporous silica nanoparticle, ∼200 nm	Redox state	142
Citric acid-polyethylene imine/diamond powder	Morpholine derivative, 2–6 nm	Lysosome	143
*m*-phenylene diamine-l-cysteine	Protoporphyrin IX, 25 nm	Nucleolus, nucleus	144
Triethylene tetraamine-*m*-phenylene diamine/graphite powder/graphite	Lactic acid/porphyrin, 18–25 nm/8 nm	RNA	145
Flour/nanodiamond	Triphenylphosphonium/mitochondria targeting peptide/folic acid, —	Mitochondria	146
Mangifera indica leaves	//	Temperature	147
2-Azidoimidazole	Au nanoparticle, —	Cysteine	148
Nanodiamond	DNA, —	Transfected DNA	149
Nanodiamond	Biotin/integrin β3 antibodies/mitochondrial localization signal peptide, —	Intracellular imaging	82
Basic fuchsin-citric acid/citric acidtridecanediamine/*p*-phenylenediamine/citric acid-dopamine/thiomalic acid	Fluorescein isothiocyanate (FITC)/dopamine, ∼10 nm	pH	150
Ethanol	Hydroethidine, —	Superoxide	151
Alginate-tryptophan	Complexation with Cu^2+^, —	Histidine	152
Citric acid-urea/neutral red-triethyl amine	MnO_2_ nanosheet/MnO_2_ nanoflower, —	Glutathione	153
Capsicum	—, —	Ca^2+^	154
Citric acid-polyethylenimine/citric acidpolyethylenimine	Quinoline derivative, ∼4.5 nm	Zn^2+^	155
Ethanol/(aminopropyl triethoxysilane)	([*N*-(2-amino ethyl)-*N*,*N*,*N*-tris (pyridin-2-ylmethyl) ethane1,2-diamine, —	Cu^2+^	156

### 3.2 CDs for *in vitro* cellular imaging

**Fig. 13:**
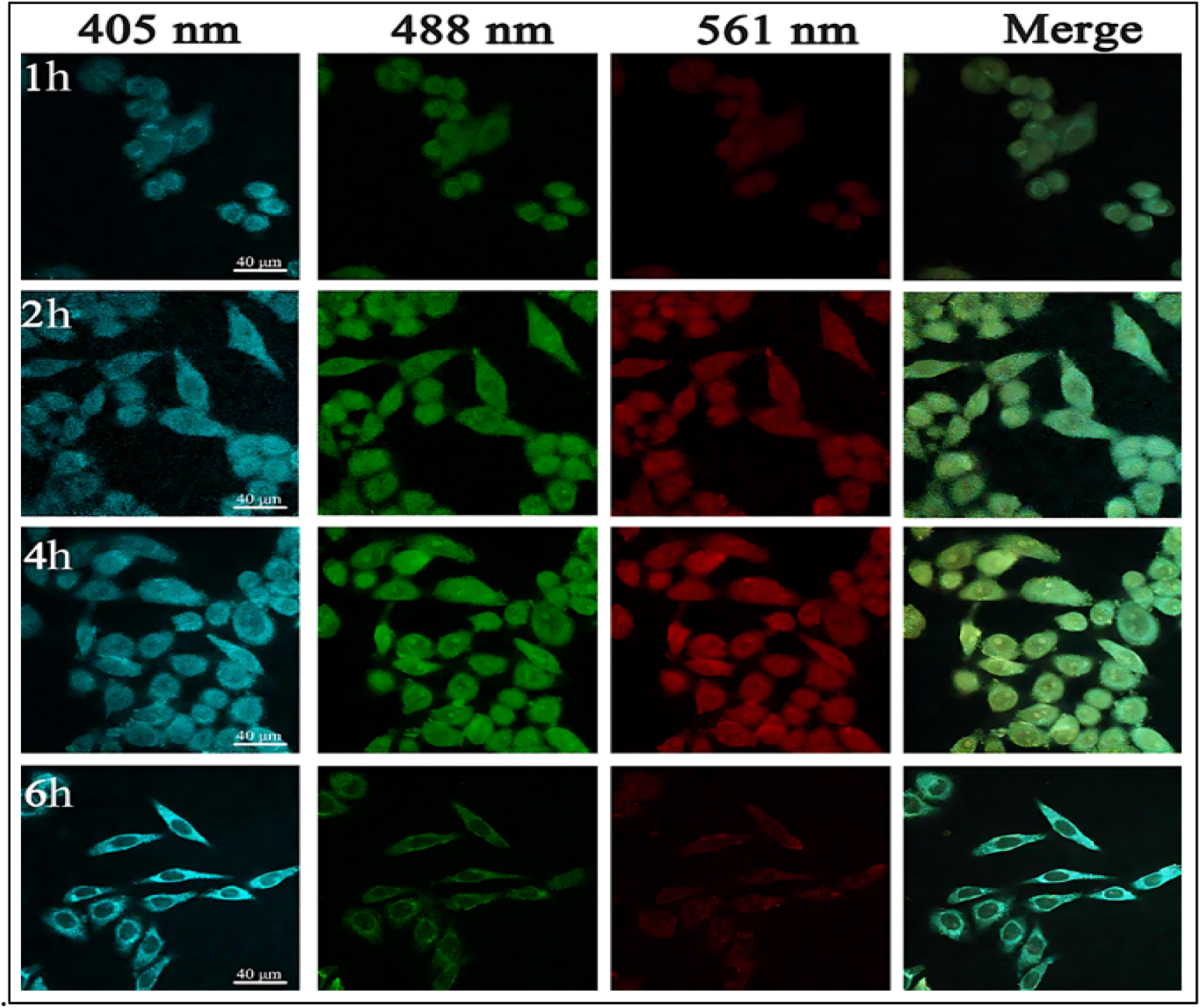
Confocal fluorescence images of HeLa cells treated with prepared CDs for 2, 4, and 6 hour. The 1–4 columns showed different excitation wavelengths at 405, 488, and 561 nm. (Adapted with permission from ref. 162 Copyright 2020 American Chemical Society).

For cell imaging without transfection or ligands, various kinds of CDs having unique optical properties are used.^157^ Endocytosis, aquaporin-aided entrance, and ion channel diffusion are CDs's primary entry mechanisms.^158^ An advanced report proposed citrus lemon as the cheapest means of preparing N-CDs using a hydrothermal process. The MCF7 cells are exposed to 0.025 mg mL^−1^ of the prepared CDs to assess their bioimaging potential. Distinctive blue, green, and red fluorescence in the cytoplasm indicates CDs have entered the sample cells. Cell staining with CDs demonstrated their ability to serve as a versatile probe for imaging whole cells in several colors.^159^ Biocompatibility has been seen in other N-CDs that are made by hydrothermal methods. Fig. 13 depicts HeLa cells that were exposed to CDs at a concentration of 100 µg mL^−1^. The cells were incubated for different periods of time and under various excitation wavelengths. The images captured from this experiment exhibit fluorescence in the colors green, blue, and red. More CDs accumulate within the cytoplasm with longer incubation times, enhancing fluorescence emission. It reveals that CDs may penetrate into HeLa cell membrane.^160^ Scallion-derived N and S-doped CDs were used for examining A549 cells. Most fluorescent light originates from the cytosol's perinuclear regions, according to images. CDs exhibit significant cell permeability in living cells because of their small size and the presence of surface functional groups.^161^ Despite much investigation, the specific method by which cells intake CDs remains unclear.

#### 3.2.1 Subcellular organelles imaging

Subcellular imaging plays an important role in identifying and studying disease mechanisms, cellular growth, repair, and other molecular pathways. This type of imaging focuses on various cell components, such as lysosomes, nuclei, mitochondria, nucleolus, lipid droplets (LDs), and the endoplasmic reticulum (ER). A key challenge in organelle imaging is ensuring that the probe can effectively penetrate the cell and bind to the target. To address this, researchers commonly use accessible probes like Deep Red, LysoTracker, and NucRed Live 647 in co-localization experiments to accurately track the probe locations. The primary objectives of subcellular imaging with CDs are outlined below.

#### 3.2.2 Lysosome

**Fig. 14:**
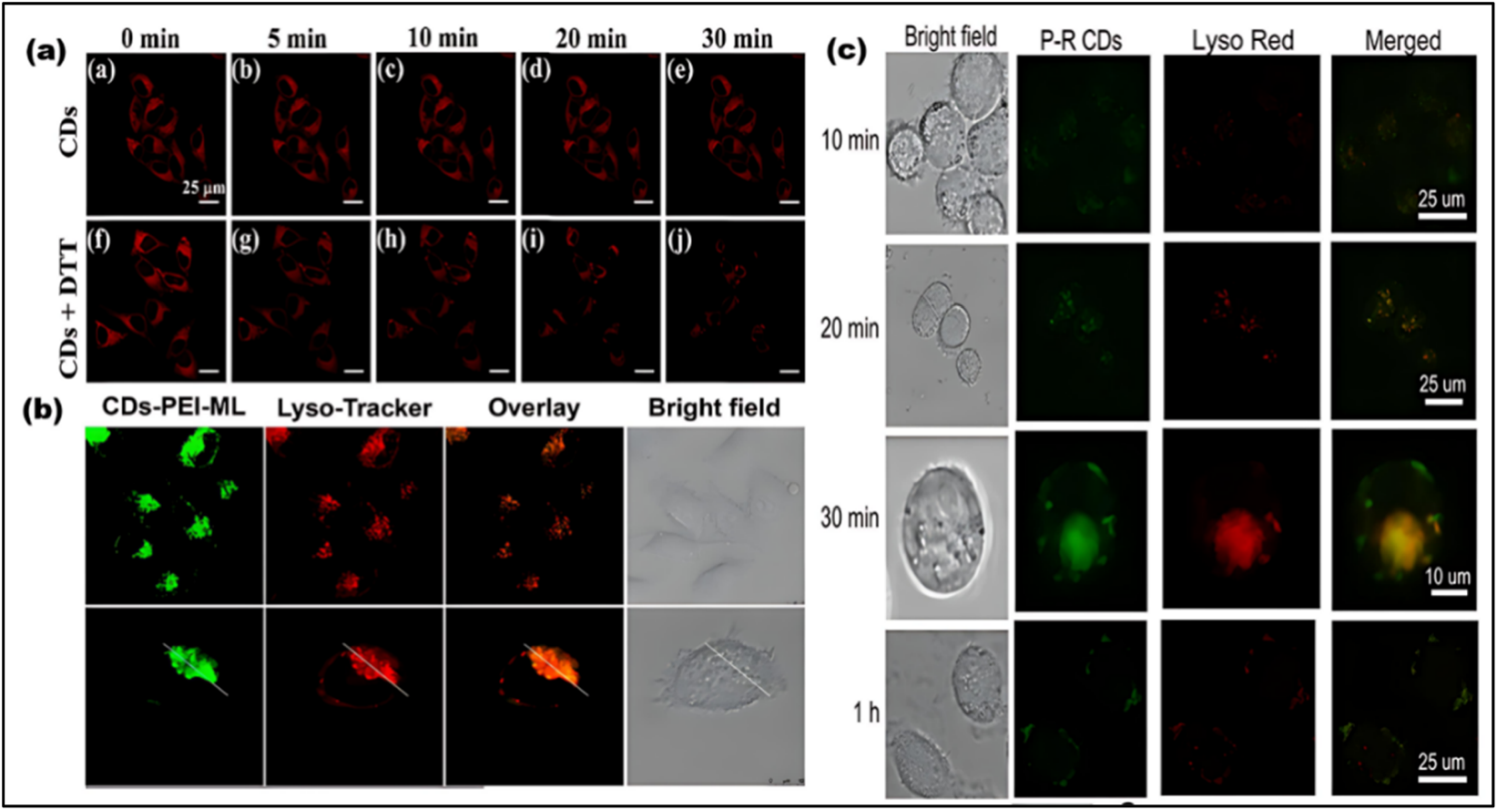
(a) Confocal Laser Scanning Microscopy (CLSM) images of HeLa cells treated with CDs and CDs ^+ ^DTT. (Adapted with permission from ref. 167 Copyright 2020 American Chemical Society). (b) The comparative images of CDs and Lyso-Tracker Red probe in live HeLa cells. (Adapted with permission from ref. 143 Copyright 2017 American Chemical Society). (c) Lysosomal imaging of the CDs in the HL-7702 cell line. (Adapted with permission from ref. 168 Copyright 2020 American Chemical Society).

Lysosomes are spherical, acidic digesting organelles in eukaryotic cells.^163^ Angiogenesis, energy balance, and macromolecular digestion occur in lysosomes.^164^ Cardiovascular, neurological, cancer, and Alzheimer's illnesses arise from lysosome defections.^165^ Researchers have created lysosome-targeting CDs in recent years that effectively use clathrin-mediated endocytosis to target lysosomes.^166^ Lysosomal polarity may assist to understand normal and abnormal lysosome functioning. The CDs also show that dithiothreitol (DTT) causes living cells to shift polarity, reducing fluorescence intensity with increasing polarity (Fig. 14a). The phenyl-CDs tracked lysosome polarity successfully.^167^ According to recent studies, the morpholine group in CDs' chemical structure targets lysosomes particularly. The endocytic route deposits these CDs in the lysosome, enabling long-term tracking in living cells. CD's green emission and LysoTracker Deep dye's red emission generate bright orange fluorescence in live cells (Fig. 14b). Lysosome-stained CDs correlate with LysoTracker Deep Red.^143^ New one-step hydrothermal rose Bengal and PEI CDs indicate remarkable lysosome affinity (Fig. 14c), produced CDs penetrate cells in 10 min. After 30 min, they accumulated in the lysosome and fluoresced strongly. These CDs present a prospective substitute for traditional probes due to their long-lasting imaging capabilities, lack of leakage, remarkably high PL efficiency, and low cytotoxicity.^168^ In summary, CD modification with morpholine and –NH_2_ groups targets lysosomes. But –NH_2_ groups have an affinity with the ER, and previously had difficulties with lysosome specificity. CDs are taken *via* energy-dependent, micropinocytosis, caveolae, and clathrin routes. Different functional groups of CDs can be temporarily trapped in lysosomes.^169^

#### 3.2.3 Nucleus

**Fig. 15:**
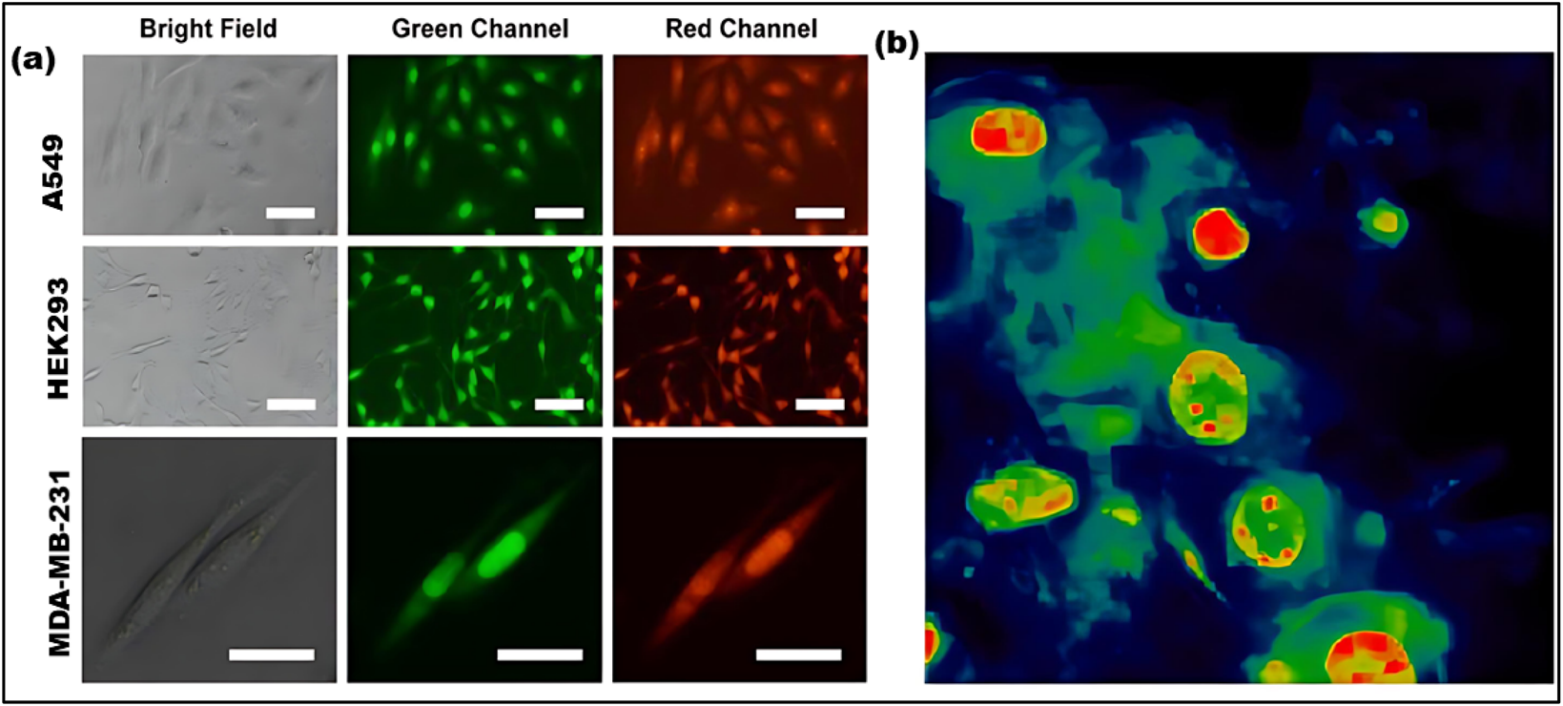
(a) Fluorescence images of A549, HEK293, and MDA-MB-231 cells treated with CDs, fluorescence signal accumulated in nuclear substance, especially in A549 and MDA-MB-231 cells. (Adapted from ref. 171 Copyright 2021 Elsevier). (b) The nuclei of NIH/3T_3_ cells are filled with CDs (red color). False colored maps of intensity. (Adapted with permission from ref. 172 Copyrights 2021 by the authors. Licensee MDPI).

Cell metabolism, activity, and diseases, including cancer, are closely linked to the nucleus. However, scientific understanding of the nucleus is still limited, making nucleus imaging and labeling critical for both biological studies and therapeutic applications. In cancer treatment, for instance, nucleus imaging is essential for gene therapy and drug delivery. CDs can be used not only as stable fluorescent probes for nucleus visualization but also for drug delivery.^170^ CDs produced through a hydrothermal process, are water-soluble and nontoxic. The positive surface charge of these CDs enhances electrostatic interactions with DNA macromolecules in the nucleus, which may account for their ability to target the nucleus (Fig. 15a).^171^ For example, in L929 cells, the fluorescence emission of CDs undergoes a blue shift due to the physicochemical environment, with the lowest intensity observed in the cytoplasm and the highest in the nucleus (green and red, respectively) (Fig. 15b).^172^ Interestingly, positive CDs and negative GO can electrostatically assemble to form a biocompatible probe with low cytoplasmic binding and selective targeting of the nucleus.^173^ This electrostatic interaction between CDs and GO alters the nanostructure electrical charge, enabling precise identification of cell nuclei. While there is no standardized method for nucleus identification using CDs, previous studies suggest that the zwitterionic surfaces of CDs may facilitate nucleus attachment. However, nucleus targeting through the surface functionalization of CDs remains complex and is not yet fully understood, making this area of research both novel and promising for future investigations.

#### 3.2.4 Nucleolus

**Fig. 16:**
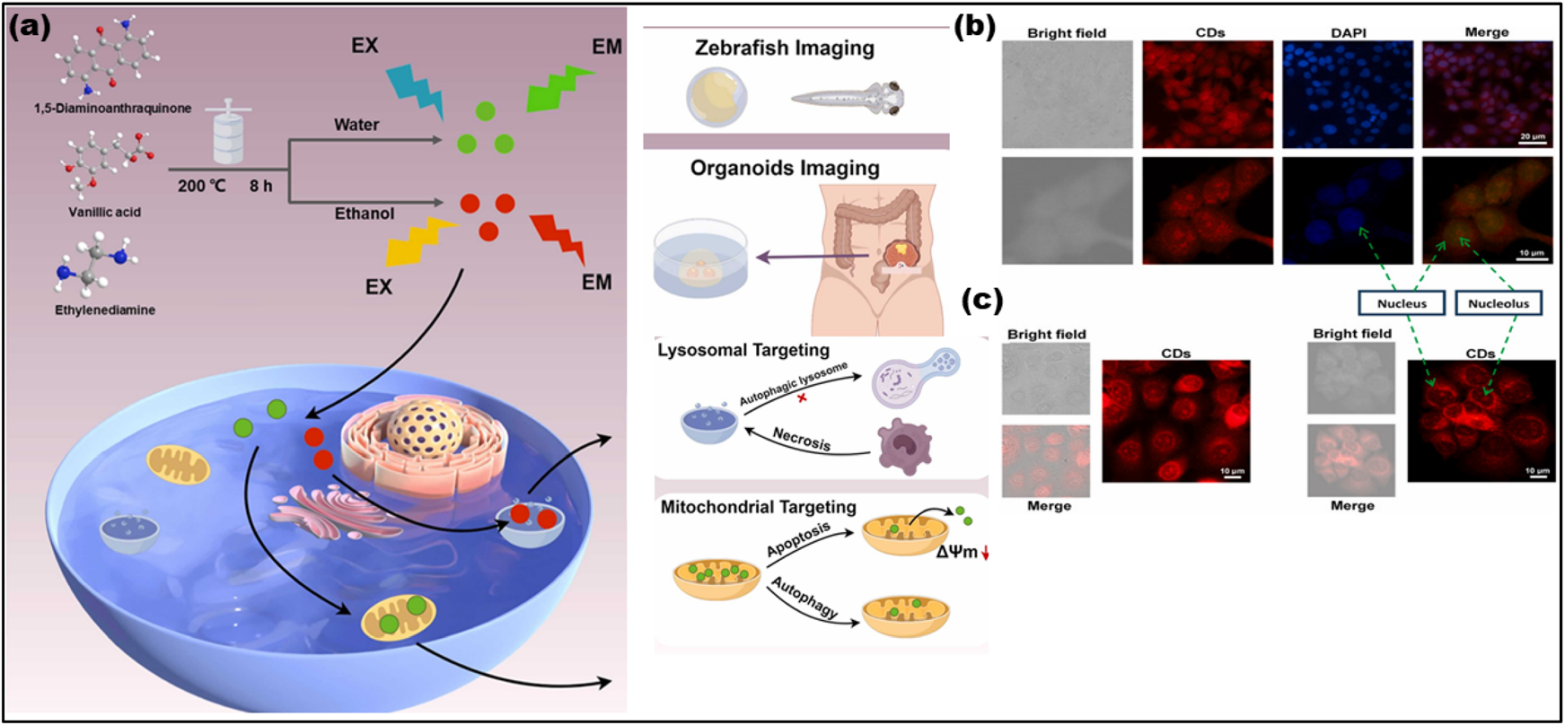
(a) The synthesis of G-CDs and R-CDs and their applications in live-cell dynamic monitoring of mitochondria and lysosomes, as well as in organoid and zebrafish imaging. (Adapted with permission from ref. 183 Copyrights 2024, Elsevier B.V. (b and c)) Inverted fluorescence microscope and confocal laser scanning microscope images of fixed and live MCF-7 cells in the presence of CDs. (Adapted with perfmission from ref. 180 Copyrights 2025, Elsevier Ltd).

The nucleolus is responsible for producing ribosomes, which in turn synthesize all cellular proteins.^174^ Since proteins are essential for the functions of biological components, imaging the nucleolus provides valuable insights into a cell's condition and metabolic processes.^175^ The Common techniques for examining the morphological structure of the nucleolus in fixed cell lines include immunohistochemistry (IHC),^176^ silver staining,^177^ and fluorescence *in situ* hybridization (FISH). Organic fluorophores like silver staining and SYTO RNA^178^ FISH are often used in both fixed and living cell imaging,^179^ although nucleolus imaging can lead to photobleaching of these dyes. Red-emitting CDs for nucleolus imaging in both fixed and live cells were synthesized using a one-step hydrothermal method. By using nicotinamide (NA) and o-PD as precursors, resulting in ultra-narrow bandwidth emission at 617 nm.^180^Fig. 16b presents fluorescence microscopy images of MCF-7 cells. Panel 16(b) shows fixed cells stained with CDs and DAPI, where CDs predominantly target and stain the nucleolus in the cells. Panel 16(c) shows live cells with the same staining pattern, indicating that the CDs can successfully enter the cells and accumulate in the nucleolus, emitting bright red fluorescence under UV light. The figures clearly highlight the selective targeting of the nucleolus, confirming the CDs' ability for precise cellular imaging. Notably, doping CDs with fluorine has resulted in a novel nucleolus and tunneling nanotube (TNT) staining agent, which boasts a 56% QY, low toxicity, resistance to photobleaching and excellent water solubility. The primary reason for using CDs in nucleolus labeling is their non-covalent affinity fornucleolus RNAs and ribosomes,^181^ which facilitates imaging without the need for covalent bonding.^182^ Interestingly, despite their potential for subcellular labeling, CDs have yet to be applied in key nucleolus diagnostics, such as immune activation, transformation, or chemo-drug therapy. This gap highlights the need for further exploration of CDs in nucleolus-based diagnostics, making them an intriguing area of research.

#### 3.2.5 Mitochondrion

The mitochondria release ATP, an essential energy source in live cells, in mammalian cells.^184^ Any mitochondrial imperfections cause cardiac malfunction, Parkinson's and Alzheimer's.^185^ The environment of the mitochondrion is alkaline (pH = 8), with a positive surface charge and a negative interior charge.^186^ Thus, the two methods for targeted imaging of mitochondria include lipophilic labels with positive surface groups such as pyridine, ammonium, and triphenyl phosphonium (TPP), as well as rhodamine modification or targeting peptides.^187^ CDs affinity for mitochondria is practically increased by TPP ligands.^188^ TPP is toxic to many cell types, even with targeted mitochondrial imaging using these CDs. However, efforts are being made to generate CDs with basic mitochondrial targeting.^189^ Scientists have created CDs that are long-lasting, water soluble, photostable, biocompatible and mono-sized. Li *et al.* developed dual-color CDs green (G-CDs) and red (R-CDs) for dynamic fluorescence tracking of mitochondria and lysosomes in living cells. G-CDs, emitting green fluorescence, specifically target mitochondria due to their positive surface charge and good lipophilicity, allowing them to interact with the negatively charged mitochondrial membrane. In contrast, R-CDs, emitting red fluorescence, target lysosomes due to their stronger fluorescence in acidic conditions, which matches the acidic internal environment of lysosomes. These CDs demonstrated excellent fluorescence stability and targeting specificity, making them valuable for monitoring cellular processes like apoptosis, autophagy, and necrosis. The study extended their application to zebrafish and human organoids, demonstrating their potential in *in vivo* imaging. Fig. 16a illustrates the synthesis process and application of these CDs, which can be utilized for advanced imaging in disease modeling and drug screening. This work highlights the potential of CDs as versatile probes for real-time organelle monitoring and biological research.^183^

#### 3.2.6 ER

Protein synthesis, transport, production of lipids and storage, calcium storage and control, and glucose metabolism are all included in the functions of the ER. Therefore, ER mediates cell metabolism and regulation.^190^ Polarity influences different cellular processes and metabolism, which makes it vital for healthy cells. The ER can influence cell polarity and function by protein post-translational modification, transformation, and secretory production of proteins. Thus, ER polarity anomalies may suggest diabetes and Alzheimer's.^191^ Consequently, effective ER imaging gives reliable cell activity and human disease information. Dual emissive CDs for ER labeling are produced by lysine and o-PD. In the o-PD carbonization test, lysine was added to the flask, which slowed down the reaction and turned the green emission blue. CDs enter the ER lumen by lipid raft-mediated endocytosis because they are both lipophilic and electrophilic. Introduced CDs can image ER stress by responding to ER polarity changes in live cells.^192^ In summary, CDs can effectively visualize the ER and offer significant morphological data. The interactions and reactions of CDs within the ER in response to changes in the environment, such as pH levels, provide insights into the functions of the ER and other organelles.

#### 3.2.7 LD

**Fig. 17:**
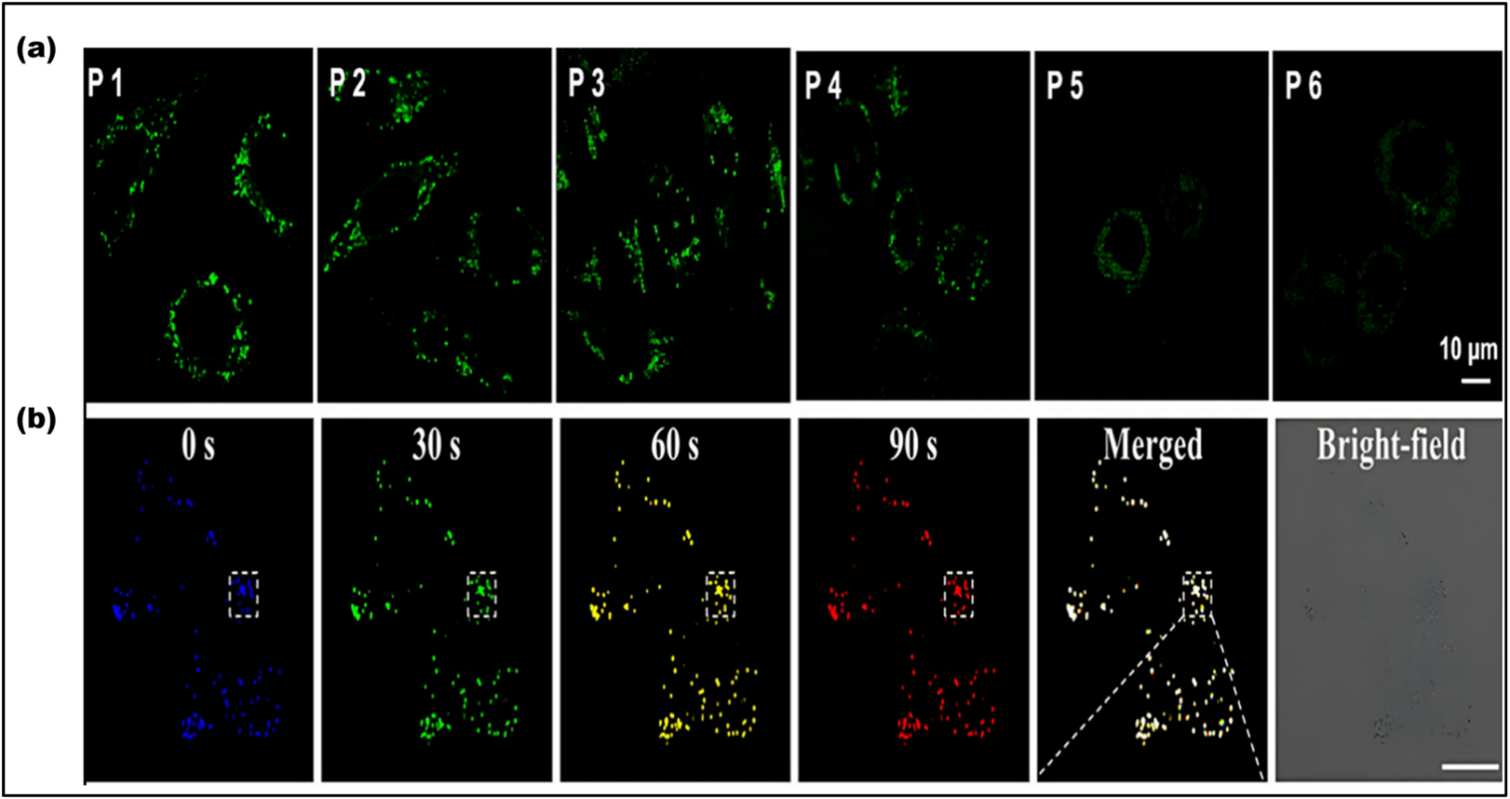
(a) Confocal images of LoVo cells treated with CDs at different passages. CDs: excitation at 405 nm. Adapted with permission from ref. 194. Copyright 2021 American Chemical Society. (b) Monitoring of the LD movement by PA CDs: the movement of LDs at 0, 30, 60, and 90 s is illustrated by four different colors and the merging of all images. (Adapted with permission from ref. 195. Copyright 2021 American Chemical Society).

LDs consist of lipids surrounded by a phospholipid layer, forming hydrophobic cores. Given the various functions of LDs in the cell, there is a strong belief that they play a significant role in lipid metabolic disorders like atherosclerosis, obesity and cancer.^193^ Using CDs to image lipid droplets is a useful method for tracking cellular lipid metabolism and related conditions. The hydrothermal approach was used for the production of biocompatible and amphiphilic CDs for LD imaging. The outstanding cell survival, photostability, ability to stay inside cells, and long-term LD monitoring capabilities of the produced CDs set them apart. These fluorescent CDs remain visible within LDs in the cell cytoplasm even after six cycles (Fig. 17a). The CDs can also be used to monitor the effects of atorvastatin, a common lipid-lowering medication, on hepatocyte cells and allow the tracking of autophagy during the catabolic process.^194^ These CDs are made using the lipophilic reagent 4-piperidinoaniline (PA), which naturally targets LDs. These CDs allow researchers to track changes linked to fatty liver disease and visualize the dynamic behavior of LDs^195^ (Fig. 17b). Table 3 Summarize the application of CDs for intracellular imaging of organelles.

**Table 3:** Summarize the application of CDs for intracellular imaging of organelles

Precursors	Size (nm)	Biocompatibility viability > 80% (mostly by MTT assay)	Fluorogenicity	Brightness	Photoblinking	Red & NIR	Photostability	Imaging target	References
Tris betaine hydrochloride	7	—				●	●	Nucleus	172
*p*-Aminoazobenzene	4	0–100 µg mL^−1^	●	●		●		Nucleolus	181
l-arginin									
Ascorbic acid									
Fluoresceinamine	5–10	MTS assay 100–800 µg mL^−1^					●	pH	196
Styrylcyanine dye *o*-Phenylenediamine	3	0–1 mg mL^−1^					●	Lysosome	197
H_2_0_2_ NH: H_2_0 ammonium citrate	3.5	CCK-8 assay 0–100 µg mL^−1^		●				Cytoplasm	198
Micro-algae dunaliella salina	2–8	0–5 mg mL^−1^				●	●	Ion	199
Trisodium citrate sodiumthiosulfate	5	0–15 mg mL^−1^	●	●		●	●	Small molecule	200
Porcine pancreatic lipase	1–5	0–100 µg mL^−1^		●				Small molecule	201
Methyl blue	2.3	0–2 mg mL^−1^		●	●	●	●	Small molecule	202
Plant Wedelia Trilobata	2.8	CCK-8 assay 50–400 µg mL^−1^				●	●	Small molecule	203
Azure a chloride copper gluconate	4.5	0–5 mg mL^−1^				●	●	Small molecules	204
l-tartaric acid Triethyleneteramine	2.5	0–40 µg mL^−1^		●	●		●	Small moelcules	205
Calcon-carboxylic acid	5.1	—				●		Small molecules	206
Glycerol ethylenediamine	5	0–200 µg mL^−1^			●	●	●	Small molecules	207
*DBP citric acid	21	0–5 nM			●			Proteins	208
Urea citric acid	2.5		●		●	●		Proteins	209

Building upon the extensive *in vitro* analyses, it is important to consider the growing body of *in vivo* research, which provides critical insights into the biodistribution, toxicity, and biocompatibility of CDs in living organisms and their translational potential for biomedical applications.

### 3.3 *In vivo* evaluation of CDs

*In vitro* cytotoxicity assessments of diverse pristine and functionalized CDs have generally revealed encouraging biocompatibility, with cell viability typically ranging from 80% to 95% or higher, even at notably high concentrations (1000 µg mL^−1^).^133^ To build upon the *in vitro* investigations and foster a more thorough understanding of the potential therapeutic uses of CDs, *in vivo* studies have been performed primarily utilizing well-established murine and zebrafish models. In 2009, Ya-Ping Sun *et al.* carried out the initial optical study to assess the *in vivo* toxicity of CDs by injecting nanoparticles into a group of mice. The results indicated that the animals showed no signs of acute toxicological effects, suggesting that CDs were non-toxic *in vivo*.^210^

In 2018, Pengfei Wang *et al.* synthesized CDs from *Hypocrella bambusae* (HBCDs), a fungus with potential therapeutic applications for rheumatoid arthritis, skin, and gastric diseases. The solvothermal method was used to prepare HBCDs that exhibited red-light emission and good water solubility. These HBCDs, at a concentration of 2000 µg mL^−1^, were then intravenously injected into groups of mice with body weights ranging from 15 to 20 g, some with induced tumors and others without tumors. The fluorescence signal in tumors reached a maximum after 8 hours and then decreased gradually, being completely removed after 14 days. The accumulation of HBCDs was indicated through the enhanced permeability and retention (EPR) effect, with the highest accumulation level found in the liver and kidney. Monitoring the mice's body weight at various time points post-injection revealed no sudden changes. A histological comparison of the major organs also showed no alterations, indicating that HBCDs have very low biotoxicity and hold promise for biological applications.^211^ In a 2019 study by Saxena *et al.*, N-doped carbon quantum dots (NCQDs) were produced using d-glucose and ethylenediamine *via* microwave irradiation. Swiss albino mice, each weighing around 22 g, received intravenous injections of NCQDs at doses of 5 and 10 mg kg^−1^ body weight. After observing the mice for 30 days, the researchers recorded an increase in body weight (exceeding 25 g), with no significant changes noted in major organ weights or protein content in tissues. Based on additional assessments of antioxidant, hematological, and histopathological parameters, the researchers concluded that NCQDs exhibit favorable biocompatibility.^212^ Multiple studies have shown that the incorporation of CDs into zebrafish does not lead to embryonic toxicity. In 2015 study conducted by Xue-Bo Yin *et al.*^213^ it was demonstrated that CQDs introduced into zebrafish embryos were non-toxic. The CQDs were synthesized through a hydrothermal method using glucose and EDA as precursors, and they were administered to zebrafish embryos and larvae *via* soaking and microinjection techniques. Both embryos and larvae showed significant tolerance to high concentrations of CQDs. For embryos, survival rates were over 80% at 24 hours post-fertilization (hpf) when microinjected with 1.5 mg mL^−1^ or soaked in concentrations up to 1.5 mg mL^−1^, although this rate decreased to approximately 55–60% at higher concentrations. For larvae, survival remained unaffected at 84 hpf when exposed to concentrations up to 625 µg mL^−1^ of CQDs, but decreased to over 85% and 55% at 1.25 and 2.5 mg mL^−1^, respectively.^213^ In 2020, Tongkai Chen *et al.* produced CDs using a hydrothermal method with sugarcane molasses. Toxicity assessments on zebrafish embryos exposed to CDs at concentrations ranging from 50 to 400 µg mL^−1^ indicated that there were no significant embryonic toxic effects or teratogenic outcomes at levels up to 150 µg mL^−1^. The study established the LC50 and TD50 values for CDs at 96 hours post-fertilization (hpf) as 257.2 µg mL^−1^ and 194.7 µg mL^−1^, respectively.^214^ These findings suggest excellent biocompatibility, especially considering that the US FWS Acute-Toxicity Rating Scales classify an aquatic 96 h LC50 value of 100–1000 mg L^−1^ as ‘Practically Nontoxic’.^215^ However, notable adverse effects were documented at concentrations exceeding 200 µg mL^−1^, including pericardial and yolk sac edema, delayed growth, spinal cord flexure, and mortality. Additionally, high concentrations of CDs were linked to decreased larval locomotor activity, lower dopamine levels, reduced tyrosine hydroxylase-positive dopaminergic neurons, and damage to multiple organs in zebrafish embryos.^214^ The mechanisms behind these adverse effects at elevated CD concentrations remain unclear and require further exploration. Furthermore, photodegradation of CDs may introduce some degree of cytotoxicity, irrespective of their chemical composition. Liu *et al.* proposed that light exposure could provoke cytotoxicity in CDs, as many cytotoxicity studies have been conducted in the absence of light. Experiments carried out by their team on both healthy and cancerous cell lines indicated that irradiated, lab-synthesized CDs exhibited toxicity in both types of cells. Among the 1431 photolyzed molecules, 499 were linked to cytotoxicity and may contain benzene, –C=O or –OH groups.^216^ Another important factor for the *in vivo* use of carbon dots (CDs) is their timely elimination from the body.^217^ In this context, Chen *et al.*,^218^ conducted a study where they conjugated CDs with the ZW800 dye and administered the compound to mice *via* intravenous (tail vein), intramuscular (left leg muscle), and subcutaneous (left leg skin) injections. The results indicated that CDs were quickly eliminated from the mice, with clearance rates observed in the following order: intravenous > intramuscular > subcutaneous.^218,219^ After administering the CDs-ZW800, the researchers assessed the clearance using ex vivo imaging of the kidneys and livers of the mice, along with positron emission tomography. Both imaging methods showed no signs of CDs-ZW800 accumulation 24 hours post-injection, confirming the compound's elimination.^218^

Overall, these findings suggest that CDs demonstrate promising biocompatibility, indicating their potential for various bioapplications. However, concerns regarding neurotoxicity and cytotoxicity due to photodegradation highlight the need for further research to ensure the safe and effective use of CDs.While CDs have demonstrated significant promise as fluorescent probes for cell imaging due to their tunable emission and biocompatibility, conventional intensity-based imaging techniques still face challenges such as photobleaching, background autofluorescence, and limited multiplexing capability. These limitations highlight the need for more advanced imaging modalities that can fully exploit the unique photophysical properties of CDs One such advanced technique is fluorescence FLIM, which leverages the fluorescence lifetime parameter-rather than intensity-to provide more robust, quantitative, and environment-sensitive imaging. Understanding the fundamental principles of FLIM is essential to appreciate how its integration with CDs can overcome the shortcomings of traditional fluorescence imaging and unlock new possibilities in cellular visualization.

## 4. FLIM in cellular imaging: advancements and applications

FLIM is an advanced imaging technique that enables the measurement of fluorescence lifetimes at the pixel level, yielding precise spatial distributions of these lifetimes inside an object. When a molecule absorbs light, its electrons are elevated to a higher energy level and, upon reverting to the ground state, emit energy as fluorescence. The fluorescence lifespan, or tau (*τ*), is the average amount of time a fluorophore stays excited before relaxing. The usual *τ* ranges from femtoseconds to several nanoseconds.^21,220^ In some cases, fluorophores exhibit longer decay times, ranging from microseconds to milliseconds. These extended *τ* may involve additional electronic transitions, such as phosphorescence.^221^ The *τ* is a key characteristic of a fluorescent molecule and is influenced by a range of physical processes, including solvent effects, excited-state reactions, quenching, photolysis, molecular interactions, and internal conversion. Unlike fluorescence intensity, which can vary based on factors like concentration or instrument settings (*e.g.*, excitation source, wavelength, and optical path), the fluorescence lifetime is a relatively stable parameter.^222^

Notably, some fluorophores, such as aggregation-induced emission (AIE) fluorophores, exhibit concentration-dependent lifetime changes. These changes make FLIM an excellent tool for studying AIE phenomena, where the fluorescence lifetime can alter due to molecular aggregation. While traditional intensity-based imaging provides some insight into fluorescence characteristics, it does not offer the same depth of information as FLIM. FLIM enhances traditional methods by integrating both temporal and spatial data on fluorescence lifetimes, allowing for more nuanced and comprehensive analysis of fluorescence behaviors.^223^ By measuring the timing of photon arrivals following excitation, FLIM offers a significant improvement over intensity-based approaches by recording decay dynamics across multiple absorption and emission cycles.^224^ FLIM usually conducts statistical lifetime profiles of fluorescence analysis on massive populations of fluorophores by combining data from several absorption and emission cycles. However, recent developments in single-molecule FLIM methods have made it possible to estimate fluorescence lifetimes down to the molecular level, which opens up exciting new avenues for exploring molecular interactions at a fine spatial resolution. Therefore, FLIM is a vital tool for studying complicated biological processes since it maps the distribution of fluorescence lifetimes across biological samples.^225^

FLIM is now being used to examine dynamic biological processes such as protein–protein interactions in living cells, in addition to simple molecular mapping. It is possible to see very small things happening inside cells using FLIM because it has a high spatial precision and can measure fluorescence lifetimes. This is why FLIM is so important for cell imaging, particularly for tracking biochemical activities as they happen in real time. A strong grasp of fluorescence lifetime measurements is crucial for efficiently utilizing FLIM for such applications. First, the system is calibrated using fluorescence lifetime standards to ensure accurate measurements.^220^ Then, biological samples are collected and FLIM data is analyzed to extract fluorescence lifetime information.^226^ Finally, the results are interpreted to reveal biological activity or achieve other experimental objective.^227^ The methodology and findings from such experiments are discussed in detail in the literature,^226^ highlighting FLIM's growing importance in the field of fluorescence microscopy.

To further explore the principles and applications of FLIM, a wealth of educational resources is available for readers. Comprehensive guides and tutorials can provide in-depth insights into both the theoretical foundations and practical implementations of FLIM. Notable resources includethe detailed Leica Microsystems guide on FLIM techniques (https://www.leica-microsystems.com), Edinburgh Instruments' overview of FLIM's use in tissue imaging (https://www.edinst.com) and the University of Queensland training manual on FLIM theory and software tools such as FLIMfit (https://www.uq.edu.au). These resources are excellent for those wishing to deepen their understanding of FLIM and its diverse applications in cellular and molecular imaging.

### 4.1 Fundamental principles and methods

**Fig. 18:**
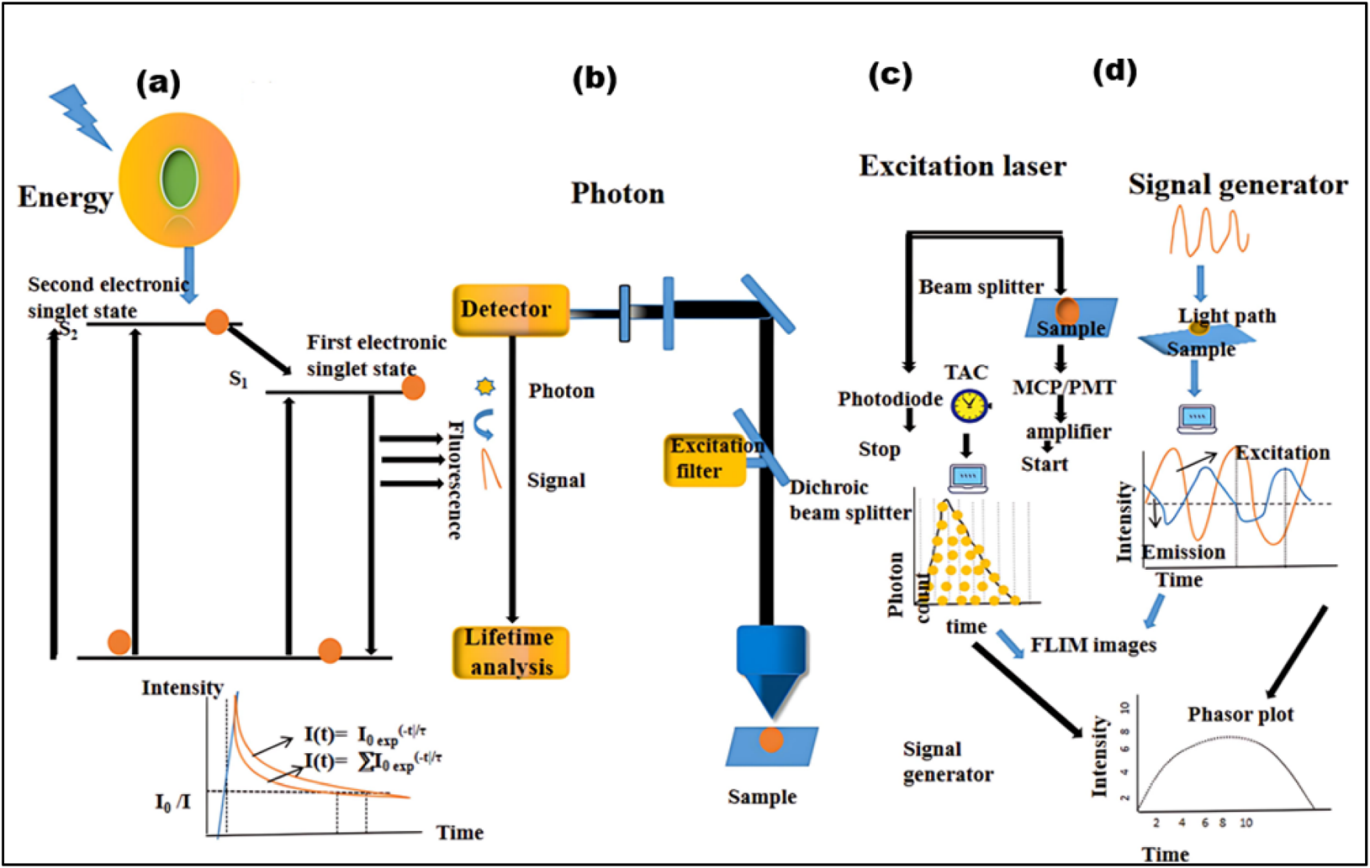
Basic principles of fluorescence lifetime, fluorescence imaging, TCSPC-FLIM, and FD-FLIM. (a) Principles of fluorescence lifetime (b) basic setup for fluorescence imaging with a lifetime analysis instrument. (c) TCSPC-FLIM system scheme. (d) FD-FLIM sketch map.

FLIM involves the measurement of *τ* in each pixel of an image, providing detailed information about the temporal properties of fluorescence. A typical FLIM measurement involves monitoring how the fluorescence intensity decays over time after excitation (Fig. 18a and b). For a single fluorophore, the decay of fluorescence from the excited state to the ground state generally follows an exponential distribution. The fluorescence intensity, *I*(*t*), as a function of time, can be described by the eqn (I)^228,229^

I *I*(*t*) = *I*_0_*e*^−*t*/*τ*^

where: *I*(*t*) is the fluorescence intensity at time *t*, *I*_0_ is the initial intensity at *t* = 0,^225^ This model is commonly used when analyzing a single fluorophore. However,in more complex systems, multiple decay components may be present, necessitating more sophisticated models to describe the fluorescence decay accurately.

In systems with multiple fluorophores, the total fluorescence intensity at time *t* is the sum of the intensities contributed by each individual species, where each species contributes with its own characteristic lifetime. This can be expressed as in eqn (II):II
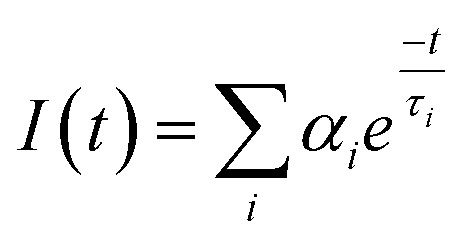


The overall lifetime is determined by the weighted contributions of different fluorophores and can be influenced by radiative and non-radiative decay processes. Non-radiative effects, such as dynamic quenching through molecular collisions with small soluble molecules like oxygen or ions, as well as energy transfer between interacting molecules through Förster Resonance Energy Transfer (FRET), lead to a reduction in *τ*.

FLIM has been measured using time-domain and frequency-domain methods, but advances in technology have blurred the distinction between them. Modern FLIM techniques are categorized into time-tagging and phase-modulation shift methods. Time-correlated single-photon counting (TCSPC) and digital frequency domain (DFD) techniques precisely capture photon arrival times relative to the excitation pulse, enabling accurate fluorescence decay measurements and lifetime calculations. In contrast, the phase-modulation shift approach, also known as the analog frequency-domain technique, employs modulated light sources to record photon arrivals using phase shifts and demodulation. FLIM data acquisition involves collecting multiple excitation–emission cycles to reconstruct a fluorescence decay histogram for each pixel, enabling pixel-by-pixel characterization of fluorescent molecular species. The phasor approach has emerged as a powerful and widely adopted FLIM analysis method due to its simplicity, as it does not require computational models while still offering robust and detailed characterization. Its accessibility has been further enhanced by the availability of open-source tools, leading to widespread adoption in microscopy research.

Notably, it requires less computing power and has no models. Because of this, phasor analysis is now generally accepted in the microscopy field, and nearly all organizations employ it. The abundance of open-source tools available further improves phasor analysis's implementation and accessibility. FLIM is a flexible method that may be used to multiplex numerous fluorophores inside a sample and investigate a range of biological processes, such as tissue and cellular metabolism,.^230–234^ It is now possible to obtain FLIM pictures in wide-field or scanning mode using both TD and FD FLIM techniques and spectral information. These images can give 3D information (Fig. 18c and d). Further expanding FLIM's spatiotemporal capabilities and facilitating more thorough and in-depth analysis is the combination of FLIM with sophisticated microscopy methods including fluorescence fluctuation spectroscopy, hyperspectral imaging, and stimulated emission depletion (STED).

FLIM allows for the real-time tracking of ion concentrations, protein interactions, and molecular dynamics, among its many other uses in cellular imaging. It holds significant value in metabolic imaging for the surveillance of essential compounds such as FAD and NADH, in addition to its applications in fluorophore multiplexing and spectral unmixing.^235^ When combined with super-resolution techniques like STED, FLIM boosts spatial resolution, allowing for the precise examination of subcellular structures. Furthermore, FLIM-FRET yields essential insights into molecular interactions and localized environmental alterations, enhancing comprehension of cellular processes.^236^

### 4.2 Metabolic imaging

FLIM is a sophisticated method that detects a fluorescence lifetime of material and is extremely sensitive to the surroundings and conformations of molecules. It permits concentration-independent imaging, which can be especially helpful in lowering concentration-dependent toxicity because the lifetime value is independent of concentration. Multiphoton FLIM provides sub-micron sensitivity in 3D tumor spheroid models by improving axial resolution and penetration depth.^237^ This feature enables the measurement of fluorescence decays in both free and bound forms of auto-fluorescent biological components such as FAD and NAD(P)H.^238^ The metabolic pathways that cancer cells use to sustain different cellular processes, such as glycolysis and oxidative phosphorylation, are directly revealed by such measures. By calculating the ratio of bound NAD(P)H to bound FAD, the FLIM Redox Ratio (FLIRR) offers important insights into the main metabolic route of a cell. There is a shift toward oxidative phosphorylation with a greater FLIRR and a movement toward glycolysis with a lower FLIRR.^239^ Likewise, the sensitivity of FLIM to carcinogenicity can also be used to directly visualize tumor heterogeneity, such as differences in solid tumor metabolism and post-treatment metabolic pathway alterations.^240^ The FLIM-based method also presents an intriguing chance to measure the auto fluorescence lifespan of normal and malignant cells. This approach can be used to distinguish cancerous cells from non-cancerous ones because studies have revealed that the fluorescence lifespan of NADH and FAD is shorter in diseased cells than in normal cells.^241^

So it has been investigated as a potent instrument that can diagnose cancer and learn more about cellular metabolism.^235^ This method offers useful insights into the functioning of cells yet maintains the material characteristics. A number of variables, including temperature, pH, ion concentration, and interactions between biomolecules, can affect optical properties, especially lifespan values. These characteristics can be tracked inside cells using FLIM, providing information on treatment effectiveness, cellular functions, and the progression of disease.

It is a powerful technique for assessing tissue metabolism through NADH autofluorescence.^242–244^ By providing real-time,label-free measurements of key metabolic parameters, FLIM is particularly advantageous for metabolic phenotyping. These parameters include the autofluorescence intensity ratio of NADH to FAD and the balance between NADH and NAD+. The ratio of NAD + to NADH, along with the distribution of free *versus* protein-bound NADH, serves as a valuable optical redox marker for evaluating metabolic shifts.^245^ An increase in free NADH levels corresponds to a shift from oxidative phosphorylation to glycolysis. To measure NADH emission, autofluorescence is captured using a narrow filter (440 ± 45 nm). Free NADH has a fluorescence lifetime of approximately 0.4 ns, while protein-bound NADH, such as when attached to lactate dehydrogenase exhibits an extended lifetime of around 3.4 ns. The relative proportions of free and bound NADH are reflected in the autofluorescence lifetime decay^246^ when no additional fluorophores are present^247^

**Fig. 19:**
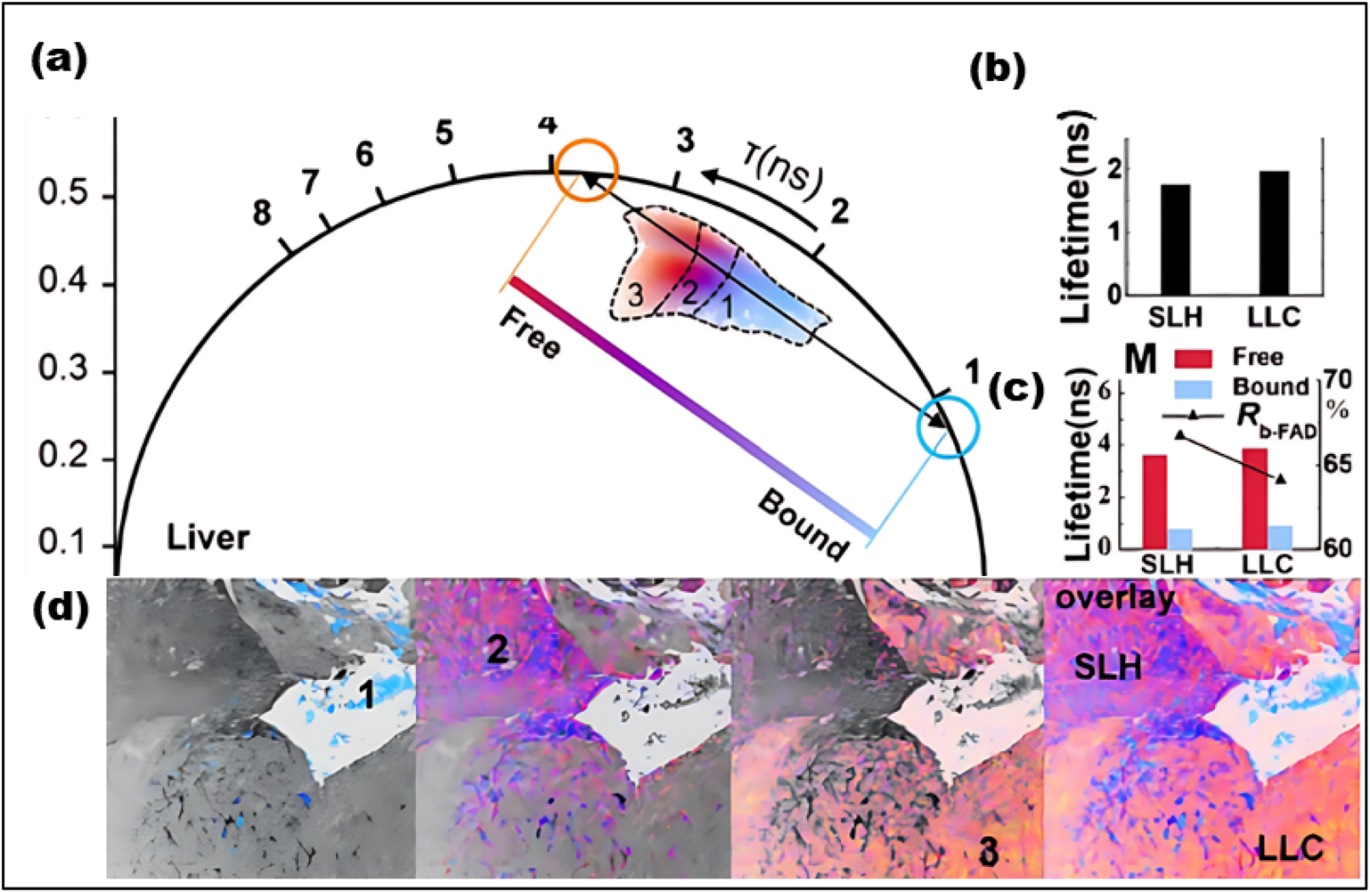
(a) Phasor plot (with different pseudo colors) of cancer cells based on FAD FLIM data. (b) Overall lifetimes. (c) Component lifetimes and Rb-FAD ratios. (d) The regions marked as (1, 2, and 3) represent various lifetime species shown in the pseudocolor images below. ((a–d)Adapted with permission from ref. 248. Copyright 2020, Ivyspring International).

By using FLIM and phasor plot analysis, Shen *et al.* investigated metastatic colonization in pancreatic cancer through FAD autofluorescence (Fig. 19).^248^ Phasor-based FLIM detected fluorophore fractions corresponding to both free and protein-bound FAD, and a two-component analysis of the phasor plot determined their relative proportions. A metabolic shift from oxidative phosphorylation to glycolysis was indicated by an increase in the free FAD fraction along the metabolic trajectory. However, to the best of our knowledge, carbon dots have not yet been widely explored in combination with FLIM for metabolic imaging. We believe that this could represent an exciting new direction for research.

### 4.3 Multiplexing and unmixing of multiple fluorophores

**Fig. 20:**
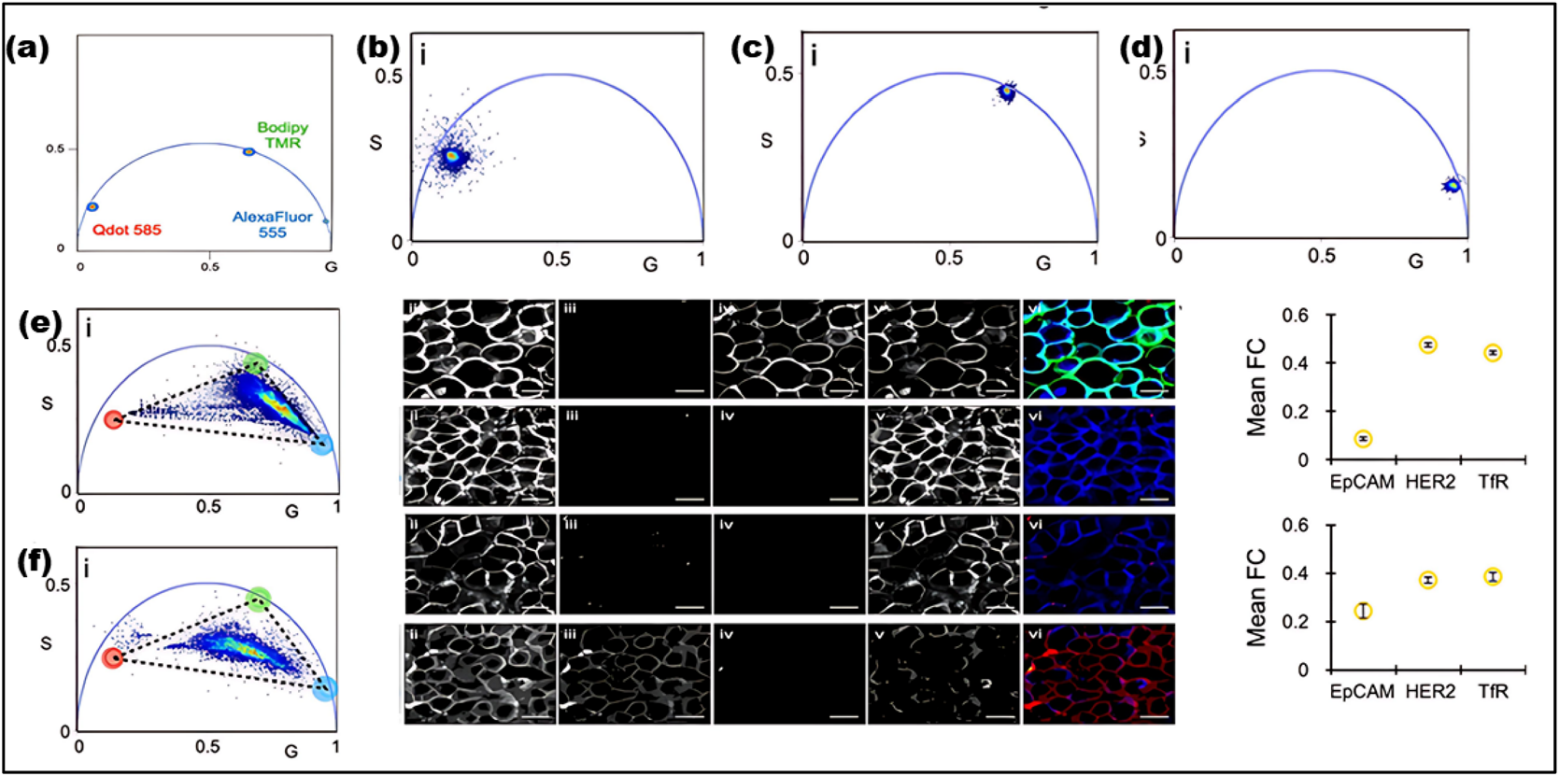
(a) Phasor plots of the three fluorescence lifetime probes. (b–d) The phasor plot of MCF-7 cells was labeled with (b) αEpCAM-Qdot 585, (c) αCK18-Bodipy TMR, and (d) αKi67-AF555. (e) and (f) Adding unlabeled antibodies reduced the signal intensities of HER2-Bodipy TMR and TfR-AF555 probes at labeled/unlabeled ratios of (e) 30 : 70 and (f) 10 : 90. The displayed results include (i) a phasor plot; (ii) an intensity image; (iii)–(v) separate intensity images for EpCAM, HER2, and TfR; (vi) a pseudo colored overlay image (EpCAM: red, HER2: green, TfR: blue); and (vii) the overall fractional contribution of each probe throughout various images. ((a–f) Adapted with permission from ref. 252 Copyright 2022, ACS Publications).

One significant benefit of phasor analysis is its ability to resolve fluorescence species from a material, yielding important biological information in numerous situations.^249,250^ Without making any assumptions about the phasor positions or decay characteristics of the species combined in a single pixel, the blind unmixing method allows for quantitative examination of their contributions. The technique relies on leveraging DFD contemporary electronics^251^ to compute the phasor plot at higher harmonics. Tissue metabolic imaging has been accomplished by the use of blind unmixing. This method makes it possible to quantitatively analyze the fractions of oxidized lipids (long lifetime species), bound NADH, and free NADH. These fractions can be used to compute the ratio of free-to-protein-bound NADH when oxidized lipids are present. Exogenous probes are useful for analyzing the spatial distribution of fluorescence lifetimes in FLIM. By performing metabolic imaging in tissue *via* blind unmixing, the various components—such as free NADH, bound NADH, and oxidized lipids (long-lifetime species)—can be quantitatively analyzed. The ratio of free to protein-bound NADH in the presence of oxidized lipids can be computed by figuring out the proportions of these components. This technique offers a strong tool for evaluating tissue interactions and metabolic processes. Multiplexed detection, such as using probes that target distinct biomolecules, is made possible by multi-component analysis of the phasor plot. Multiple biomarkers on or within cell surfaces can be identified simultaneously thanks to the phasor plot's ability to distinguish between various probes based on their varying lifetimes. Three dyes coupled to antibodies with stable lifetimes were able to successfully identify a variety of biomarkers found on or within the cell surface (Fig. 20).^252^ The group used three dyes with distinct lifetimes that were bio-conjugated to antibodies that targeted various parts of the cell. This method demonstrated the phasor plot's ability to identify three spatially colocalized biomarkers and differentiate between different lifetimes by effectively resolving and quantifying three probes that target cell surface biomarkers. Up to eight distinct biological targets can be detected in living mammalian cell lines using fluorescence lifetime multiplexing.^253^

Using a machine-learning clustering methodology called the Gaussian mixture model (GMM), fluorescent species populations can be automatically and independently recognized through spectrum and lifetime imaging by looking for clusters in phasor space.^254^ MOSAICA (multi-omic single-scan assay with integrated combinatorial analysis) is a spatial-omics technology based on fluorescence imaging that was developed using this method.^255^ By combining FLIM and spectrum imaging with *in situ* tagging of mRNA and protein markers using combinatorial fluorescent probes that have different lifetimes and spectra, MOSAICA allows multiplexed biomarker profiling of cells and tissues.

### 4.4 Local environment sensing

Water dynamics at various subcellular sites are essential for regulating cellular functions such phase separation, membrane dynamics, and enzyme activity. Nevertheless, there are few and technically difficult ways to investigate water dynamics *in vivo* at the subcellular level. Dimethylaminonaphthalene has been used to create environmentally sensitive fluorescent probes that can detect polarity and dipolar relaxation nearby, offering information on the structural dynamics of macromolecules.^256^ The physical state of the membrane phase and the amount of cholesterol present affect the lifespan of LAURDAN, a membrane-affinity probe.^21^ FLIM and phasor analysis have been used to investigate membrane dynamics in living cells by taking use of these characteristics. Dipolar relaxation, shown as phasor clusters, can be easily identified because to the special phasor framework. Fluorophores with pH-dependent lifetimes can be used to measure intracellular pH *via* FLIM. After calibration in unidentified structures, these pH probes make it easier to determine pH by shifting their positions on the phasor plot in response to pH variations.^257,258^ The uses of FLIM are further extended when paired with automatic clustering based on the phasor plot^259^ or machine learning methods.^258^

Identifying and detecting cancer cells during tumor progression and metastasis,^260^ automating lysosome segmentation,^261^ and clarifying drug delivery mechanisms by analyzing intracellular trafficking pathways^258^ are just a few of the important applications of using FLIM to measure pH in biological systems. Additionally, FLIM makes it possible to determine ion concentrations. Changes in the fluorescence lifetime of probes sensitive to Ca^2+^ levels can be used to quantify intracellular Ca^2+^ concentrations.^262^ The detection of calcium changes at nanomolar levels and accurate measurements over a wide pH spectrum^262^ are now possible because of advancements in probe development and methodology. FLIM is adaptable to several tissues and cell types, including brain systems, and can assess Ca^2+^ gradients^263^ in epidermal cells when used in conjunction with phasor analysis. Furthermore, fluorescent probes have been created that use lifetime changes to assess the concentration of Na^+.^^264^ For measuring Ca^2+^ and Na^2+^ concentrations and associated subcellular variations, FLIM is therefore a useful instrument.

### 4.5 Molecular interactions: FLIM-FRET

**Fig. 21:**
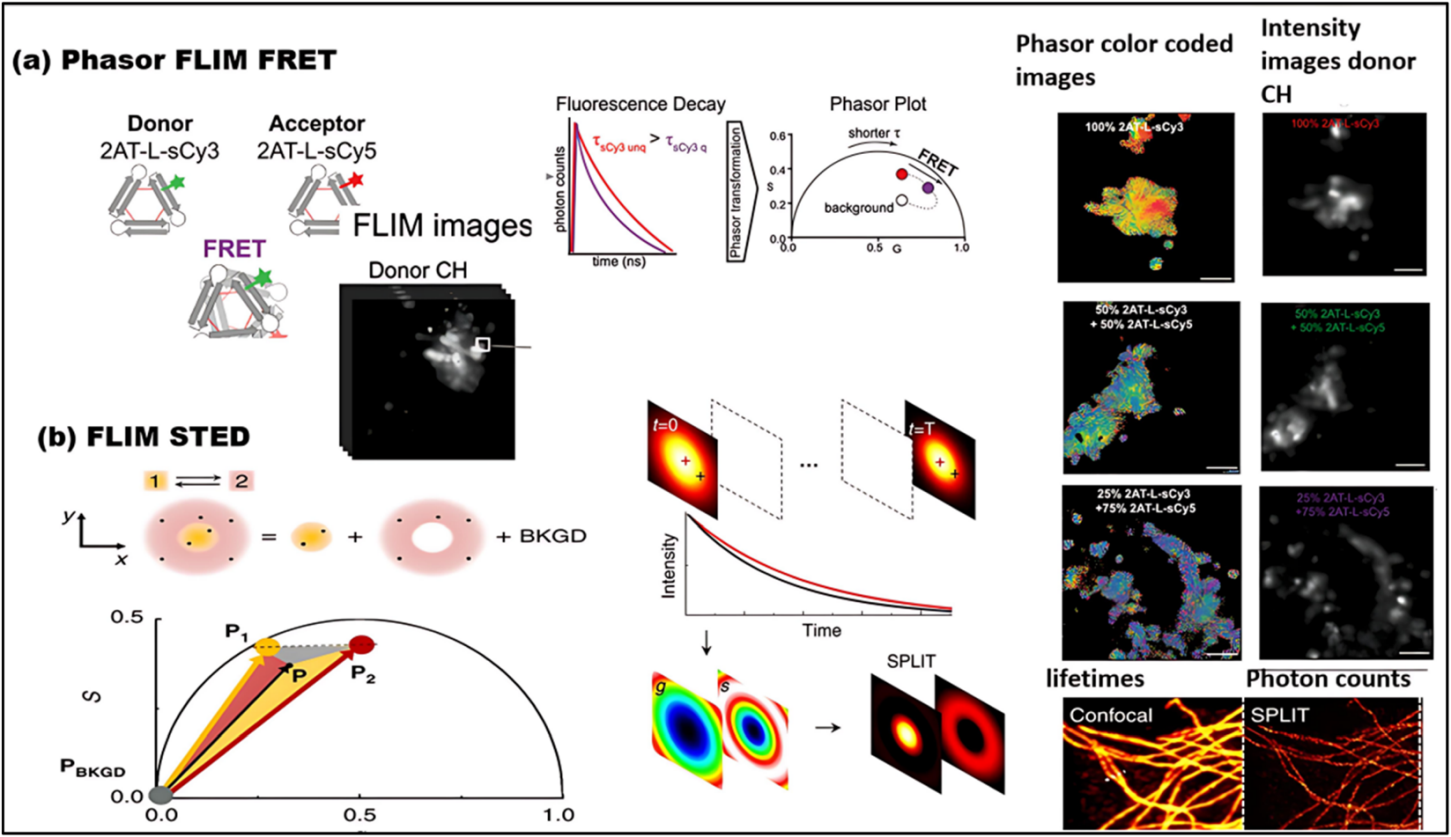
(a) Phasor FLIM-FRET analysis using trimers of Aβ 2AT-*l*-sCy3 (donor) and 2AT-*l*-sCy5 (acceptor) to study oligomeric assemblies in SH-SY5Y cells. Donor quenching is observed in phasor plots at D : A ratios of 0 : 0, 50 : 50, and 25 : 75. Intensity and phasor colour-coded images show FRET occurrences (scale bar, 5 µm). (Adapted with permission from ref. 270 Copyright 2019, American Chemical Society). (b) FLIM-STED imaging using the SPLIT method to enhance spatial resolution by tuning photon lifetimes. Fluorophore lifetime varies based on its position relative to the excitation spot, with shorter lifetimes observed further from the centre. (Adapted with permission from ref. 271 Copyright 2015, Springer Nature Limited).

FFLIM is a useful method for FRET (Fluorescence Resonance Energy Transfer) studies of molecular interactions.^265^ When two molecules are close together (less than 10 nm), excited-state energy is transferred from one fluorescent molecule (the donor) to the other (the acceptor), causing FRET.^266^ Fluorescence quenching as a result of this interaction lowers the fluorescence lifetime of the FRET donor. Lifetime measurements in FLIM are often independent of fluorophore concentration, which has many benefits over intensity-based FRET techniques. Furthermore, because FLIM-FRET only uses donor channel data for analysis, it does away with worries regarding donor spectrum bleed-through and direct stimulation of the acceptor.^267^ By analyzing the phasor shift between the donor-only species and the donor–acceptor complex, FRET is assessed in the phasor plot. The phasors of the autofluorescence background and the unquenched donor for each experiment must be determined in order to compute the FRET efficiency, which can vary from 0% to 100%.^268^ Studies of protein interactions, protein conformational changes, biosensors, chromatin compaction, and other biological applications have made extensive use of FLIM-FRET with phasor analysis.^269^ Using FLIM-FRET and phasors, the oligomerisation of Aβ peptides is evaluated in an *in vitro* trimer model (Fig. 21a).

### 4.6 FLIM-STED for super-resolution

When used in conjunction with STED microscopy, which overcomes the diffraction limit by selectively depleting fluorophores at particular locations within the excitation regions, FLIM improves super-resolution imaging by permitting light emission from only non-silenced fluorophores.^272^ This is accomplished by aligning a STED beam with a Gaussian excitation beam to create a doughnut-shaped focal intensity distribution. The size of the effective fluorescent zone surrounding the center point (or “zero” intensity) determines the subdiffraction spatial resolution. Data gathering with a spatial resolution of less than 40 nm is possible by integrating FLIM with STED.^273^ In multi-color imaging, the STED and FLIM combination has been used for up to five components.^274^ Time-resolved lifespan data can improve STED microscopy resolution without raising the STED beam's peak intensity.^270^ Utilizing the difference in fluorophore lifetime depending on its location within the excitation region, one method is called SPLIT (Separation of Photons by Lifetime Tuning) microscopy (Fig. 21b). The fluorophore's fluorescence lifetime falls as it goes away from the STED beam's zero-intensity point, reaching its minimum close to the doughnut-shaped area where the STED intensity is maximum.^271^ SPLIT significantly improves spatial resolution by analyzing fluorescence lifetime at each pixel using the phasor technique. This SPLIT method is the foundation of the Leica Tau-STED microscope. Furthermore, multicolor imaging has been made possible by combining SPLIT with STED-ISM (STED with Structured Illumination Microscopy).

### 4.7 FLIM for intracellular detection and nanoparticle interaction

Fluorescence Lifetime Imaging Microscopy (FLIM) has been utilized in a limited number of fluorescent-based intracellular applications, primarily involving organic fluorophores and fluorescent proteins.^275^ Some genetically encoded fluorescent sensors, which use fluorescent proteins, have incorporated fluorescence lifetime detection. For example, Tantama *et al.* developed an intracellular pH nanosensor by mutating the red fluorescent protein mKeima, creating pHRed. The pHRed sensor enabled the manipulation of the intracellular pH in live Neuro2A cells for calibration purposes, showing a significant pH-responsive fluorescence lifetime change of approximately 0.4 ns over physiological pH values.^276^ In another study, a genetically encoded FRET-based sensor for detecting ERK activity (EKAR) was introduced, utilizing FLIM for fluorescence lifetime detection. The sensor exhibited changes in fluorescence lifetime indicating ERK activation in HEK293 cells following epidermal growth factor stimulation. This approach was also applied to dendrites and the nucleus of hippocampal pyramidal neurons in brain slices after theta-burst stimulation or trains of back-propagating action potentials. However, the sensitivity of this sensor was limited, with fluorescence lifetime changes of only around 0.06 ns.^277^ To achieve greater sensitivity, Kuimova *et al.* developed a versatile sensor to measure the microviscosity of the environment using a fluorescent indacene derivative as a molecular rotor. The fluorescence quantum yield of the fluorophore increased significantly with the increasing viscosity of the solvent, and the fluorescence lifetime varied from 0.7 to 3.8 ns as viscosity increased from 28 to 950 cP. This molecular rotor was incubated in SK-OV-3 cells, and the resulting fluorescence lifetime map provided a direct measurement of intracellular viscosity.^278^ Despite these advances, the application of FLIM with fluorescent nanoparticles has been underexplored. A few studies have reported the use of fluorescent resonance energy transfer (FRET) interactions with nanoparticles in FLIM detection. For instance, energy transfer between the organic dye DAPI and gold nanoparticles (AuNPs) has been studied using FLIM under two-photon excitation. In the analysis of FLIM images from Madin–Darby canine kidney cells, the fluorescence lifetime of DAPI decreased from 2.5 ns to 0.9 ns due to energy transfer.^279^ Dahan *et al.* pioneered the use of FLIM with QD nanoparticles for imaging fixed cells, demonstrating that QDs could enhance fluorescence biological imaging contrast and sensitivity, notably reducing autofluorescence contributions to the overall image.^280^ Further studies applied FLIM with QDs to detect DNA hybridization events in DNA microarray spots, also showing the potential for lifetime multiplexing using QDs and the Alexa 430 dye with a single excitation-detection readout channel.^281^ Hybrid materials, such as Valerite nanoparticles coated with QDs, were employed for biological applications based on FRET and FLIM. The binding of poly-His-tagged TdTomato protein to the QD surfaces resulted in efficient quenching of QD PL, along with FRET-sensitized emission of the bound TdTomato proteins. These fluorescence lifetime changes were clearly observed in the FLIM images.^282^

## 5. From single probe to multitool: CDs revolutionize FLIM-based bioimaging

Recently, nanoparticles have been explored as fluorophores for FLIM to study cellular systems. When compared to typical organic fluorescent dyes, these NPs provide brighter fluorescence, more photostability, and improved biocompatibility. In addition, their large surface-to-volume ratio allows an extensive variety of sensing techniques. However, the application of FLIM with FNPs remained mostly unexplored. FLIM has been utilized in a limited number of studies for identifying particles in intracellular medium rather than in sensing.^283^ A few examples include employing FLIM to detect intracellular interactions between gold nanoparticles and dyes. Only a few of them demonstrate FLIM with polymer NPs as fluorescent polymeric thermometers for mapping the intracellular temperature and investigating its relevance to organelle functions.^284^

Initially, *N*-acetylcysteine, d-penicillamine, and histidine are used to functionalize CdSe/ZnS quantum dots for intracellular pH detection.^257^ Qds have laso been used for glucose sensing using FLIM.^285^ Semiconductor quantum dots (SQDs) differ from organic fluorophores in PL lifetime. Organic dyes have a simple decay pattern (1–5 ns), making them easy to identify, but they are difficult to distinguish from excitation light and short-lived fluorescence. For effective use of nanoparticles in FLIM within cells, calibration with solutions mimicking the cellular environment is essential, considering salts, proteins, and other cellular components. For intracellular sensing, a different kind of AgInS_2_ (AIS)/ZnS QDs was investigated. The author successfully differentiated between cancerous and non-cancerous cells by examining pH sensing in various subcellular areas, including the cytoplasm and lysosome.^286^ The impact of the protein corona has not yet been taken into account in FLIM imaging. Nandi *et al.* used FLIM-based lifetime imaging to investigate the precise distribution of doxorubicin (DOX) drug release throughout the cell. The authors came to the conclusion that DOX entered the cell cytoplasm more quickly. The observed variations in the drug release pattern were ascribed to variations in protein binding, resulting in different lifetime values in the nucleus and cytoplasm of the cell.^287^

The study emphasized how drug release variation is influenced by protein corona binding affinity, which can be seen in real-time utilizing the FLIM technique. Furthermore, using FLIM imaging, a related study examined the time-dependent DNA fragmentation brought on by PEGylated carbogenic nanodots loaded with DOX.^288^ Beyond DOX, graphene quantum dots in an organized lipid membrane were used to study the translocation of daunomycin, a well-known chemotherapeutic medication. Thus, there is a great deal of promise for improving cellular imaging methods through the combination of FLIM with sophisticated fluorescent probes, especially CDs.^289^ CDs are perfect candidates for FLIM applications because of their remarkable fluorescence characteristics. Furthermore, it is possible to modify the fluorescence of CDs by changing their size, surface chemistry, or functional groups, a crucial feature for time-resolved imaging such as FLIM.

The use of FLIM in conjunction with carbon dots has also shown great promise in FRET tests, in which CDs can act as acceptors or donors.^290^ This makes studying conformational changes, receptor-ligand binding, and protein–protein interactions in living cells easier, providing real-time insights into molecular dynamics. Multiplexed imaging, which allows several targets within a single biological sample to be labeled and examined simultaneously, is one of the most intriguing uses of carbon dots in conjunction with FLIM. This feature greatly expands the breadth of biological discoveries by enabling the analysis of several biomarkers, cellular pathways, and signaling networks in a single experiment without requiring intricate labeling methods. CDs like semiconductor quantum dots, have been investigated for intracellular pH monitoring utilizing the FLIM strategy. FLIM data of CDs were acquired in the presence of different biomolecules like NADH, glutamic acid, arginine, histone, and bovine serum albumin to better simulate the cell environment for intracellular pH imaging. This method can be used for subcellular targeting with fluorescent CDs and pH-based disease progression monitoring. Additionally, FLIM can be used to monitor drug release in various subcellular locations in real-time, which helps improve drug-targeting techniques.^291^ The significance of employing CDs as probes in FLIM for cell imaging is discussed below, emphasizing their potential to offer detailed insights into cellular processes and drug interactions.

### 5.1 FLIM with CDs to study long-live dynamic cellular functionalities

**Fig. 22:**
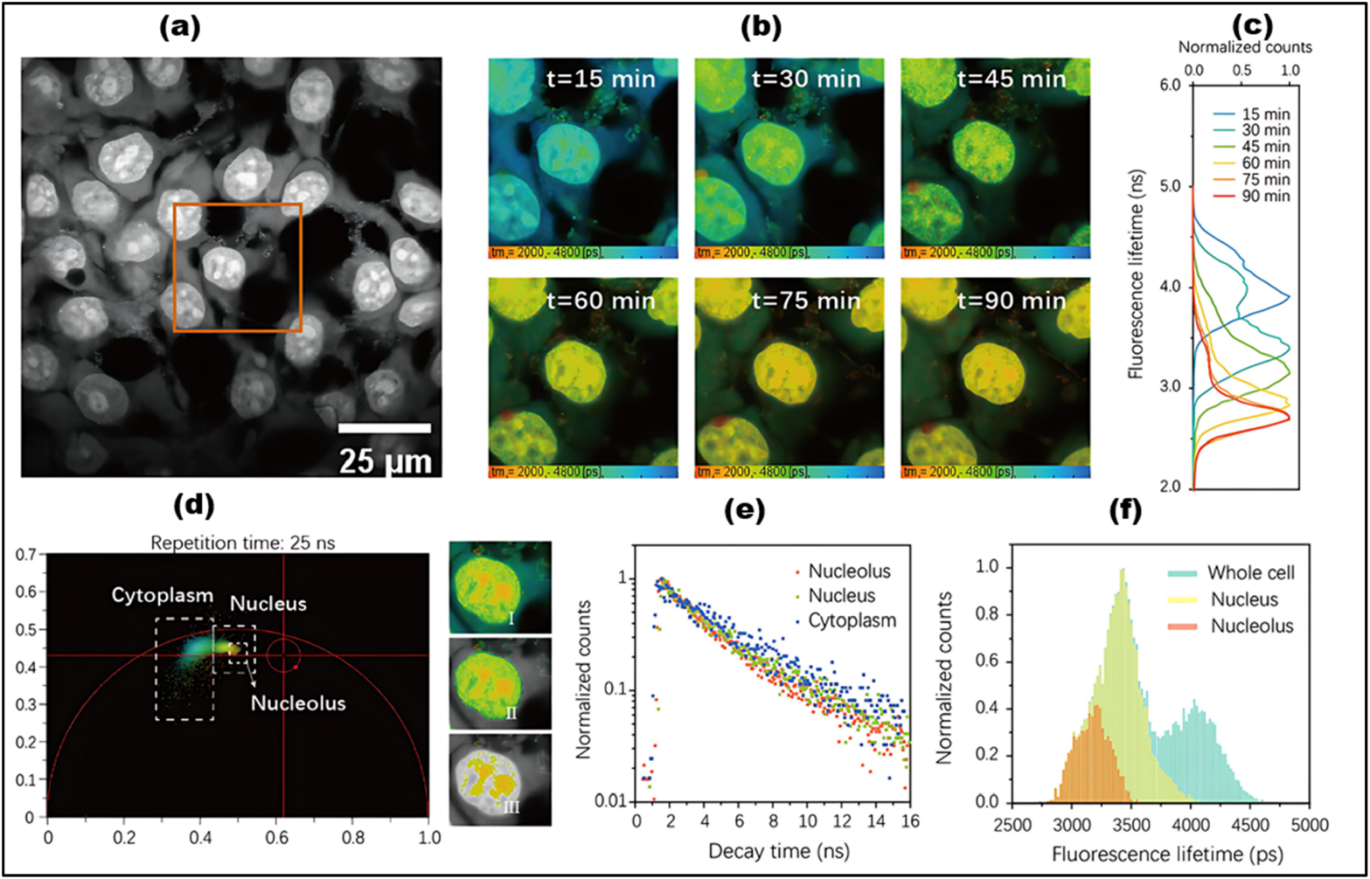
(a) Fluorescence intensity image of CPDs^−3^-stained KYSE-150 cells (*t* = 15 min). (b) *τ* image acquired at 15−90 min after incubating with CPDs^−3^. The color legend of lifetime distribution has been unified (2000−4800 ps). (c) *τ* distribution histogram (d) Phasor plot diagram (*t* = 30 min). Insets: (I) *τ* distribution of the whole cell, (II) *τ* distribution in the selected cluster corresponding to the nucleus region, and (III) *τ* distribution in the selected cluster corresponding to the nucleolus region. The color legend of *τ* distribution has been normalized (2800−4600 ps). (e) Lifetime decay traces of the pixels located in the nucleolus, chromatin, and cytoplasm regions. (f) *τ* distribution histogram of the whole cell (cyan), nucleus (yellow), and nucleolus (orange) regions separated by selecting clusters in the phasor plot image (d) ((a–f) Adapted with permission from ref. 292. Copyright 2021 American Chemical Society).

In real-time, STED and FLIM optical imaging methods may observe sub-cellular targets at high resolution. Fluorescent probes limit practical imaging, especially long-term live-cell nucleic acid imaging. Current probes lack targeted specificity, biocompatibility, low excitation power, and photostability. To fully utilize the potential of these advanced imaging methods, we need to find solutions to the limitations posed by probes. In their study, Liu, Song, *et al.* produced CDs that can be used for ferric ion sensing and cellular imaging purposes, irrespective of the excitation source. For this study, researchers carefully designed and created CPDs-3, a tiny CPD material that produces light with outstanding efficacy and stability. This substance is used to image nucleic acids in living cells, allowing for fluorescence lifetime study and super-resolution imaging. The nanoprobe demonstrated remarkable emission properties when evaluating nucleic acid interactions, greatly improving the signal-to-noise ratio in both the spatial and temporal domains. Direct imaging of chromatin structures with sub-diffraction resolution (90 nm) is made possible by CPDs-3, which has a low saturation intensity of 0.68 miliwatt (mW) 0.23 megawatt (MW cm^−2^) and requires little excitation <1 microwatt, (µW) and depletion power (<5 mW). Long-term imaging in both STED and FLIM setups is possible with CPDs-3 because of its exceptional photostability, high photonic efficiency, and low toxicity. This illustrates its great potential for examining the long-term actions of nucleic acids. Important information on CPDs^−3^'s interactions with different nucleic acid structures inside cells can be gained by tracking its fluorescence lifetime over time. In (Fig. 22a), the fluorescence lifespan of CPDs^−3^ was investigated in living cells following incubation for various intervals of time. CPDs^−3^ had a broad lifetime range of 3500 to 5000 picoseconds (ps) within the first 15 min, indicating that it was binding free nucleic acids, as illustrated in Fig. 22b and c. The lifetime distribution altered throughout time and was separated into two components, one for the cytoplasm and one for the nucleus. This suggests that CPDs^−3^ aggregates and binds differently to nucleic acids investigated in living cells following incubation for various intervals of time. CPDs^−3^ had a broad lifetime range of 3500 to 5000 ps within the first 15 min, indicating that it was binding free nucleic acids, as illustrated in Fig. 22d–f. This demonstrates CPDs^−3^'s enormous promise for future dynamic, long-term investigations of nucleic acid functions.^292^

### 5.2 FLIM and CDs for multicolor imaging-based selective interaction to detect Gram-negative bacteria

**Fig. 23:**
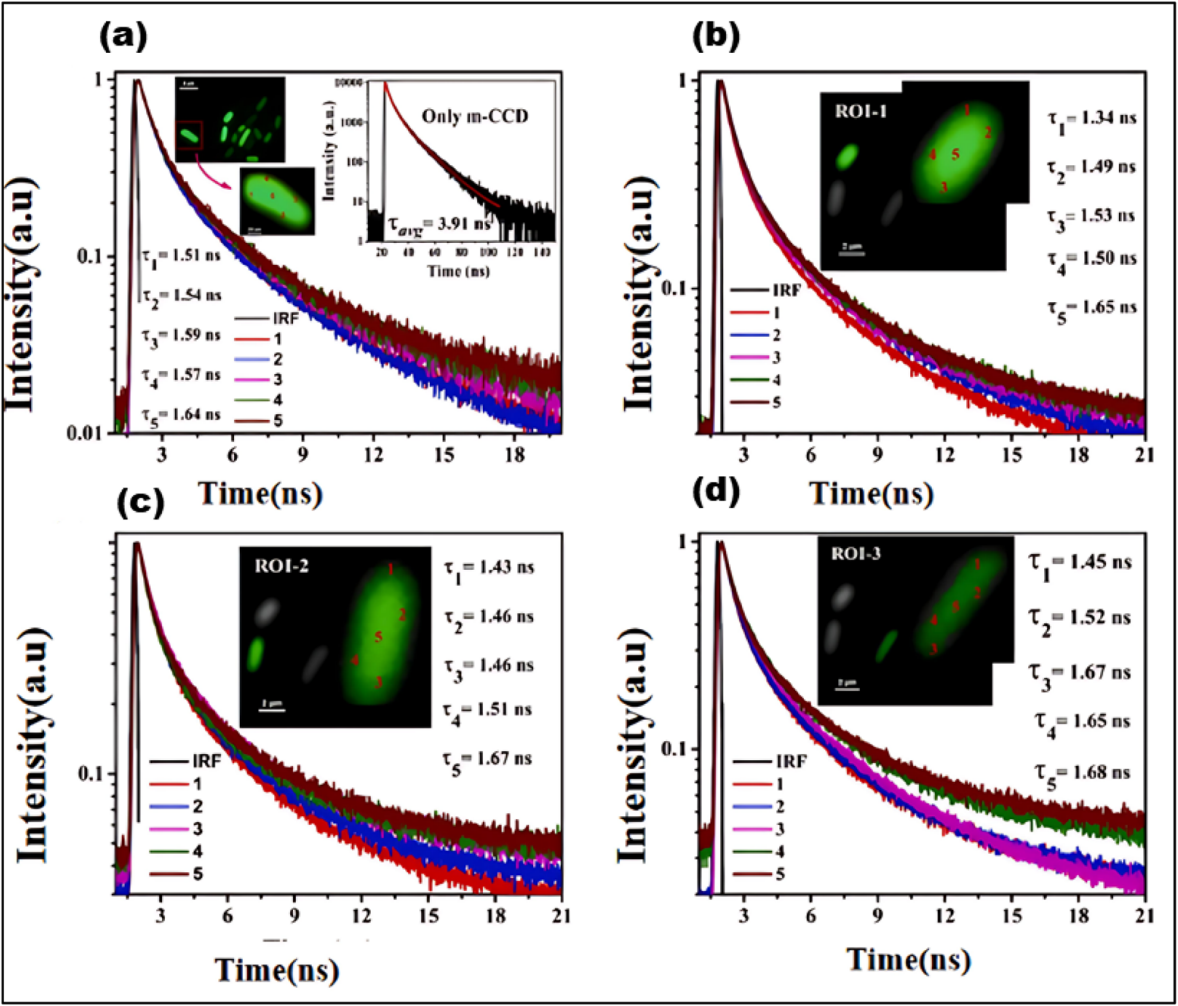
(a) FLIM-based point lifetime decay curves of individual *m*-CCD treated *E. coli* measured on the cell wall (1, 2, 3 & 4) and cytoplasm; FLIM-based point lifetime decay at different ROI (triplicates) for data reproducibility (b) ROI^−1^; (c) ROI^−2^ (d) ROI^−3^ ((a–d) Adapted with permission from ref. 293 Copyrights 2023 Elsevier B.V).

The emergence of antibiotic-resistant Gram-negative bacteria poses a significant challenge to healthcare, necessitating the creation of innovative and efficient detection methods. Pathak, Navaneeth, and coworkers have presented a straightforward and effective technique for recognizing Gram-negative bacteria in particular. They used fluorescence lifetime (FLT) response in conjunction with bioimaging for detection, and they used multi-emissive colistin-passivated carbon dots (*m*-CCDs). A one-step hydrothermal procedure with microwave assistance was used to create the *m*-CCDs. The time-resolved fluorescence properties of the *m*-CCDs following attachment to the bacteria were evaluated using FLIM and Confocal laser scanning microscopy (CLSM) in order to examine their interaction with Gram-negative bacteria. When *m*-CCDs interact with *E. coli* bacteria, the bacterial cells produce fluorescence signals. Gram-negative bacteria like *E. coli* can be particularly interacted with by *m*-CCDs through its colistin moiety. When *m*-CCDs interact with the outer membrane of *E. coli*, colistin disrupts the lipopolysaccharide by displacing certain ions, resulting in the formation of membrane pores. These pores then enable *m*-CCDs to penetrate the bacterial cell. The FLT decay curves of *E. coli* cells treated with *m*-CCDs, as determined by the researchers, are shown in Fig. 23a. The average lifetime of *m*-CCDs-treated *E. coli* ranged from 1.51 to 1.64 ns at different points on the bacterial cell, as indicated in Table 4. This has a much shorter *τ* compared to the typical duration of free *m*-CCDs, which was approximately 3.91 ns. It appears that the interaction between *m*-CCDs and *E. coli* leads to an increase in non-radiative processes, which in turn reduces the fluorescence lifetime. Results from the FLT measurements were consistent across the *E. coli* cell's several regions of interest (ROI). By conducting FLT measurements at three different ROIs and gathering triplicate data from five different sites, the average fluorescence lifespan (τavg) of *m*-CCD-bac was confirmed (Fig. 23b–d). Additionally, there was no discernible difference in the FLT profile between the bacterial cell's cytoplasm and outer membrane. The average lifespan for *m*-CCD-bac was found to be almost constant throughout all chosen areas, ranging from roughly 1.34 to 1.68 ns.^293^

**Table 4:** The average lifetime of *m*-CCD-treated *E* coli Bacterias. Adapted with permission from ref. 293. Copyrights 2023 Elsevier B.V

Data point	*A*_1_	Err ±	*A*_2_	Err ±	*A*_3_	Err ±	*τ*_1_ [ns]	Err ±	*τ*_2_ [ ns]	Err ±	*τ*_3_ [ ns]	Err ±	*τ*_avg_ [ ns]	*x* ^2^
1	0.1	0.005	0.64	0.032	0.26	0.013	6.07	0.3035	0.56	0.028	2.07	0.103	1.51	1.06
2	0.09	0.0045	0.65	0.0325	0.26	0.013	6.3	0.315	0.59	0.029	2.24	0.112	1.54	1.03
3	0.1	0.005	0.63	0.0315	0.27	0.0135	6.3	0.315	0.59	0.029	2.17	0.108	1.59	1.02
4	0.1	0.005	0.63	0.0315	0.27	0.0135	6.1	0.305	0.58	0.029	22	0.11	1.57	1.03
5	0.1	0.005	0.63	0.0315	0.27	0.0135	6.4	0.32	0.6	0.03	2.3	0.115	1.64	1.04
*m*-CCD	0.13	0.0065	0.49	0.0245	0.38	0.019	13.44	0.672	0.91	0.045	4.53	0.226	3.91	1.05

### 5.3 FLIM with CDs for monitoring cell cycle

**Fig. 24:**
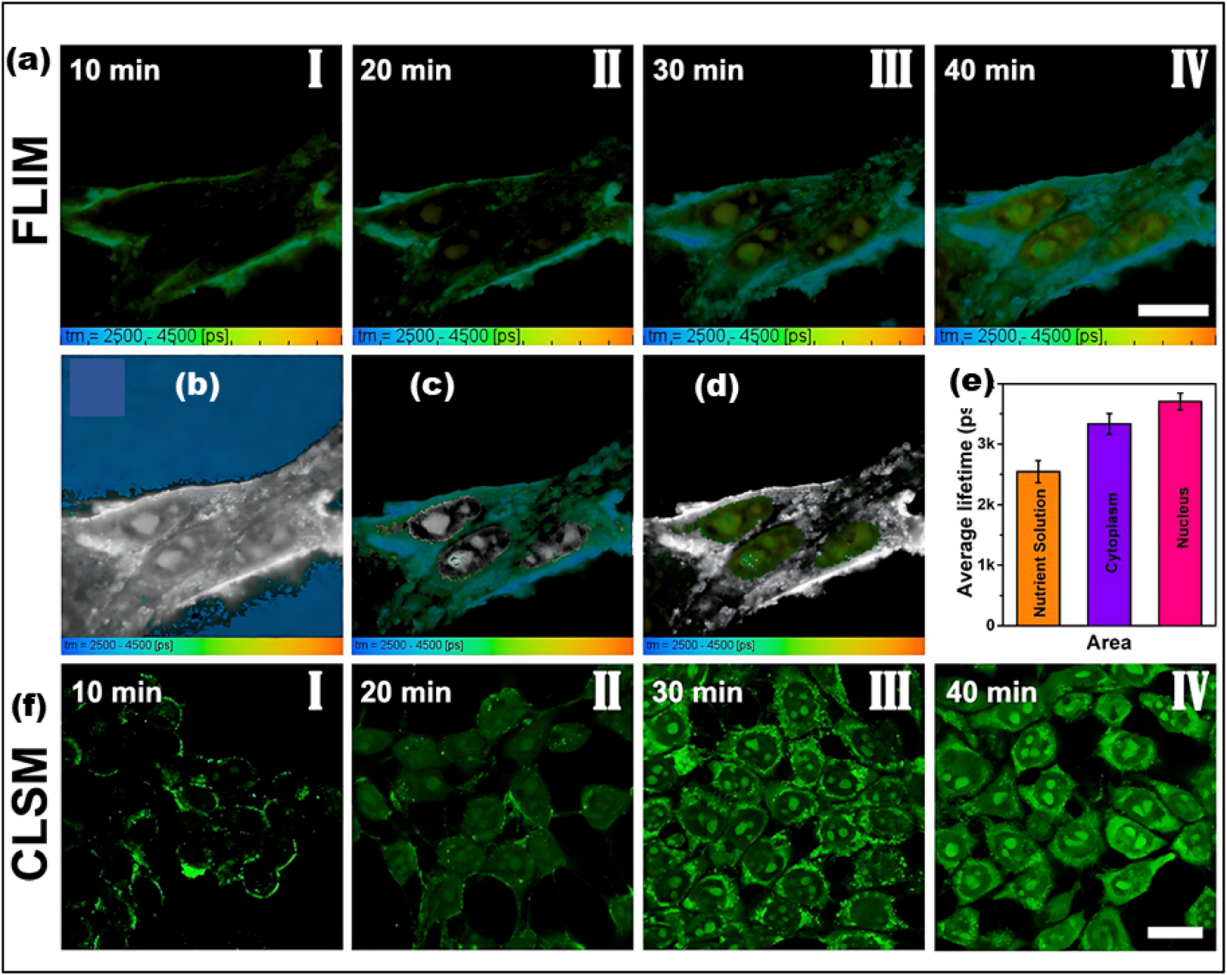
(a) After adding FNCDs, the FLIM images of 4T1 cells under different times. (b) The FLIM images of FNCDs in the nutrient solution. (c) The FLIM images of FNCDs in the cytoplasm. (d) The FLIM images of FNCDs in the nucleus. (e) The average lifetime of FNCDs in the nutrient solution, cytoplasm, and nucleus, respectively. (f) After adding FNCDs, the CLSM images of 4T1 cells under different times. Scale bar: 20 µm. ((a–f) Adapted with permission from ref. 294 Copyrights 2020 Elsevier B.V).

Drugs have an impact on the various stages of cellular development. In a recent study, Li, *et al.* conducted research on FNCDs to investigate their potential as cell-targeting probes and their ability to deliver drugs at various stages of the cell cycle. Through a one-step hydrothermal treatment of glycine and 2,4-difluorobenzoic acid, a new probe was successfully produced. The FNCDs showed a remarkable QY 57%, along with excellent optical stability. Furthermore, they exhibited the capacity to sustain their fluorescence despite variations in circumstances such as ionic strength, pH, temperature, and the presence of guest molecules. FLIM was utilized to observe the entry of FNCDs into cells and their subsequent arrival at the nucleolus. Through time-dependent experiments, it was observed that FNCDs were able to enter the cytoplasm of 4T1 cells *via* endocytosis within 10 min of co-incubation. Furthermore, after 20 min, the FNCDs were found to have reached the nucleolus. Fig. 24 offers a thorough summary of the FLIM data. Fig. 24a illustrates the dynamic uptake and localization of FNCDs in 4T1 cells over 40 min, with images taken at 5-min intervals. The FLIM images demonstrate the process of FNCDs being absorbed into the cytoplasm and nucleus from the nutrient solution. Fig. 24b–d shows the FLIM images of FNCDs in the nutrient solution, cytoplasm, and nucleus, respectively. These images reveal different fluorescence lifetimes in each environment. The measured lifetimes varied across different parts of the cell, 2545.35 ps in the nutrient solution, 3334.04 ps in the cytoplasm, and 3702.49 ps in the nucleus. Fig. 24e provides a quantification of the average fluorescence lifetimes, highlighting how FNCDs are influenced by their surroundings. Fig. 24f provides additional support to these findings through the use of CLSM images, which visually confirm the spatial distribution of FNCDs within the cells. This experiment has revealed a groundbreaking discovery FNCDs have the remarkable ability to penetrate cells, target specific areas within them, and exhibit stable fluorescence lifetimes that respond to the cellular environment. This establishes FNCDs as powerful fluorescent probes for live cell imaging.^294^

### 5.4 FLIM-based CDs photo catalyst for ROS-mediated inhibition of algae growth

**Fig. 25:**
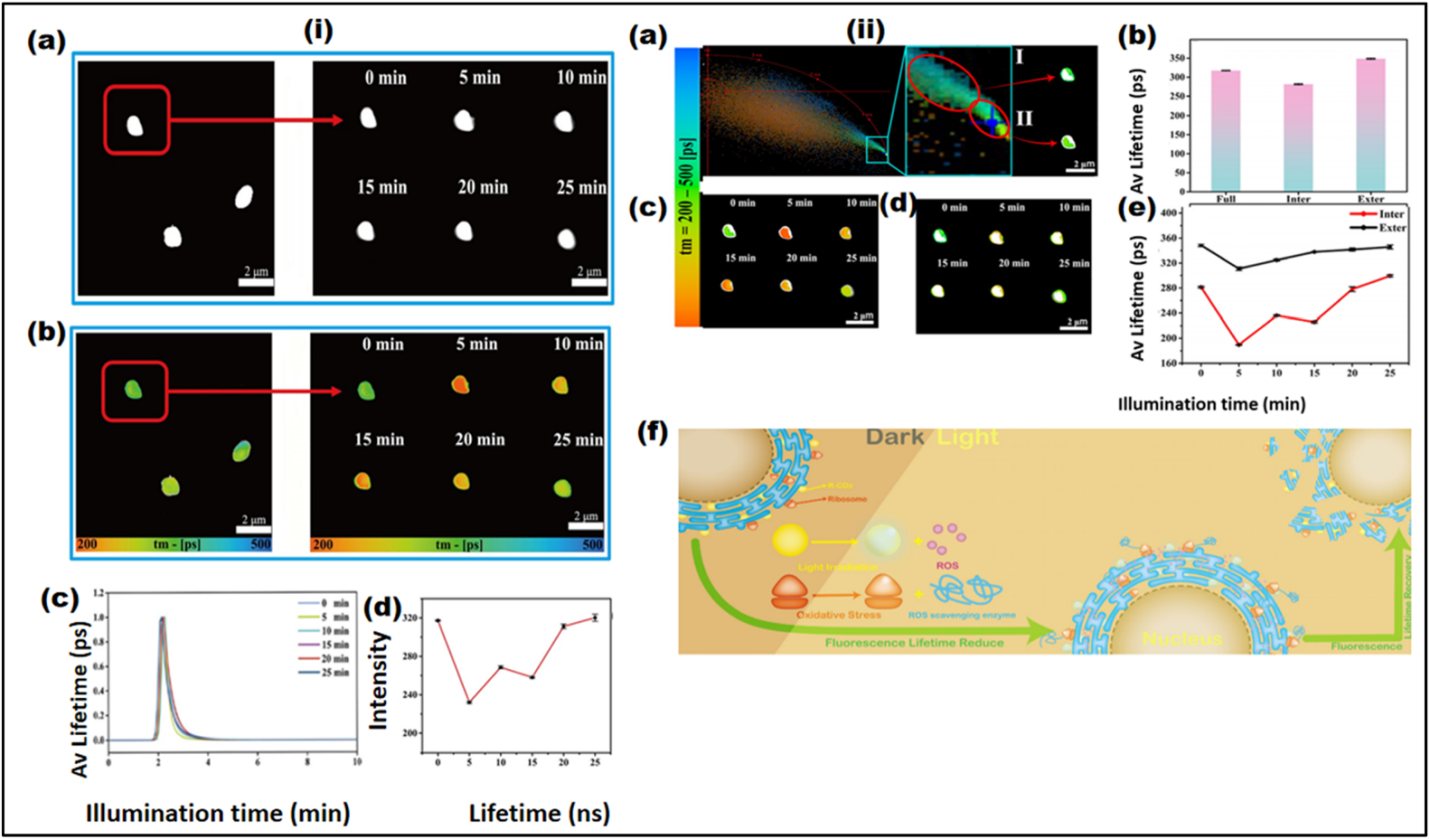
(i) (a) FLIM intensity map of the PGS@R-CD system over time. (b) FLIM lifetime map of the PGS@R-C system over time. (c) Line graph showing the average lifetime changes of PGS@R-CD cells over time. (d) Curve fitting of the lifetime changes of PGS@R-CD cells over time ((a–f) Adapted with permission from ref. 295 Copyrights 2023 by the authors. Licensee MDPI, Basel, Switzerland).

Human-caused potentially dangerous algal blooms have grown more prevalent, highlighting the significance of taking effective elimination measures rapidly. Researchers in this study have managed to successfully generate R-CDs that are safe and stable for biological usage. When exposed to sunlight, these small particles emit ROS unusually. In addition, FLIM was first used to explore the physiological responses related to oxidative stress caused by Phaeocystis globosa Scherffe (PGS) in response to ROS threat. The FLIM image (Fig. 25i(b)) provides an extensive look of the cell's fluorescence lifespan and structure compared to the CLSM image (Fig. 25i(a)), which records fewer details. In the absence of light, the fluorescence lifetime stayed constant at an average of 319.2 ps. Nevertheless, there was a was a noticeable difference between central and peripheral regions of the cell. The average fluorescence lifetime significantly decreased following light exposure, suggesting changes in the physiological roles of the PGS cells' subcellular structures (Fig. 25i(c)). The fluorescence lifespan first decreased markedly as the light duration increased, but it eventually recovered to its initial level. These alterations imply that significant changes in the physiological state of the PGS cells were brought about by reactive ROS produced by R-CDs when exposed to light. The shape of the fluorescence lifetime fitting curve Fig. 25i(d) shows that the R-CDs stayed stable and only functioned as catalysts. R-CDs connect to the PGS cells' internal (II) and external (I) subcellular structures, as shown by the phase diagram in (Fig. 25ii(a)). Fig. 25ii(b) illustrates the differences between the exterior and internal components of PGS cells and R-CDs. Fig. 25ii(c and d) illustrates how the R-CDs produced ROS in response to light exposure, resulting in oxidative stress. Ribosomes in the endoplasmic reticulum released ROS scavengers, which had a major impact on the R-CDs' fluorescence lifetime. As depicted in Fig. 25ii(e and f), the heightened presence of ROS scavengers in close contact to the endoplasmic reticulum (ER) led to a notable enhancement in longevity. A glimpse into the interesting world of R-CDs and their powerful anti-algae mechanism.^295^

### 5.5 FLIM integrated with CDs to mimic complex microenvironments inside the cells

**Fig. 26:**
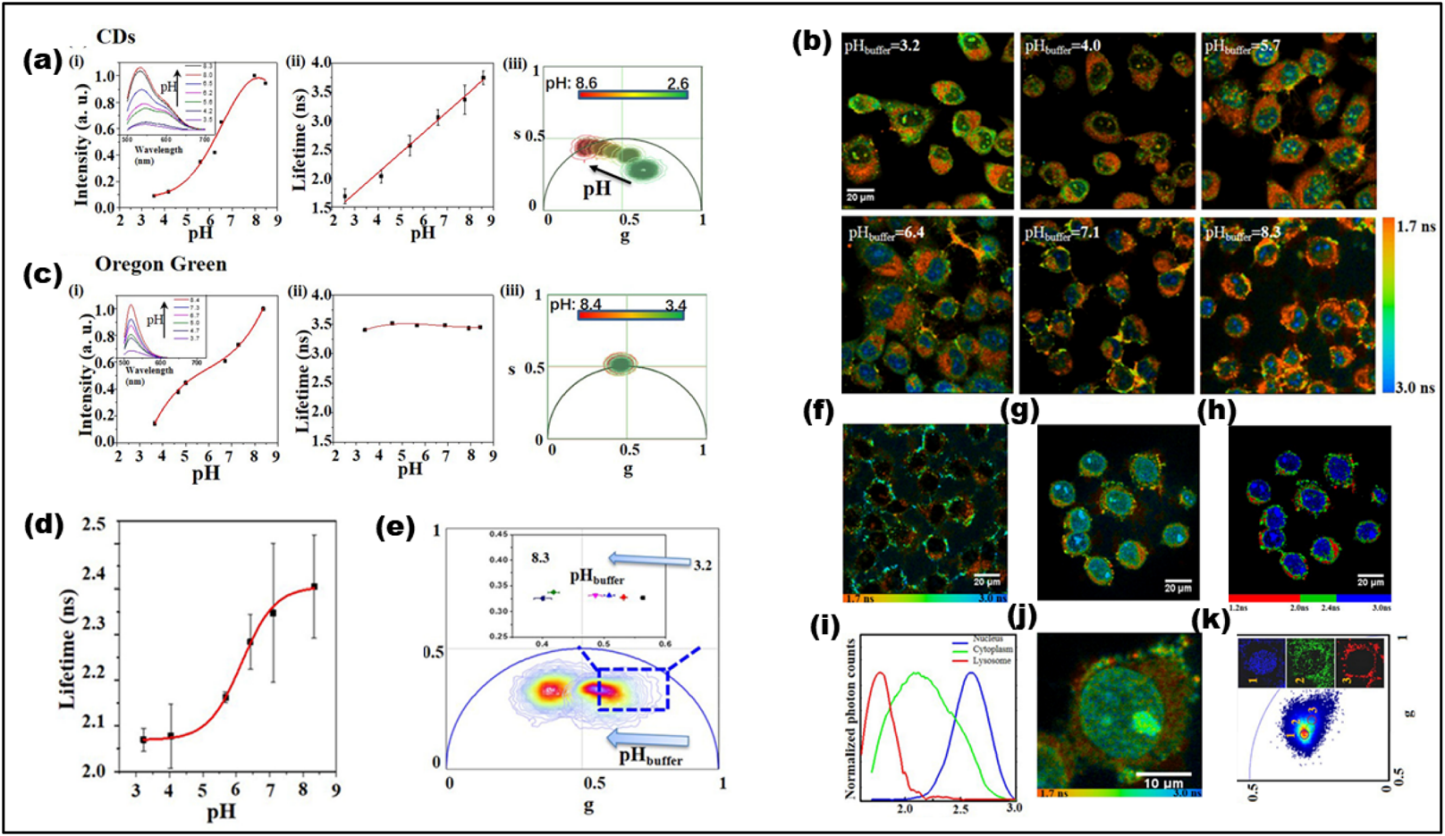
Comparison of the fluorescence characteristics of (a) CDs and (b) commercial pH sensor oregon green in different pH environments. (i) Fluorescence intensity changes with pH values (the error bar is very small in this case and the inset is the fluorescence spectra), (ii) fluorescence lifetime changes with pH values, and (iii) phasor analysis of FLIM images. pH response of CDs in HeLa living cells. (c) Fluorescence lifetime images of cells labeled with CDs at different pH buffers, scale bar: 20 µm. (d) The relationship between the average fluorescence lifetime and the pH values of the FLIM images in (c). (e) A phasor diagram obtained using the phasor analysis of the FLIM images in (c), in which the enlarged part of the phasor diagram gives the maximum density of the corresponding phasor plot for each pH value. The FLIM images of the CDs in the HeLa cells were compared. (f) Cells were incubated with CDs for 2 hour. (g) Cells were incubated with CDs for 2 hours, and then cultured with digitonin for 3 min for cell permeabilization (scale bar: 20 µm). (h) The different fluorescence lifetimes of CDs in the lysosomes, cytoplasm, and nucleus of the cell are in discrete colors. (i) Statistical curves of the CDs fluorescence lifetime distribution in different regions of the cell (lysosomes, cytoplasm, and nucleus). (j) FLIM images of a permeabilized cell and (k) the corresponding phasor FLIM distribution, where 1, 2, and 3 correspond to the three regions of the nucleus, cytoplasm, and lysosomes, respectively. (a–k) Adapted with permission from ref. 296. Copyright 2020, MDPI (Open access).

Understanding intracellular movement and functions requires careful monitoring of intracellular pH. There are several limitations when it comes to biosensing using fluorescence intensity or spectra analysis. For the first time, FLIM was used to detect intracellular pH using pH-sensitive CDs. Prior to investigating CD fluorescence in cells, control cells' inherent fluorescence was contrasted with that of CDs-treated cells. The study revealed that the intensity of cell auto-fluorescence was significantly lower compared to the fluorescence of CD's, with a difference of 8.5 times. Additionally, the auto-fluorescence lifetime was measured to be approximately 0.98 ns. Therefore, the autofluorescence signal has no impact on the FLIM measurement of cells treated with CDs. As depicted in Fig. 26c, the intracellular pH of the HeLa cells was altered when treated with various pH buffers. Notably, variation in pH were observed across different regions of the cells. Consequently, the FLIM images of CDs inside living cells showed a wide range of colors in various locations. As the buffer's pH was elevated, the cells' fluorescence lifespan gradually increased (Fig. 26d). The average fluorescence lifespan of the CDs inside the cells progressively increased from 2.1 ns to 2.4 ns across the whole pH value range that was examined. The curve adopted a sigmoidal shape in contrast to the smooth increase seen in Fig. 26a(ii), presumably as a result of differences in the intracellular pH in relation to the pH of the buffer solution. An interesting pattern was found when examining the phasor FLIM data (Fig. 26e): the intracellular CDs data moved to the left as the pH level increased. Fig. 26a(iii) illustrates this tendency, which implies that the pH response of CDs in living cells is similar to that seen in solution. The phasor FLIM behavior of the CDs in solution is in agreement with this result. A little percentage of CDs penetrate the nucleus, but the majority are found in the cytoplasm. Given the rapid division of the HeLa cell, the ratio of cytoplasm to nucleus would be lower, resulting in relatively larger nuclear size compared to normal cells. Therefore, there is a high likelihood of external material entering the nucleus when the cell membrane is disrupted. It is possible that this could lead to the distribution of CDs within nuclei. Furthermore, as illustrated in Fig. 26c, the pH buffer affected the cell membrane's permeability, which resulted in a greater uptake of CDs into the cells and nuclei. Digitonin treatment improved the permeability of the membrane and nuclear envelope, enabling a more accurate examination of the CDs' fluorescence lifespan inside the nucleus. The FLIM pictures of CDs in living cells before and after digitonin treatment are contrasted in (Fig. 26f). The CDs seldom ever entered the nucleus in (Fig. 26f) when the cell membrane was intact, and their fluorescence lifespan varied between 1.6 and 2.3 ns. Nevertheless, the CDs were able to enter the nucleus when the membrane permeability was changed, leading to a fluorescence lifespan of roughly 2.5–2.8 ns, which was longer than in the cytoplasm (Fig. 26g). Various colors are used to illustrate the various fluorescence lifetimes of CDs in the cytoplasm (green), lysosomes (red), and nucleus (blue) in (Fig. 26h and i). The FLIM picture of CDs inside a HeLa cell treated with digitonin is displayed in Fig. 26j, and the matching phasor image analysis is shown in Fig. 26k. Three separate zones with varying distributions are shown by the data in Fig. 26k: the nucleus, neutral cytoplasm, and acidic lysosomes.^296^

The CDs in this work were able to penetrate the nuclear envelope, which allowed for the investigation of the microenvironment inside the nucleus, in contrast to the majority of CDs or SQDs, which have a restricted ability to reach the nucleus.^297^ This study offers a useful method for precisely measuring intracellular pH in intricate biological systems by fusing FLIM technology with pH-sensitive CDs.

### 5.6 Unaltered imaging capability of CQDs in cellular environments

**Fig. 27:**
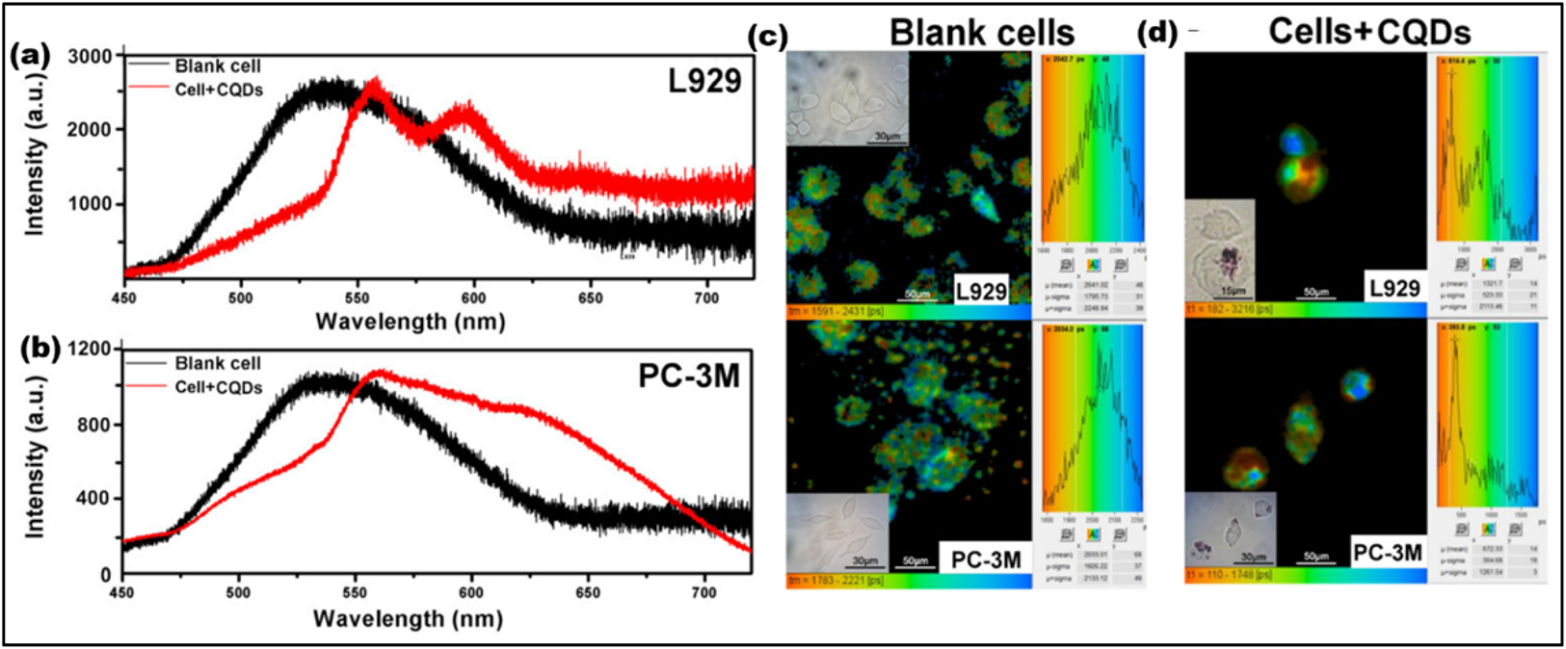
Comparison of fluorescence intensity with and without CQDs for 48 h at an excitation wavelength of 400 nm in (a) L929 cells and (b) PC-3M cells. Comparison of FLIM images of L929 and PC-3M cells (c) with and (d) without CQDs. The excitation wavelength was 400 nm, and the emission wavelength was 560 nm. Insets: Bright-field images of L929 and PC-3M cells. ((a–d) Adapted with permission from ref. 29 Copyrights 2020, International Journal of Nanomedicine).

Understanding the biocompatibility and biointeractions of nano-CQDs is essential for evaluating their potential risks to human health, particularly for biomedical applications such as imaging and therapy.^54^ In a study, the biocompatibility and biointeractions of CQDs synthesized *via* laser ablation in liquid were assessed for their potential use as optical nanoprobes. The CQDs were evaluated through a variety of *in vitro* and *in vivo* assays to determine their cytotoxicity, apoptosis, biodistribution, immunotoxicity, and gene expression alterations. For *in vitro* analysis, L929 and PC-3M cells were incubated with CQDs (1 µM) for 48 hours, followed by examination using fluorescence lifetime imaging microscopy (FLIM) with 400-nm excitation. Control experiments were performed with blank cells (without CQDs) to evaluate autofluorescence. Notably, cells demonstrated strong autofluorescence under UV excitation in the range of 260 to 400 nm, which may interfere with CQD labeling applications. Following this, lifetime images were captured at 560 nm, based on established research protocols. The results of FLIM revealed that the average time molecules remained in the excited state followed a mono-exponential function for both L929 and PC-3M cells (Fig. 27a and b). Autofluorescence lifetime distributions for L929 (1591–2431 ps) and PC-3M cells (1783–2221 ps) were observed prior to CQD uptake (Fig. 27c and d). After CQD uptake, these distributions shifted to 182–3216 ps for L929 cells and 110–1748 ps for PC-3M cells, with a notable increase in the average excited-state lifetime in L929 cells and a decrease in PC-3M cells. The average fluorescence lifetime of CQDs inside the cells was longer (0.94 ns) compared to when they were measured independently, indicating the cellular uptake of CQDs altered their optical properties.^298^

### 5.7 Target specific CDs flim probe to study nucleus

**Fig. 28:**
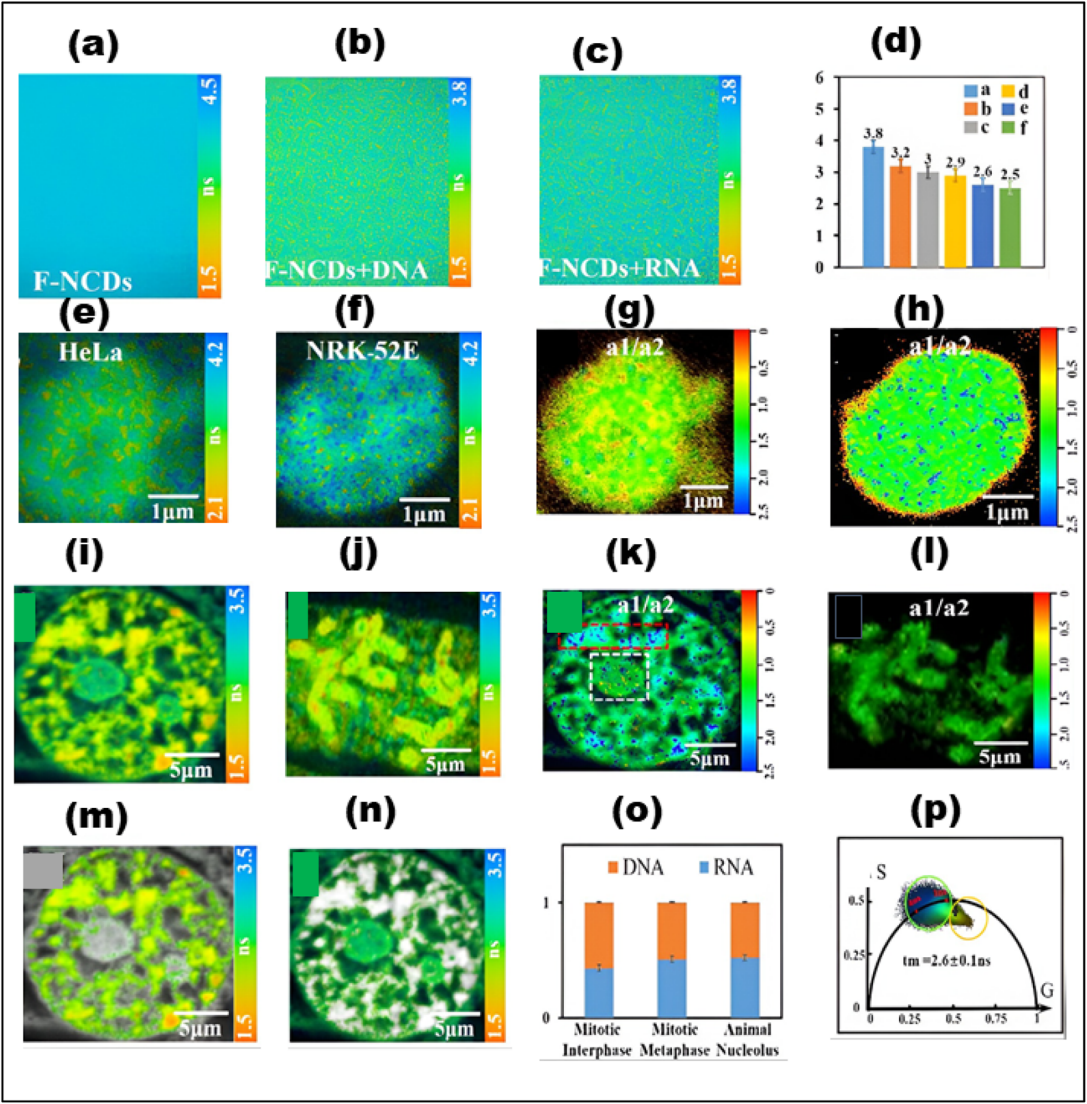
(a–c) FLIM imaging of FNCDs in pure solution, FNCDs bound to DNA, and F-NCDs bound to RNA in mixed solution. (d) Fluorescence lifetime change of F-NCDs binding to different concentrations of DNA and RNA. (e and f) FLIM images of nucleolus in HeLa and NRK-52E cells stained with F-NCDs. Co-localization results of F-NCDs with the commercial dye Hoechst 33 342 showed that F-NCDs targeted the nucleolus of HeLa and NRK-52E cells). (g and h) a1/a2 ratio of (e and f), respectively. Color-coded by the a1/a2 ratio. (i and j) FLIM image of a single plant cell stained with F-NCDs in interphase and metaphase (k and l) a1/a2 ratio of (i and j). (m and n) FLIM images of DNA and RNA separated by a phasor plot. (o) Comparison of DNA and RNA ratios in different species and mitotic stages. (p) Phasor plot corresponding to (i). All a1/a2 ratio images were denoised. ((a–p) Adapted with permission from ref. 299 Copyrights American Chemical Society, 2025.

Moreover another study was conducted for fluorine-nitrogen codoped carbon dots (F-NCDs) in conjunction with two-photon fluorescence lifetime imaging microscopy (TP-FLIM) for the *in vivo* visualization and imaging of nucleic acids, specifically DNA and RNA. The FNCDs were synthesized *via* a hydrothermal method and showed excellent biocompatibility, with an ability to penetrate cell membranes easily and localize in the nucleus, where nucleic acids are abundant. Fluorescence lifetime measurements were performed on pure FNCDs, as well as FNCDs bound to DNA and RNA in mixed solutions. The average fluorescence lifetimes of the FNCDs were measured as follows: 3.8 ± 0.1 ns for pure FNCDs, 2.5 ± 0.2 ns for FNCDs bound to DNA, and 3.2 ± 0.2 ns for F-NCDs bound to RNA (Fig. 28a–c). These results highlight the sensitivity of FNCDs to nucleic acid binding, with noticeable fluorescence lifetime changes when they were bound to DNA and RNA. This allowed for the differentiation between the two types of nucleic acids based on their fluorescence lifetime properties. Moreover, the fluorescence lifetime of FNCDs was observed to decrease significantly upon binding to DNA and RNA, which suggests that these carbon dots can distinguish between nucleic acids based on their fluorescence lifetime properties (Fig. 28d). The changes in fluorescence lifetime were studied in detail in HeLa and NRK-2E cells, where TP-FLIM revealed the distribution of DNA and RNA within the nucleolus. The fluorescence lifetime images (Fig. 28e and f) showed average lifetimes of 3.0 ± 0.1 ns for HeLa cells and 3.2 ± 0.1 ns for NRK-2E cells, reflecting the differences in DNA and RNA concentrations in the nucleolus of these two cell types. The amplitude ratio (a1/a2) was calculated to reflect the DNA/RNA concentration ratio in the nucleolus (Fig. 28g and h), revealing a ratio of 0.95 in HeLa cells and 0.90 in NRK-2E cells, further demonstrating distinct DNA/RNA distributions between the two cell types. FNCDs were also used for imaging plant cells, such as onion root tip meristematic cells, during various stages of mitosis (Fig. 28i–l). The fluorescence lifetime data of plant cells showed dynamic changes in the DNA/RNA ratio during the cell cycle (Fig. 28m and n). In interphase, the average DNA/RNA ratio was 1.35, with the nucleolus showing a ratio of 0.92. However, during metaphase, the ratio decreased to 0.98, with RNA showing a more pronounced increase relative to DNA. In metaphase, the average DNA/RNA ratio across the entire plant cell nucleus decreases to 0.98 (Fig. 28o). This indicated dynamic fluctuations in DNA and RNA concentrations as cells transitioned through the cell cycle. The TP-FLIM data from plant cell nuclei were further analyzed using a phasor plot (Fig. 28p), which visually distinguished the DNA and RNA regions based on their fluorescence lifetime distributions. This provided a comprehensive view of DNA/RNA distribution within plant cells, exemplifying the utility of F-NCDs in plant cell imaging.^299^

### 5.8 FLIM -CDS as pH sensors in living cells

**Fig. 29:**
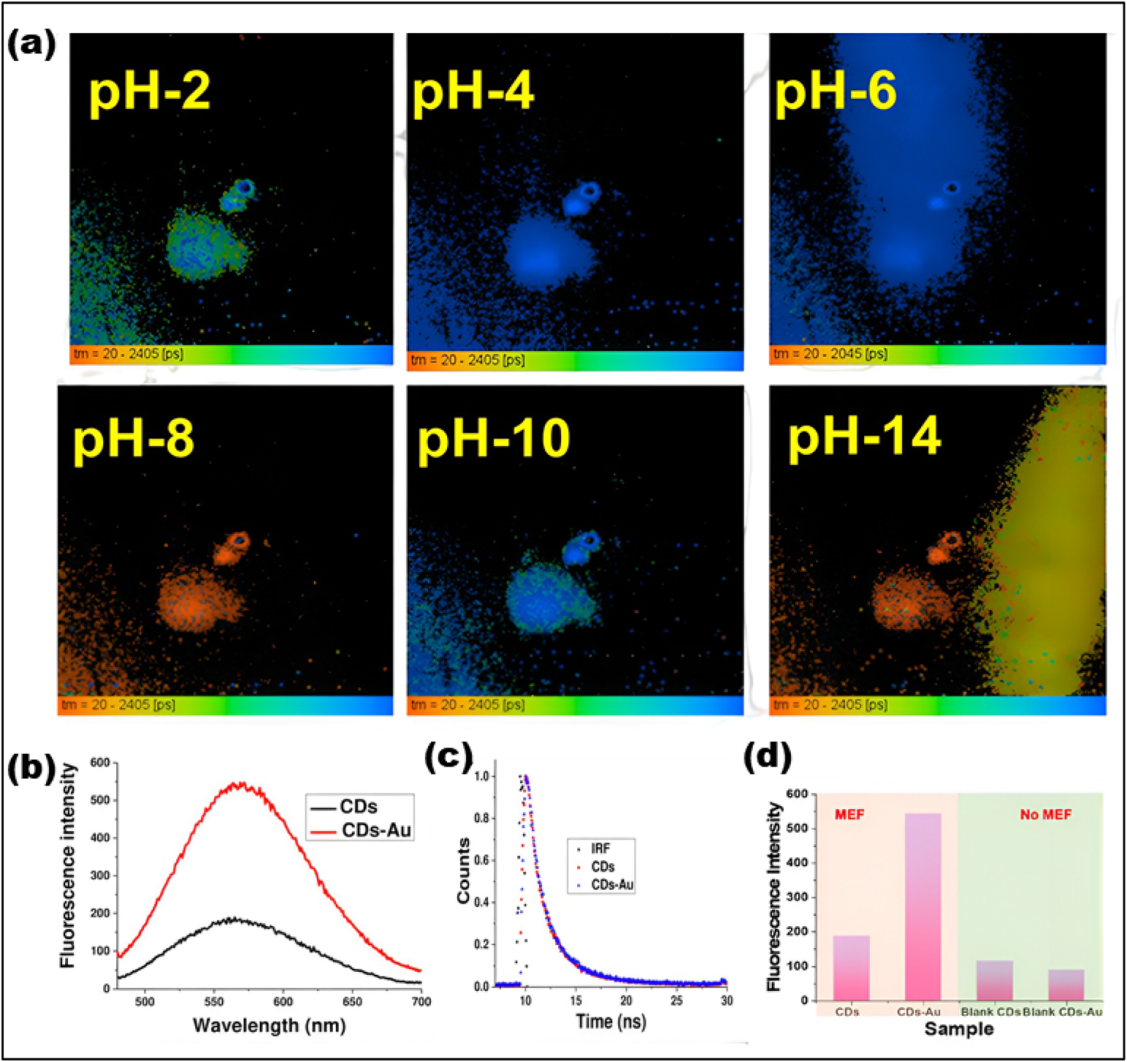
FLIM images of CDs at different pH levels. (b) Fluorescence intensity and (c) time-resolved fluorescence decay curves of CDs and CDs-Au in water. (d) Blank studies of CDs and CDs-Au in water. ((a–d) Adapted with permission from ref. 300 Copyrights 2023, SPIE).

Incorporating the use of carbon dots with fluorescence lifetime imaging microscopy FLIM has advanced the field of cellular bio-imaging, particularly through the application of PEI-coated carbon dots (PEI-CDs). These PEI-CDs, synthesized *via* a one-step hydrothermal method, exhibit excellent biocompatibility and pH-responsive fluorescence due to the amine groups on the PEI surface. These characteristics make PEI-CDs promising candidates for cellular imaging, where their positive charge enables efficient electrostatic interactions with cellular components, particularly DNA within the nucleus. The fluorescence lifetime of PEI-CDs was found to be highly sensitive to pH changes, allowing for the monitoring of dynamic intracellular processes. The study utilized FLIM to assess the fluorescence lifetime of PEI-CDs under different pH conditions, showing their potential as effective pH sensors in living cells (Fig. 29a) presents FLIM images of the PEI-CDs under different pH conditions, highlighting their pH-dependent fluorescence lifetime variations. This demonstrates their potential as effective pH sensors in living cells, allowing for real-time monitoring of intracellular processes where pH plays a critical role, such as cell migration, apoptosis, and ion transport. Additionally, the conjugation of PEI-CDs with AuNPs formed a CDs-Au nanohybrid, which significantly enhanced the fluorescence intensity of the CDs through metal-enhanced fluorescence (MEF). The fluorescence intensity of PEI-CDs was notably higher after conjugation with AuNPs, illustrating the MEF effect, which improves the fluorescence properties and enhances their application in high-resolution bio-imaging. Fig. 29(b and c) show the fluorescence intensity and fluorescence lifetime measurements of both PEI-CDs and CDs-Au nanohybrids, demonstrating the enhanced optical properties of the hybrid structure. The fluorescence lifetime (FLT) of the PEI-CDs decreased slightly from 2.50 ns to 2.45 ns after complexation with AuNPs, indicating a more efficient interaction between the CDs and AuNPs.^300^ These findings suggest that PEI-CDs, particularly when integrated with gold nanoparticles, hold great potential for bio-labeling, targeted imaging, and therapeutic applications.

### 5.9 FLIM-CDS distinguish cellular autofluorescence

**Fig. 30:**
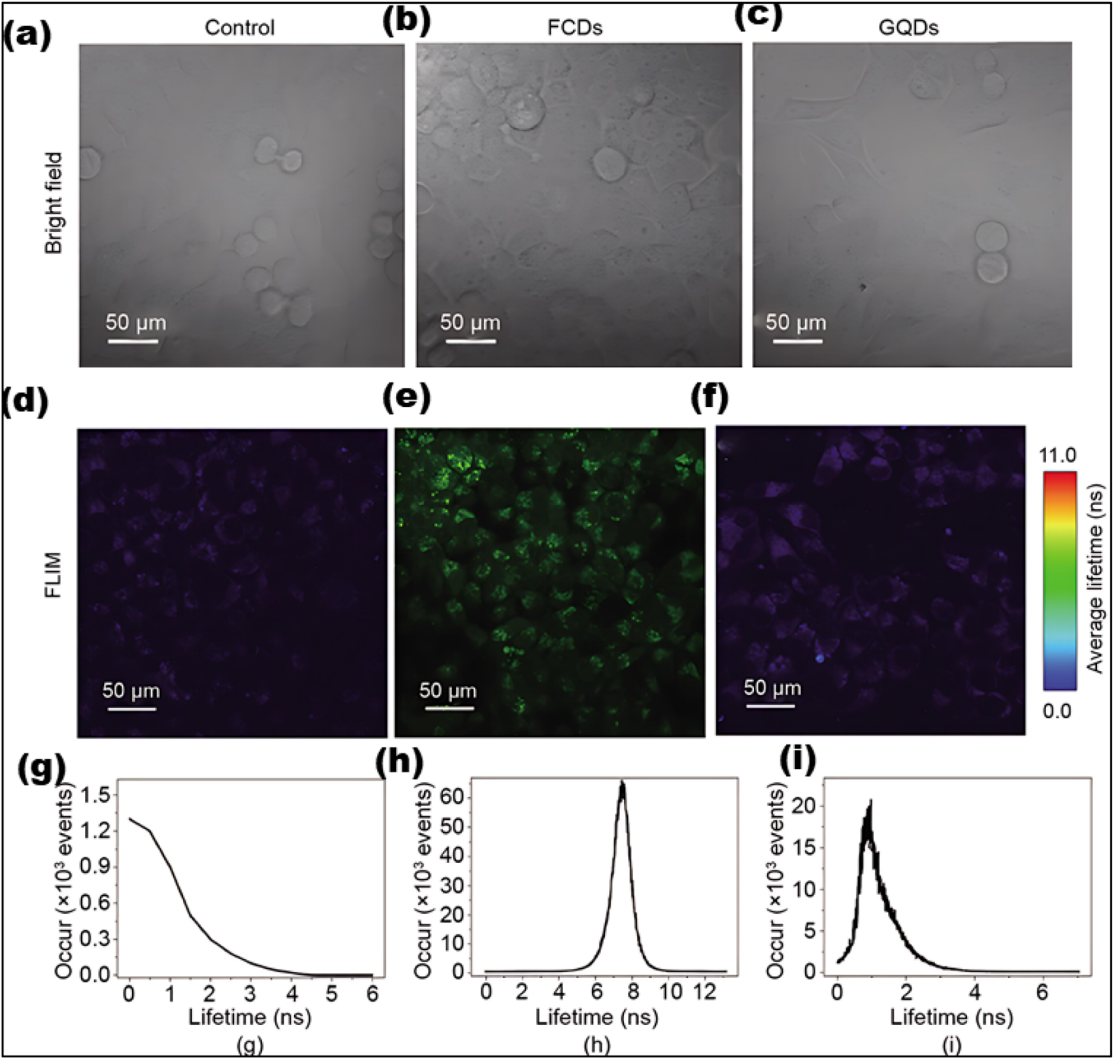
(a–c) Bright-field, (d–f) two-photon excited FLIM imaging, and (g–i) recovered lifetime histograms of control cells without FCD treatment (left column), experimental cells incubated with FCDs (middle column), and GQDs (right column), respectively. ((a–i) Adapted from ref. 304. Copyrights 2025 Elsevier Ltd).

CDs have been employed as fluorescent probes for cell imaging in the past due to their low cytotoxicity and intense fluorescence emission.^301^ Nevertheless, the majority of earlier research on the bioimaging of CDs in cells relied on enhancing the contrast between the cells and the backdrop by using the PL emission intensity of CDs. Unless there is a very high concentration of CDs in cells, the fluorescence signal from CDs is barely different from the cellular autofluorescence because CDs typically exhibit PL emissions in the blue-to-green region (400–500 nm), where cellular autofluorescence is also very intense under short-wavelength excitation. Researchers looked into the FCDs' possible uses for fluorescence lifetime imaging in living cells because of their extended fluorescence lifetime. When a fluorescent sample is activated by two-photon excitation,^302^ FLIM generates images based on the differences in fluorescence lifetimes. This allows one to see the contrast between materials that glow at the same wavelength but have different fluorescence decay rates.^303^ Furthermore, because NIR excitation light has a strong penetrating capability in tissues, the use of two-photon excitation (750 nm fs laser) is advantageous for possible deep-tissue imaging. Fig. 30 displays the FLIM pictures of graphene quantum dots (GQDs)145 with a short fluorescence lifetime (∼1 ns, right column), control cells (left column), and incubations with FCDs with a long fluorescence lifetime (∼7.2 ns, middle column). It is clear that the FCDs' extended fluorescence lifetime made it easier to distinguish the FCD fluorescence emission from the cells' autofluorescence. These findings demonstrate FCDs' enormous potential as intracellular imaging probes for FLIM imaging.

### 5.10 FLIM-CDs for niosome detection

Niosomes are very stable systems and biologically diverse substances having various applications in pharmaceuticals and as nano-carriers and nano-reactors. This was the first detailed report on the photophysical dynamics of fluorescent CDs in niosomes.

**Fig. 31:**
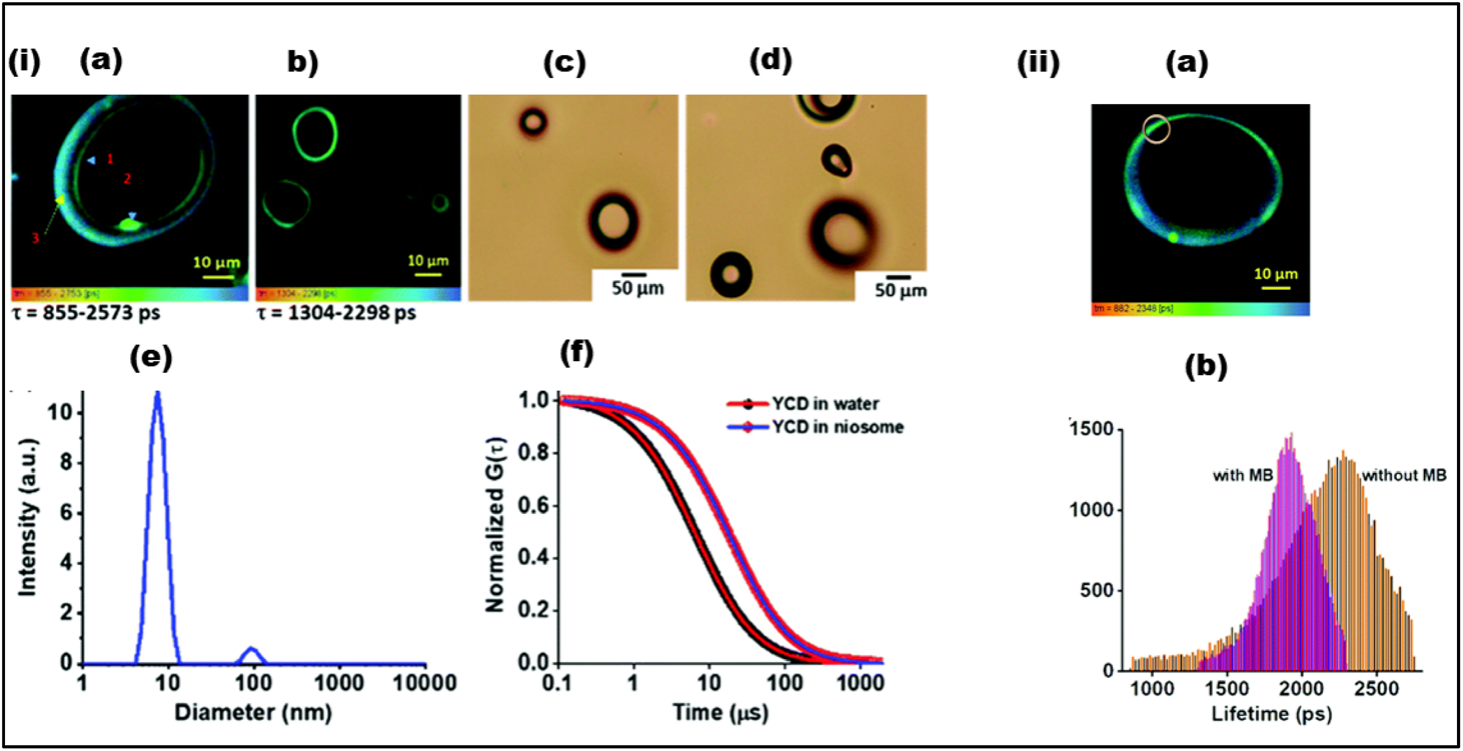
(i) (a and b) FLIM images, and (c and d) POM images of niosomes incorporated with YCDs; (e) DLS measurement showing the size distribution of the niosomes after sonication for 30 min, and (f) plot of the normalized correlation function (*G*(*τ*)) of YCDs with time obtained from the FCS measurements in bulk water and in niosomes. (ii) (a) FLIM images of the YCDs in niosomes with 75 µM MB (the ring selects a region for measuring the lifetimes), and (b) the lifetime histogram for the YCD–MB adduct and only YCD in the niosomes at 25 °C. ((a–f) Adapted with permission from ref. 305 Copyrights, RSC 2022).

Yellow emissive carbon dots (YCDs) were found in the surfactant bilayer of niosomes using the FLIM approach (Fig. 31i(a and b)). As a result, excited state durations could be correlated with time-correlated single photon counting (TCSPC) lifetimes. Polarised optical microscopy (POM) was used to confirm that the niosomes remained stable even after the addition of YCDs (Fig. 31i(c and d)). When the niosomes interacted with the YCDs, their shape did not change. The existence of YCDs in the bilayer was confirmed by FLIM pictures, which only appeared when fluorophores were present. The distribution of the YCDs in the niosomal bilayer was further examined using fluorescence correlation spectroscopy (FCS), which demonstrated a sluggish diffusion rate of YCDs inside the bilayer (Fig. 31i(e)). The fluorescence of the YCDs also displayed a bi-exponential decay (Fig. 31i(f)). As a result of the interactions between YCDs and the surfactant head groups, the diffusion time inside the niosomes increased from 7 µs in bulk water to roughly 20 µs. The observed delay in diffusion within the bilayer was supported by the predicted diffusion coefficients, which were 4.04 × 10^3^ µm^2^ s^−1^ in bulk water and 1.63 × 10^3^ µm^2^ s^−1^ in niosomes.

#### 5.10.1 Donor-detected (DD)-FLIM-FRET studies

When YCDs are included in niosomes, they cause two decaying components: one inside the multilamellar niosomal bilayers and one at the interface. Due to FRET and singlet oxygen, both components degrade more quickly when MB is added. While the slower component stays rather constant, the quicker component (likely YCDs at the interface) decays more. Understanding the FRET mechanism using lifetime imaging is made much easier with the help of the FLIM-FRET technology. Direct monitoring was done of the energy donor's (YCD) lifespan alterations within the niosomal bilayer.

The distribution of YCDs in two distinct niosomal regions was evident from the FLIM pictures; in one, their lifetime is somewhat shorter, while in the other, it is significantly longer. The discernible alterations was observed in the FLIM picture (Fig. 31ii(a)) upon the addition of methyl blue (MB) to the YCDs integrated inside the niosomal bilayers. Two decaying batches of YCDs that resembled those in the time-resolved ensemble studies were collected by us. Under typical circumstances, the two decaying components in the indicated area of Fig. 8a displayed decays of 807 and 3385 ps with 75 µM MB.

For the YCD-MB adducts and the YCDs integrated into the niosomal bilayer, the lifetime distribution clearly shifts in amplitude (Fig. 31ii(b)). We employed the DD-FLIM-FRET technique to explain our findings because the signal from MB could not be detected in the current setup. The multilayered niosome's interfacial region, which travels towards the polar aqueous regions and interacts with the YCDs, is where the FLIM image shows singlet oxygen production. Therefore, it is possible to think of the niosomes as carriers of singlet oxygen generators that can be designed to reach the intended targets. Furthermore, the findings show that adjusting the quantity of MB introduced to the system can affect the formation of singlet oxygen.^305^ The synthesized YCDs exhibit high fluorescence QY and photostability, making them promising for bio-imaging and staining. Incorporating them into niosomes enhances their QY to 91%, but reduces FRET efficiency due to bilayer interaction. These YCDs, paired with MB, show potential for NIR bio-imaging, FRET-based applications, and controlled photodynamic therapy (PDT).

## 6. Conclusions, challenges and perspectives

The integration of CDs into FLIM represents a significant advancement in cell imaging applications. It offers numerous advantages over conventional fluorescent probes. This review has explored the multifaceted potential of carbon dots, emphasizing their remarkable attributes such as biocompatibility, water solubility, and tunable fluorescence properties. From a synthetic standpoint, various methods for producing carbon dots have been explained, indicating the versatility of both “top-down” and “bottom-up” approaches. Analyzing its optical characteristics, the suitability of carbon dots for fluorescence-based applications is attributed to their UV-vis absorption, PL, fluorescence quantum yield, and exceptional photostability. Significantly, the tunability of their fluorescence properties through size, surface functionalization, and doping offers unprecedented flexibility in designing tailored imaging probes. Additionally, the ability of carbon dots to serve as sensors for detecting metal ions and ROS, as well as their potential for accurate targeting and imaging of cellular organelles, including lysosomes, mitochondria, and nuclei is emphasized. In the field of advanced imaging techniques, FLIM emerges as an essential tool, offering valuable fluorescence lifetime information for probing dynamic cellular processes. The synergy between carbon dots and FLIM suggest a new era in intracellular imaging and sensing, promising deeper insights into cellular functions and dynamics. Despite the promising applications of CDs in FLIM and cellular imaging, several challenges remain. One of the primary challenges is improving the penetration capabilities of CDs to enhance nucleous imaging and sensing. Current CDs often exhibit limited ability to penetrate deep cellular structures, restricting their applicability. Another significant challenge is addressing the long-term toxicity and environmental impact of CDs. While CDs are generally considered biocompatible, comprehensive studies on their long-term effects and environmental impact are necessary to ensure safe clinical applications. Photostability is another concern; prolonged exposure to light can lead to photo bleaching, limiting the duration of imaging sessions. Furthermore, achieving consistent quality and reproducibility in CDs synthesis is challenging due to variations in precursor materials and synthesis conditions. The processes of surface functionalization and doping, essential for tuning fluorescence properties, introduce additional complexity and variability in CD behavior.

Future research should focus on developing advanced synthesis techniques to produce CDs with consistent quality and reproducibility. To increase CDs' suitability for long-term imaging research, their photostability must be improved. Integrating CDs with other imaging modalities could provide more comprehensive imaging solutions, investing the unique properties of CDs for multi-modal imaging. Similarly exploring targeted delivery mechanisms to improve the specificity of CDs for various cellular components, including the nucleus, is essential for advancing their utility in detailed cellular imaging. Consequently, enhancing the real-time sensing capabilities of CDs, particularly for detecting dynamic changes in cellular environments, will open new avenues in biological research and medical diagnostics. Overall, the continued development and optimization of CDs for FLIM and cellular imaging hold significant promise for advancing our understanding of cellular processes and improving diagnostic techniques.

## Conflicts of interest

There are no conflicts to declare.

## Data Availability

As this is a review article, no new data were generated or analyzed. The content is based solely on previously published literature.

## References

[cit1] Toomre D., Bewersdorf J. (2010). Annu. Rev. Cell Dev. Biol..

[cit2] Xu H., Li Q., Wang L., He Y., Shi J., Tang B., Fan C. (2014). Chem. Soc. Rev..

[cit3] Lemon W. C., McDole K. (2020). Curr. Opin. Cell Biol..

[cit4] Refaat A., Yap M. L., Pietersz G., Walsh A. P. G., Zeller J., del Rosal B., Wang X., Peter K. (2022). J. Nanobiotechnol..

[cit5] Liu G. and Liu Z., in Computational Optical Imaging: Principle and Technology, ed. Z. Liu, X. Zhou and S. Liu, Springer Nature Singapore, Singapore, 2024, pp. 297–335, 10.11007/978-981-97-1455-1_11

[cit6] Sharma R., Singh M., Sharma R. (2020). Spectrochim. Acta, Part A.

[cit7] Xu P., Dong Z., Zhong S., Zhang Y.-H., Shen W. (2025). J. Innovative Opt. Health Sci..

[cit8] Jeong S., Widengren J., Lee J.-C. (2022). Nanomaterials.

[cit9] Schmidt E. L., Ou Z., Ximendes E., Cui H., Keck C. H. C., Jaque D., Hong G. (2024). Nat. Rev. Methods Primers.

[cit10] Li M., Li T., Wu F., Ren F., Xue S., Li C. (2024). Chemosensors.

[cit11] Ishikawa-Ankerhold H. C., Ankerhold R., Drummen G. P. C. (2012). Molecules.

[cit12] Pashayan N., Pharoah P. D. (2020). Science.

[cit13] Schiffman J. D., Fisher P. G., Gibbs P. (2015). Am. Soc. Clin. Oncol. Educ. Book.

[cit14] Juan C. A., Pérez de la Lastra J. M., Plou F. J., Pérez-Lebeña E. (2021). Int. J. Mol. Sci..

[cit15] Hatem E., El Banna N., Huang M.-E. (2017). Antioxid. Redox Signaling.

[cit16] Kennedy L., Sandhu J. K., Harper M.-E., Cuperlovic-Culf M. (2020). Biomolecules.

[cit17] Bhardwaj V., He J. (2020). Int. J. Mol. Sci..

[cit18] Hussain S., Mubeen I., Ullah N., Shah S. S. U. D., Khan B. A., Zahoor M., Ullah R., Khan F. A., Sultan M. A. (2022). BioMed Res. Int..

[cit19] Stefano A. (2024). Comput. Biol. Med..

[cit20] Kasban H. (2015). Int. J. Intell. Syst..

[cit21] Torrado B., Pannunzio B., Malacrida L., Digman M. A. (2024). Nat. Rev. Methods Primers.

[cit22] Suhling K. , Hirvonen L. M., Levitt J. A., Chung P.-H., Tregido C., Le Marois A., Rusakov D. A., Zheng K., Ameer-Beg S. and Poland S., Advanced Time-Correlated Single Photon Counting Applications, 2015, pp. 119–188

[cit23] Sun M.-J., Zhang Y.-C., Lin F.-R., Wang S., Liu L.-W., Qu J.-L. (2024). APL Photonics.

[cit24] Barroso M., Monaghan M. G., Niesner R., Dmitriev R. I. (2023). Adv. Drug Delivery Rev..

[cit25] Zhu H., Fan J., Du J., Peng X. (2016). Acc. Chem. Res..

[cit26] Chan J., Dodani S. C., Chang C. J. (2012). Nat. Chem..

[cit27] Zhang S., Qu Y., Zhang D., Li S., Tang F., Ding A., Hu L., Zhang J., Wang H., Huang K., Li L. (2024). Chem. Eur. J..

[cit28] Samanta S., Lai K., Wu F., Liu Y., Cai S., Yang X., Qu J., Yang Z. (2023). Chem. Soc. Rev..

[cit29] Lu S., Dai Z., Cui Y., Kong D.-M. (2023). Biosensors.

[cit30] Ceballos-Ávila D., Vázquez-Sandoval I., Ferrusca-Martínez F., Jiménez-Sánchez A. (2024). Biosens. Bioelectron..

[cit31] Saleh Mohammadnia M., Roghani-Mamaqani H., Mardani H., Rezvani-Moghaddam A., Hemmati S., Salami-Kalajahi M. (2022). Carbohydr. Polym..

[cit32] Ganguly S., Margel S. (2023). Pharmaceutics.

[cit33] Gómez-Virgilio L., Luarte A., Ponce D. P., Bruna B. A., Behrens M. I. (2021). Int. J. Mol. Sci..

[cit34] Adhikari M., Houhou R., Hniopek J., Bocklitz T. (2023). J. Exp. Theor. Anal..

[cit35] Liu X., Yu B., Shen Y., Cong H. (2022). Coord. Chem. Rev..

[cit36] Wu L., Li Z., Wang K., Groleau R. R., Rong X., Liu X., Liu C., Lewis S. E., Zhu B., James T. D. (2025). J. Am. Chem. Soc..

[cit37] Yao J., Yang M., Duan Y. (2014). Chem. Rev..

[cit38] Ge X., Sun L., Dang S., Liu J., Xu Y., Wei Z., Shi L., Zhang H. (2015). Microchim. Acta.

[cit39] Mazrad Z. A. I., Choi C. A., Kim S. H., Lee G., Lee S., In I., Lee K.-D., Park S. Y. (2017). J. Mater. Chem. B.

[cit40] Betzig E., Patterson G. H., Sougrat R., Lindwasser O. W., Olenych S., Bonifacino J. S., Davidson M. W., Lippincott-Schwartz J., Hess H. F. (2006). Science.

[cit41] Pedram P., Mahani M., Torkzadeh-Mahani M., Hasani Z., Ju H. (2016). Microchim. Acta.

[cit42] Cha C., Shin S. R., Annabi N., Dokmeci M. R., Khademhosseini A. (2013). ACS Nano.

[cit43] Georgakilas V., Perman J. A., Tucek J., Zboril R. (2015). Chem. Rev..

[cit44] Gaur M., Misra C., Yadav A. B., Swaroop S., Maolmhuaidh F. Ó., Bechelany M., Barhoum A. (2021). Materials.

[cit45] Zhang Z., Zhang J., Kwong G., Li J., Fan Z., Deng X., Tang G. (2013). Sci. Rep..

[cit46] Slepičková Kasálková N., Slepička P., Švorčík V. (2021). Nanomaterials.

[cit47] Himaja A. L., Karthik P. S., Singh S. P. (2015). Chem. Rec..

[cit48] Xu L., Li J., Li L., Luo Z., Xiang Y., Deng W., Zou G., Hou H., Ji X. (2021). Small.

[cit49] Dhenadhayalan N., Lin K.-C., Saleh T. A. (2020). Small.

[cit50] Rui S., Song L., Lan J., Wang D., Feng S., Lu J., Wang S., Zhao Q. (2023). Chem. Eng. J..

[cit51] Li H., Kang Z., Liu Y., Lee S.-T. (2012). J. Mater. Chem..

[cit52] Liu Z., Zou H., Wang N., Yang T., Peng Z., Wang J., Li N., Huang C. (2018). Sci. China Chem..

[cit53] Ðorđević L., Arcudi F., Cacioppo M., Prato M. (2022). Nat. Nanotechnol..

[cit54] Molaei M. J. (2019). RSC Adv..

[cit55] Al-Jumaili A., Alancherry S., Bazaka K., Jacob M. V. (2017). Materials.

[cit56] Ai L., Shi R., Yang J., Zhang K., Zhang T., Lu S. (2021). Small.

[cit57] Chen N., He Y., Su Y., Li X., Huang Q., Wang H., Zhang X., Tai R., Fan C. (2012). Biomaterials.

[cit58] Hötzer B., Medintz I. L., Hildebrandt N. (2012). Small.

[cit59] Verma A., Pandey V., Sherry C., Humphrey T., James C., Matteson K., Smith J. T., Rudkouskaya A., Intes X., Barroso M. (2025). Advanced Science.

[cit60] John B. K., Abraham T., Mathew B. (2022). J. Fluoresc..

[cit61] Saraswat S. K., Mustafa M. A., Ghadir G. K., Kaur M., Lozada D. F. G., Al-Ani A. M., Alshahrani M. Y., Abid M. K., Jumaa S. S., Alhameedi D. Y. (2024). Inorg. Chem. Commun..

[cit62] Alarfaj N.
A., El-Tohamy M. F., Oraby H. F. (2018). Int. J. Mol. Sci..

[cit63] Mindivan F., Göktaş M. (2023). Colloids Surf., A.

[cit64] Singh H., Bamrah A., Khatri M., Bhardwaj N. (2020). Mater. Today: Proc..

[cit65] Xu A., Wang G., Li Y., Dong H., Yang S., He P., Ding G. (2020). Small.

[cit66] Safardoust-Hojaghan H., Salavati-Niasari M., Amiri O., Rashki S., Ashrafi M. (2021). Ceram. Int..

[cit67] Gómez I. J., Sulleiro M. V., Dolečková A., Pizúrová N., Medalová J., Bednařík A., Preisler J., Nečas D., Zajíčková L. (2022). Mater. Chem. Front..

[cit68] Wu J.-B., Lin M.-L., Cong X., Liu H.-N., Tan P.-H. (2018). Chem. Soc. Rev..

[cit69] Rigodanza F., Burian M., Arcudi F., Đorđević L., Amenitsch H., Prato M. (2021). Nat. Commun..

[cit70] Li C. X., Yu C., Wang C. F., Chen S. (2013). J. Mater. Sci..

[cit71] Nie H., Li M., Li Q., Liang S., Tan Y., Sheng L., Shi W., Zhang S. X.-A. (2014). Chem. Mater..

[cit72] Zuo P., Lu X., Sun Z., Guo Y., He H. (2016). Microchim. Acta.

[cit73] Hettiarachchi S. D., Graham R. M., Mintz K. J., Zhou Y., Vanni S., Peng Z., Leblanc R. M. (2019). Nanoscale.

[cit74] Xia C., Zhu S., Feng T., Yang M., Yang B. (2019). Adv. Sci..

[cit75] Li W., Huang J., Li X., Zhao S., Lu J., Han Z. V., Wang H. (2021). Mater. Today Phys..

[cit76] Cao Y., Dong H., Yang Z., Zhong X., Chen Y., Dai W., Zhang X. (2017). ACS Appl. Mater. Interfaces.

[cit77] Tian P., Tang L., Teng K., Lau S. (2018). Mater. Today Chem.

[cit78] Boruah A., Saikia B. K. (2022). J. Fluoresc..

[cit79] Prabhakar N., Khan M. H., Peurla M., Chang H.-C., Hänninen P. E., Rosenholm J. M. (2017). ACS Omega.

[cit80] Bradac C., Gaebel T., Naidoo N., Sellars M., Twamley J., Brown L., Barnard A., Plakhotnik T., Zvyagin A., Rabeau J. (2010). Nat. Nanotechnol..

[cit81] Lettieri S. , d’Amora M. and Giordani S., Carbon Nanomaterials for Imaging, 2022, pp. 242–277

[cit82] Terada D., Sotoma S., Harada Y., Igarashi R., Shirakawa M. (2018). Bioconjugate Chem..

[cit83] Chan M. S., Liu L. S., Leung H. M., Lo P. K. (2017). ACS Appl. Mater. Interfaces.

[cit84] Ostadhossein F., Pan D. (2017). WIREs Nanomed. Nanobiotechol..

[cit85] Li J., Jiao Y., Feng L., Zhong Y., Zuo G., Xie A., Dong W. (2017). Microchim. Acta.

[cit86] Pan L., Sun S., Zhang A., Jiang K., Zhang L., Dong C., Huang Q., Wu A., Lin H., Beach D. (2015). Adv. Mater..

[cit87] Liu C., Wang R., Wang B., Deng Z., Jin Y., Kang Y., Chen J. (2018). Microchim. Acta.

[cit88] Li R., Wei F., Wu X., Zhou P., Chen Q., Cen Y., Xu G., Cheng X., Zhang A., Hu Q. (2021). Carbon.

[cit89] Zhao X., Li J., Liu D., Yang M., Wang W., Zhu S., Yang B. (2020). Iscience.

[cit90] Li D., Jing P., Sun L., An Y., Shan X., Lu X., Zhou D., Han D., Shen D., Zhai Y. (2018). Adv. Mater..

[cit91] Ghasemlou M., Pn N., Alexander K., Zavabeti A., Sherrell P. C., Ivanova E. P., Adhikari B., Naebe M., Bhargava S. K. (2024). Adv. Mater..

[cit92] Li F., Li Y., Yang X., Han X., Jiao Y., Wei T., Yang D., Xu H., Nie G. (2018). Angew. Chem..

[cit93] Ostadhossein F., Vulugundam G., Misra S. K., Srivastava I., Pan D. (2018). Bioconjugate Chem..

[cit94] Guan Q., Su R., Zhang M., Zhang R., Li W., Wang D., Xu M., Fei L., Xu Q. (2019). New J. Chem..

[cit95] Wu Z. L., Liu Z. X., Yuan Y. H. (2017). J. Mater. Chem. B.

[cit96] Jiang K., Wang Y., Li Z., Lin H. (2020). Mater. Chem. Front..

[cit97] Yan F., Zhang H., Yu N., Sun Z., Chen L. (2020). Sens. Actuators., B.

[cit98] Li D., Ushakova E. V., Rogach A. L., Qu S. (2021). Small.

[cit99] Mansuriya B. D., Altintas Z. (2021). Nanomaterials.

[cit100] Rao L., Zhang Q., Sun B., Wen M., Zhang J., Zhong G., Fu T., Niu X. (2022). Nanomaterials.

[cit101] Jiang K., Feng X., Gao X., Wang Y., Cai C., Li Z., Lin H. (2019). Nanomaterials.

[cit102] Tang L., Ai L., Song Z., Sui L., Yu J., Yang X., Song H., Zhang B., Hu Y., Zhang Y. (2023). Adv. Funct. Mater..

[cit103] Mo X., Lu Q., Li T., Tao X., Qi C., Zhou Y., Jiang Q., Ouyang Y. (2019). J. Mater. Sci..

[cit104] Wang X., Xu X.-C., Yang M., Jiang P., Zhao J., Jiang F.-L., Liu Y. (2019). New J. Chem..

[cit105] Wu S., Li W., Sun Y., Zhang X., Zhuang J., Hu H., Lei B., Hu C., Liu Y. (2019). J. Colloid Interface Sci..

[cit106] Xu X., Hu G., Mo L., Li Y., Wei H., Lei B., Zhang X., Hu C., Zhuang J., Liu Y. (2021). Nanoscale.

[cit107] Song G., Zhang Z., Fauconnier M.-L., Li C., Chen L., Zheng X., Zhang D. (2023). Nano Today.

[cit108] Jia J., Lu W., Cui S., Dong C., Shuang S. (2022). Mater. Today Chem..

[cit109] Xu X., Mo L., Li Y., Pan X., Hu G., Lei B., Zhang X., Zheng M., Zhuang J., Liu Y. (2021). Adv. Mater..

[cit110] Song H., Liu X., Wang B., Tang Z., Lu S. (2019). Sci. Bull..

[cit111] Kumari R., Sahu S. K. (2020). Langmuir.

[cit112] Smith J. T., Sinsuebphon N., Rudkouskaya A., Michalet X., Intes X., Barroso M. (2023). Biophys. Rep..

[cit113] Ding H., Wei J. S., Zhang P., Zhou Z. Y., Gao Q. Y., Xiong H. M. (2018). Small.

[cit114] Cao M., Zhao X., Gong X. (2022). Small.

[cit115] Wang B., Yu J., Sui L., Zhu S., Tang Z., Yang B., Lu S. (2021). Adv. Sci..

[cit116] Sun Z., Yan F., Xu J., Zhang H., Chen L. (2022). Nano Res..

[cit117] Wang B., Song H., Tang Z., Yang B., Lu S. (2022). Nano
Res..

[cit118] Wang J., Zheng J., Yang Y., Liu X., Qiu J., Tian Y. (2022). Carbon.

[cit119] Shen J., Zheng X., Lin L., Xu H., Xu G. (2023). ACS Appl. Nano Mater..

[cit120] Jv D.-j., Ji T.-h., Xu Z., Li A., Chen Z.-y. (2023). Food Chem..

[cit121] Xin N., Gao D., Su B., Zhou T., Zhu Y., Wu C., Wei D., Sun J., Fan H. (2023). ACS Sens..

[cit122] Ju B., Wang Y., Zhang Y.-M., Zhang T., Liu Z., Li M., Xiao-An Zhang S. (2018). ACS Appl. Mater. Interfaces.

[cit123] Liu Y., Zhou L., Li Y., Deng R., Zhang H. (2017). Nanoscale.

[cit124] Jiang L., Ding H., Lu S., Geng T., Xiao G., Zou B., Bi H. (2020). Angew. Chem..

[cit125] Liz-Marzán L. M., Artzi N., Bals S., Buriak J. M., Chan W. C., Chen X., Hersam M. C., Kim I.-D., Millstone J. E., Mulvaney P. (2023). ACS nano.

[cit126] Jiang C., Wu H., Song X., Ma X., Wang J., Tan M. (2014). Talanta.

[cit127] Vasimalai N., Vilas-Boas V., Gallo J., de Fátima Cerqueira M., Menéndez-Miranda M., Costa-Fernández J. M., Diéguez L., Espiña B., Fernández-Argüelles M. T. (2018). Beilstein J. Nanotechnol..

[cit128] Macairan J.-R., Jaunky D. B., Piekny A., Naccache R. (2019). Nanoscale Adv..

[cit129] Vale N., Silva S., Duarte D., Crista D. M., da Silva L. P., da Silva J. C. E. (2021). RSC Med. Chem..

[cit130] Hsu P.-C., Chen P.-C., Ou C.-M., Chang H.-Y., Chang H.-T. (2013). J. Mater. Chem. B.

[cit131] Qu S., Wang X., Lu Q., Liu X., Wang L. (2012). Angew. Chem., Int. Ed..

[cit132] Sun Y.-P., Zhou B., Lin Y., Wang W., Fernando K. S., Pathak P., Meziani M. J., Harruff B. A., Wang X., Wang H. (2006). J. Am. Chem. Soc..

[cit133] Su W., Wu H., Xu H., Zhang Y., Li Y., Li X., Fan L. (2020). Mater. Chem. Front..

[cit134] Hsu P. C., Chang H. T. (2012). Chem. Commun..

[cit135] Mitra S., Chandra S., Kundu T., Banerjee R., Pramanik P., Goswami A. (2012). RSC Adv..

[cit136] Win-Shwe T. T., Fujimaki H. (2011). Int. J. Mol. Sci..

[cit137] Das P., Ganguly S., Agarwal T., Maity P., Ghosh S., Choudhary S., Gangopadhyay S., Maiti T. K., Dhara S., Banerjee S. (2019). Mater. Chem. Phys..

[cit138] Ali H., Ghosh S., Jana N. R. (2020). WIREs Nanomed. Nanobiotechol..

[cit139] Chai L., Zhou J., Feng H., Tang C., Huang Y., Qian Z. (2015). ACS Appl. Mater. Interfaces.

[cit140] Yang L., Jiang W., Qiu L., Jiang X., Zuo D., Wang D., Yang L. (2015). Nanoscale.

[cit141] Zhang M., Zhao X., Fang Z., Niu Y., Lou J., Wu Y., Zou S., Xia S., Sun M., Du F. (2017). RSC Adv..

[cit142] Liu Y., Tian Y., Wang Y., Yang W. (2015). Adv. Mater..

[cit143] Wu L., Li X., Ling Y., Huang C., Jia N. (2017). ACS Appl. Mater. Interfaces.

[cit144] Hua X.-W., Bao Y.-W., Wu F.-G. (2018). ACS Appl. Mater. Interfaces.

[cit145] Cheng Y., Li C., Mu R., Li Y., Xing T., Chen B., Huang C. (2018). Anal. Chem..

[cit146] Zhang Y., Shen Y., Teng X., Yan M., Bi H., Morais P. C. (2015). ACS Appl. Mater. Interfaces.

[cit147] Kumawat M. K., Thakur M., Gurung R. B., Srivastava R. (2017). ACS Sustain. Chem. Eng..

[cit148] Tang Z., Lin Z., Li G., Hu Y. (2017). Anal. Chem..

[cit149] Petrakova V., Benson V., Buncek M., Fiserova A., Ledvina M., Stursa J., Cigler P., Nesladek M. (2016). Nanoscale.

[cit150] Shangguan J., He D., He X., Wang K., Xu F., Liu J., Tang J., Yang X., Huang J. (2016). Anal. Chem..

[cit151] Gao X., Ding C., Zhu A., Tian Y. (2014). Anal. Chem..

[cit152] Zhu X., Zhao T., Nie Z., Miao Z., Liu Y., Yao S. (2016). Nanoscale.

[cit153] He D., Yang X., He X., Wang K., Yang X., He X., Zou Z. (2015). Chem. Commun..

[cit154] Chen D., Zhao J., Zhang L., Liu R., Huang Y., Lan C., Zhao S. (2018). Anal. Chem..

[cit155] Zhang Z., Shi Y., Pan Y., Cheng X., Zhang L., Chen J., Li M.-J., Yi C. (2014). J. Mater. Chem. B.

[cit156] Qu Q., Zhu A., Shao X., Shi G., Tian Y. (2012). Chem. Commun..

[cit157] Sharma V., Kaur N., Tiwari P., Mobin S. M. (2018). J. Photochem. Photobiol. B.

[cit158] Jana J., Ganguly M., Das B., Dhara S., Negishi Y., Pal T. (2016). Talanta.

[cit159] Tadesse A., Hagos M., RamaDevi D., Basavaiah K., Belachew N. (2020). ACS Omega.

[cit160] Yang X. C., Li Q., Tang M., Yang Y. L., Yang W., Hu J. F., Pu X. L., Liu J., Zhao J. T., Zhang Z. J. (2020). ACS Appl. Mater. Interfaces.

[cit161] Fu C., Qian K., Fu A. (2017). Mater. Sci. Eng., C.

[cit162] Yang X.-C., Li Q., Tang M., Yang Y.-L., Yang W., Hu J.-F., Pu X.-L., Liu J., Zhao J.-T., Zhang Z.-J. (2020). ACS Appl. Mater. Interfaces.

[cit163] Francis L., Harrell A., Hallifax D., Galetin A. (2020). J. Pharm. Sci..

[cit164] Mohammadi S., Khajeh K., Taghdir M., Ranjbar B. (2021). Int. J. Biol. Macromol..

[cit165] Fang M., Zhuo K., Chen Y., Zhao Y., Bai G., Wang J. (2019). Anal. Bioanal. Chem..

[cit166] Chang S., Chen B. B., Lv J., Fodjo E. K., Qian R. C., Li D. W. (2020). Microchim. Acta.

[cit167] Sun Y., Qin H., Geng X., Yang R., Qu L., Kani A. N., Li Z. (2020). ACS Appl. Mater. Interfaces.

[cit168] Tong L., Wang X., Chen Z., Liang Y., Yang Y., Gao W., Liu Z., Tang B. (2020). Anal. Chem..

[cit169] Hallaji Z., Bagheri Z., Oroujlo M., Nemati M., Tavassoli Z., Ranjbar B. (2022). Microchim. Acta.

[cit170] Lu S., Guo S., Xu P., Li X., Zhao Y., Gu W., Xue M. (2016). Int. J. Nanomed..

[cit171] Wang P., Ji H., Guo S., Zhang Y., Yan Y., Wang K., Xing J., Dong Y. (2021). Chin. Chem. Lett..

[cit172] Havrdová M., Urbančič I., Bartoň Tománková K., Malina L., Štrancar J., Bourlinos A. B. (2021). Int. J. Mol. Sci..

[cit173] Datta K., Kozak O., Ranc V., Havrdova M., Bourlinos A., Šafářová K., Hola K., Tomankova K., Zoppellaro G., Otyepka M. (2014). Chem. Commun..

[cit174] Farley K. I., Surovtseva Y., Merkel J., Baserga S. J. (2015). Chromosoma.

[cit175] Penzo M., Montanaro L., Treré D., Derenzini M. (2019). Cells.

[cit176] Sen Gupta A., Sengupta K. (2017). Mol. Cell. Biol..

[cit177] Stimpson K. M., Sullivan L. L., Kuo M. E., Sullivan B. A. (2014). PLoS One.

[cit178] Cao C., Wei P., Li R., Zhong Y., Li X., Xue F., Shi Y., Yi T. (2019). ACS Sens..

[cit179] Feng R., Li L., Li B., Li J., Peng D., Yu Y., Mu Q., Zhao N., Yu X., Wang Z. (2017). RSC Adv..

[cit180] Kalji S.-O., Sadeghizadeh M., Khajeh K. (2025). Dyes Pigm..

[cit181] Yi S., Deng S., Guo X., Pang C., Zeng J., Ji S., Liang H., Shen X.-C., Jiang B.-P. (2021). Carbon.

[cit182] Li H., Ye S., Guo J., Wang H., Yan W., Song J., Qu J. (2019). Nano Res..

[cit183] Li X., Zhu T., Quan X., Yan H., Du Y., Yan R., Dong W.-F., Li L. (2025). Sens. Actuators, B.

[cit184] Liu Y., Zhou J., Wang L., Hu X., Liu X., Liu M., Cao Z., Shangguan D., Tan W. (2016). J. Am. Chem. Soc..

[cit185] Alqahtani T., Deore S. L., Kide A. A., Shende B. A., Sharma R., Chakole R. D., Nemade L. S., Kale N. K., Borah S., Deokar S. S. (2023). Mitochondrion.

[cit186] Wallace D. C. (2005). Annu. Rev. Genet..

[cit187] Zhang X., Chen L., Wei Y., Yang Y., Liu X., Du J., Li Q., Yu S. (2021). Fullerenes, Nanotubes Carbon Fullerenes, Nanotubes Carbon Nanostruct..

[cit188] Gong N., Ma X., Ye X., Zhou Q., Chen X., Tan X., Yao S., Huo S., Zhang T., Chen S. (2019). Nat. Nanotechnol..

[cit189] Gao G., Jiang Y.-W., Yang J., Wu F.-G. (2017). Nanoscale.

[cit190] Almanza A., Carlesso A., Chintha C., Creedican S., Doultsinos D., Leuzzi B., Luís A., McCarthy N., Montibeller L., More S. (2019). FEBS J..

[cit191] Yoshida H. (2007). FEBS J..

[cit192] Shuang E., Mao Q.-X., Wang J.-H., Chen X.-W. (2020). Nanoscale.

[cit193] Onal G., Kutlu O., Gozuacik D., Dokmeci Emre S. (2017). Lipids Health Dis..

[cit194] Liu M.-X., Ding N., Chen S., Yu Y.-L., Wang J.-H. (2021). Anal. Chem..

[cit195] Wang J., Guo Y., Geng X., Hu J., Yan M., Sun Y., Zhang K., Qu L., Li Z. (2021). ACS Appl. Mater. Interfaces.

[cit196] Lesani P., Singh G., Lu Z., Mirkhalaf M., New E. J., Zreiqat H. (2022). Chem. Eng. J..

[cit197] Liu Q., Niu X., Zhang Y., Zhao Y., Xie K., Yang B., He Q., Lv S., Li L. (2020). Nanoscale.

[cit198] Cui Y., Zhang C., Sun L., Hu Z., Liu X. (2015). Part. Part. Syst. Charact..

[cit199] Singh A. K., Singh V. K., Singh M., Singh P., Khadim S. R., Singh U., Koch B., Hasan S., Asthana R. (2019). J. Photochem. Photobiol., A.

[cit200] Gupta A., Nandi C. K. (2017). Sens. Actuators, B.

[cit201] Kumar A., Kumari A., Mukherjee P., Saikia T., Pal K., Sahu S. K. (2020). Microchem. J..

[cit202] Du F., Guo Z., Cheng Z., Kremer M., Shuang S., Liu Y., Dong C. (2020). Nanoscale.

[cit203] Liang C., Xie X., Zhang D., Feng J., Lu S., Shi Q. (2021). J. Mater. Chem. B.

[cit204] Pang L.-F., Sun Y.-C., Guo X.-F., Wang H. (2021). Sens. Actuators, B.

[cit205] Ren G., Hou X., Kang Y., Zhang R., Zhang M., Liu W., Li L., Wei S., Wang H., Wang B. (2020). Spectrochim. Acta, Part A.

[cit206] Deng Z., Li X., Liu H., He Y., Zhang J., Yuan J., Gao P., Jiang X., Yang Y., Zhong S. (2020). Microchim. Acta.

[cit207] Yu C., Li X., Zeng F., Zheng F., Wu S. (2013). Chem. Commun..

[cit208] Wang Q., Feng Z., He H., Hu X., Mao J., Chen X., Liu L., Wei X., Liu D., Bi S. (2021). Chem. Commun..

[cit209] Wang G.-G., Lv Q.-Y., Song X., Cui H.-F. (2021). Sens. Actuators, B.

[cit210] Yang S.-T., Cao L., Luo P. G., Lu F., Wang X., Wang H., Meziani M. J., Liu Y., Qi G., Sun Y.-P. (2009). J. Am. Chem. Soc..

[cit211] Jia Q., Zheng X., Ge J., Liu W., Ren H., Chen S., Wen Y., Zhang H., Wu J., Wang P. (2018). J. Colloid Interface Sci..

[cit212] Singh V., Kashyap S., Yadav U., Srivastava A., Singh A. V., Singh R. K., Singh S. K., Saxena P. S. (2019). Toxicol. Res..

[cit213] Kang Y.-F., Li Y.-H., Fang Y.-W., Xu Y., Wei X.-M., Yin X.-B. (2015). Sci. Rep..

[cit214] Liu W., Huang G., Su X., Li S., Wang Q., Zhao Y., Liu Y., Luo J., Li Y., Li C. (2020). ACS Appl. Mater. Interfaces.

[cit215] Rope R. and Breckenridge R., US Fish and Wildlife Service Lands Biomonitoring Operations Manual, EG and G Idaho, Inc., Idaho Falls, ID (United States), 1993

[cit216] Liu Y.-Y., Yu N.-Y., Fang W.-D., Tan Q.-G., Ji R., Yang L.-Y., Wei S., Zhang X.-W., Miao A.-J. (2021). Nat. Commun..

[cit217] Xiao L., Sun H. (2018). Nanoscale Horiz..

[cit218] Huang X., Zhang F., Zhu L., Choi K. Y., Guo N., Guo J., Tackett K., Anilkumar P., Liu G., Quan Q. (2013). ACS Nano.

[cit219] Li H., Yan X., Kong D., Jin R., Sun C., Du D., Lin Y., Lu G. (2020). Nanoscale Horiz..

[cit220] Boens N., Qin W., Basarić N., Hofkens J., Ameloot M., Pouget J., Lefevre J.-P., Valeur B., Gratton E., Vandeven M. (2007). Anal. Chem..

[cit221] Dai X.-Y., Huo M., Liu Y. (2023). Nat. Rev. Chem..

[cit222] Berezin M. Y., Achilefu S. (2010). Chem. Rev..

[cit223] López P. A., Blum S. A. (2024). ACS Catal..

[cit224] Samimi K., Pasachhe O., Guzman E. C., Riendeau J., Gillette A. A., Pham D. L., Wiech K. J., Moore D. L., Skala M. C. (2024). Cytometry, Part A.

[cit225] Gouzou D., Taimori A., Haloubi T., Finlayson N., Wang Q., Hopgood J. R., Vallejo M. (2024). Methods Appl. Fluoresc..

[cit226] Periasamy A. and Clegg R. M., FLIM Microscopy in Biology and Medicine, CRC Press, 2009

[cit227] Zhao J., Ni Y., Tan L., Zhang W., Zhou H., Xu B. (2024). Crit. Rev. Food Sci. Nutr..

[cit228] Roopnarine O., Yuen S. L., Thompson A. R., Roelike L. N., Rebbeck R. T., Bidwell P. A., Aldrich C. C., Cornea R. L., Thomas D. D. (2023). Sci. Rep..

[cit229] Joron K., Viegas J. O., Haas-Neill L., Bier S., Drori P., Dvir S., Lim P. S. L., Rauscher S., Meshorer E., Lerner E. (2023). Nat. Commun..

[cit230] Privitera A. P., Scalisi S., Paternò G., Cerutti E., D'Amico M., Pelicci P. G., Faretta M., Dellino G. I., Diaspro A., Lanzanò L. (2024). Commun. Biol..

[cit231] Popleteeva M., Haas K. T., Stoppa D., Pancheri L., Gasparini L., Kaminski C. F., Cassidy L. D., Venkitaraman A. R., Esposito A. (2015). Opt. Express.

[cit232] Rossetta A., Slenders E., Donato M., Zappone S., Fersini F., Bruno M., Diotalevi F., Lanzanò L., Koho S., Tortarolo G. (2022). Nat. Commun..

[cit233] Bénard M., Chamot C., Schapman D., Debonne A., Lebon A., Dubois F., Levallet G., Komuro H., Galas L. (2024). Life Sci. Alliance.

[cit234] Tortarolo G., Zunino A., Piazza S., Donato M., Zappone S., Pierzyńska-Mach A., Castello M., Vicidomini G. (2024). Adv. Photonics.

[cit235] Datta R., Heaster T. M., Sharick J. T., Gillette A. A., Skala M. C. (2020). J. Biomed. Opt..

[cit236] Bonilla P. A. and Shrestha R., in KRAS: Methods and Protocols, Springer, 2024, pp. 261–269

[cit237] Karrobi K., Tank A., Fuzail M. A., Kalidoss M., Tilbury K., Zaman M., Ferruzzi J., Roblyer D. (2023). Sci. Rep..

[cit238] Mate N., Satwani V., Pranav, Mobin S. M. (2025). Chem.–Asian J..

[cit239] Wallrabe H., Svindrych Z., Alam S. R., Siller K. H., Wang T., Kashatus D., Hu S., Periasamy A. (2018). Sci. Rep..

[cit240] Shirshin E. A., Shirmanova M. V., Gayer A. V., Lukina M. M., Nikonova E. E., Yakimov B. P., Budylin G. S., Dudenkova V. V., Ignatova N. I., Komarov D. V. (2022). Proc. Natl. Acad. Sci. U. S. A..

[cit241] Ouyang Y., Liu Y., Wang Z. M., Liu Z., Wu M. (2021). Nano-Micro Lett..

[cit242] Dvornikov A., Malacrida L., Gratton E. (2019). Methods Protoc..

[cit243] Ranjit S., Henriksen K., Dvornikov A., Delsante M., Rosenberg A., Levi M., Gratton E. (2020). Kidney Int..

[cit244] Crosignani V., Jahid S., Dvornikov A. S., Gratton E. (2014). Microsc. Res. Tech..

[cit245] Winkler U., Hirrlinger J. (2015). Neurochem. Res..

[cit246] Kolb D. A., Weber G. (1975). Biochemistry.

[cit247] Ma N., Digman M. A., Malacrida L., Gratton E. (2016). Biomed. Opt. Express.

[cit248] Shen B., Yan J., Wang S., Zhou F., Zhao Y., Hu R., Qu J., Liu L. (2020). Theranostics.

[cit249] Liggett J. R., Kang J., Ranjit S., Rodriguez O., Loh K., Patil D., Cui Y., Duttargi A., Nguyen S., He B. (2022). Front. Immunol..

[cit250] Tentori P., Signore G., Camposeo A., Carretta A., Ferri G., Pingue P., Luin S., Pozzi D., Gratton E., Beltram F. (2022). Nanoscale.

[cit251] Vallmitjana A., Dvornikov A., Torrado B., Jameson D. M., Ranjit S., Gratton E. (2020). Methods Appl. Fluoresc..

[cit252] Rahim M. K., Zhao J., Patel H. V., Lagouros H. A., Kota R., Fernandez I., Gratton E., Haun J. B. (2022). Anal. Chem..

[cit253] Frei M. S., Tarnawski M., Roberti M. J., Koch B., Hiblot J., Johnsson K. (2022). Nat. Methods.

[cit254] Vallmitjana A., Torrado B., Gratton E. (2021). Biomed. Opt. Express.

[cit255] Vu T., Vallmitjana A., Gu J., La K., Xu Q., Flores J., Zimak J., Shiu J., Hosohama L., Wu J. (2022). Nat. Commun..

[cit256] Weber G., Farris F. J. (1979). Biochemistry.

[cit257] Herrera-Ochoa D., Pacheco-Liñán P. J., Bravo I., Garzón-Ruiz A. (2022). ACS Appl. Mater. Interfaces.

[cit258] Rennick J. J., Nowell C. J., Pouton C. W., Johnston A. P. (2022). Nat. Commun..

[cit259] Belashov A., Zhikhoreva A., Salova A., Belyaeva T., Litvinov I., Kornilova E., Semenova I., Vasyutinskii O. (2024). Biochem. Biophys. Res. Commun..

[cit260] Shimolina L., Potekhina E., Druzhkova I., Lukina M., Dudenkova V., Belousov V., Shcheslavskiy V., Zagaynova E., Shirmanova M. J. B. J. (2022). Biophys. J..

[cit261] Belashov A., Zhikhoreva A., Salova A., Belyaeva T., Litvinov I., Kornilova E., Semenova I., Vasyutinskii O. J. B., R B. (2024). Communications.

[cit262] van der Linden F. H. (2021). Nat. Commun..

[cit263] Celli A., Sanchez S., Behne M., Hazlett T., Gratton E., Mauro T. (2010). Biophys. J..

[cit264] Meyer J., Untiet V., Fahlke C., Gensch T., Rose C. R. (2019). J. Gen. Physiol..

[cit265] Sarkar M., Maliekal T. T. (2024). Exp. Cell Res..

[cit266] Yang W. C., Li S. Y., Ni S., Liu G. (2024). Aggregate.

[cit267] Sun Y., Periasamy A. (2010). J. Biomed. Opt..

[cit268] Liang Z., Solano A., Lou J., Hinde E. (2024). Chromosoma.

[cit269] Eibl M., Karpf S., Weng D., Hakert H., Pfeiffer T., Kolb J. P., Huber R. (2017). Biomed. Opt. Express.

[cit270] Tortarolo G., Sun Y., Teng K. W., Ishitsuka Y., Lanzanó L., Selvin P. R., Barbieri B., Diaspro A., Vicidomini G. (2019). Nanoscale.

[cit271] Lanzano L., Coto Hernández I., Castello M., Gratton E., Diaspro A., Vicidomini G. (2015). Nat. Commun..

[cit272] Peng F., Ai X., Sun J., Ge X., Li M., Xi P., Gao B. (2024). Anal. Chem..

[cit273] Qi D. , Zhang S., Yao Y., Yao J., Jin C. and He Y., in Coded Optical Imaging, Springer, 2024, pp. 607–627

[cit274] Liu T., Stephan T., Chen P., Keller-Findeisen J., Chen J., Riedel D., Yang Z., Jakobs S., Chen Z. (2022). Proc. Natl. Acad. Sci. U. S. A..

[cit275] Esposito A., Gralle M., Dani M. A. C., Lange D., Wouters F. S. (2008). Biochemistry.

[cit276] Tantama M., Hung Y. P., Yellen G. (2011). J. Am. Chem. Soc..

[cit277] Harvey C. D., Ehrhardt A. G., Cellurale C., Zhong H., Yasuda R., Davis R. J., Svoboda K. (2008). Proc. Natl. Acad. Sci. U. S. A..

[cit278] Kuimova M. K., Yahioglu G., Levitt J. A., Suhling K. (2008). J. Am. Chem. Soc..

[cit279] Zhang Y., Birch D. J., Chen Y. (2011). Appl. Phys. Lett..

[cit280] Dahan M., Laurence T., Pinaud F., Chemla D., Alivisatos A., Sauer M., Weiss S. (2001). Opt. Lett..

[cit281] Giraud G., Schulze H., Bachmann T. T., Campbell C. J., Mount A. R., Ghazal P., Khondoker M. R., Ross A. J., Ember S. W., Ciani I. (2009). Int. J. Mol. Sci..

[cit282] Pai R. K., Cotlet M. (2011). J. Phys. Chem. C.

[cit283] Yaron P. N., Holt B. D., Short P. A., Lösche M., Islam M. F., Dahl K. N. (2011). J. nanobiotechnol..

[cit284] Okabe K., Inada N., Gota C., Harada Y., Funatsu T., Uchiyama S. (2012). Nat. Commun..

[cit285] Ripoll C., Orte A., Paniza L., Ruedas-Rama M. J. (2019). Sensors.

[cit286] Su W., Yang D., Wang Y., Kong Y., Zhang W., Wang J., Fei Y., Guo R., Ma J., Mi L. (2022). Nano Res..

[cit287] Rao C., Patel S. K., Prasad A., Garg N., Nandi C. K. (2021). ACS Appl. Bio Mater..

[cit288] Rao C., Sharma S., Garg R., Anjum F., Kaushik K., Nandi C. K. (2022). Biomater. Sci..

[cit289] Krasley A. T., Li E., Galeana J. M., Bulumulla C., Beyene A. G., Demirer G. S. (2024). Chem. Rev..

[cit290] Li J., Zheng S., Zhao X., Vomiero A., Gong X. (2025). Nano Energy.

[cit291] Momoh J., Kapsokalyvas D., Vogt M., Hak S., Kiessling F., van Zandvoort M., Lammers T., Sofias A. M. (2022). Adv. Drug Delivery Rev..

[cit292] Liu Y., Song Y., Zhang J., Yang Z., Peng X., Yan W., Qu J. (2021). ACS Appl. Mater. Interfaces.

[cit293] Pathak A., Navaneeth P., Gupta M., Pradeep A., Nair B. G., Suneesh P. V., Elangovan R., Sundberg L.-R., Marjomäki V., Babu T. S. (2023). Sens. Actuators, B.

[cit294] Li H., Wang H., Guo J., Ye S., Shi W., Peng X., Song J., Qu J. (2020). Sens. Actuators, B.

[cit295] Song J., Xu Z., Li H., Chen Y., Guo J. (2023). Int. J. Mol. Sci..

[cit296] Huang M., Liang X., Zhang Z., Wang J., Fei Y., Ma J., Qu S., Mi L. (2020). Nanomaterials.

[cit297] Zhang M., Su R., Zhong J., Fei L., Cai W., Guan Q., Li W., Li N., Chen Y., Cai L. (2019). Nano Res..

[cit298] Tian X., Zeng A., Liu Z., Zheng C., Wei Y., Yang P., Zhang M., Yang F., Xie F. (2020). Int. J. Nanomed..

[cit299] Guo Y., Huang Z., Wang L., Gao X., Chen Y., Lu F., Sun C., Li H., Li H., He Y. (2025). Anal. Chem..

[cit300] Pawar S., Duadi H., Fixler D. (2023). Nanomaterials.

[cit301] Wang D., Zhu L., Chen J.-F., Dai L. (2015). Nanoscale.

[cit302] Sun H., Zhang J., Zhang K. Y., Liu S., Liang H., Lv W., Qiao W., Liu X., Yang T., Zhao Q. (2015). Part. Part. Syst. Charact..

[cit303] Orte A., Alvarez-Pez J. M., Ruedas-Rama M. J. (2013). ACS Nano.

[cit304] Wang D., Wang Z., Zhan Q., Pu Y., Wang J.-X., Foster N. R., Dai L. (2017). Engineering.

[cit305] Chatterjee A., Sharma A. K., Purkayastha P. (2022). Nanoscale.

